# Endoscopic and Open Release Similarly Safe for the Treatment of Carpal Tunnel Syndrome. A Systematic Review and Meta-Analysis

**DOI:** 10.1371/journal.pone.0143683

**Published:** 2015-12-16

**Authors:** Haris S. Vasiliadis, Adriani Nikolakopoulou, Ian Shrier, Michael P. Lunn, Ruth Brassington, Rob J. P. Scholten, Georgia Salanti

**Affiliations:** 1 Orthopaedie Sonnenhof, Bern, Switzerland; 2 Department of Hygiene and Epidemiology, University of Ioannina School of Medicine, Ioannina, Greece; 3 Centre for Clinical Epidemiology, Lady Davis Institute for Medical Research, Jewish General Hospital, McGill University, Montreal, Canada; 4 Centre for Neuromuscular Disease, National Hospital for Neurology and Neurosurgery, Queen Square, London WC1N 3BG, United Kingdom; 5 Dutch Cochrane Centre and Julius Center for Health Sciences and Primary Care, University Medical Center Utrecht, Utrecht, The Netherlands; 6 Institute of Social and Preventive Medicine (ISPM) & Berner Institut für Hausarztmedizin (BIHAM) University of Bern, Bern, Switzerland; Universita' degli Studi di Napoli Federico II, ITALY

## Abstract

**Background:**

The Endoscopic Release of Carpal Tunnel Syndrome (ECTR) is a minimal invasive approach for the treatment of Carpal Tunnel Syndrome. There is scepticism regarding the safety of this technique, based on the assumption that this is a rather “blind” procedure and on the high number of severe complications that have been reported in the literature.

**Purpose:**

To evaluate whether there is evidence supporting a higher risk after ECTR in comparison to the conventional open release.

**Methods:**

We searched MEDLINE (January 1966 to November 2013), EMBASE (January 1980 to November 2013), the Cochrane Neuromuscular Disease Group Specialized Register (November 2013) and CENTRAL (2013, issue 11 in The Cochrane Library). We hand-searched reference lists of included studies. We included all randomized or quasi-randomized controlled trials (e.g. study using alternation, date of birth, or case record number) that compare any ECTR with any OCTR technique. Safety was assessed by the incidence of major, minor and total number of complications, recurrences, and re-operations.The total time needed before return to work or to return to daily activities was also assessed. We synthesized data using a random-effects meta-analysis in STATA. We conducted a sensitivity analysis for rare events using binomial likelihood. We judged the conclusiveness of meta-analysis calculating the conditional power of meta-analysis.

**Conclusions:**

ECTR is associated with less time off work or with daily activities. The assessment of major complications, reoperations and recurrence of symptoms does not favor either of the interventions. There is an uncertain advantage of ECTR with respect to total minor complications (more transient paresthesia but fewer skin-related complications). Future studies are unlikely to alter these findings because of the rarity of the outcome. The effect of a learning curve might be responsible for reduced recurrences and reoperations with ECTR in studies that are more recent, although formal statistical analysis failed to provide evidence for such an association. Level of evidence: I.

## Introduction

Carpal tunnel syndrome (CTS) is the most common compression neuropathy. Surgical treatment for CTS involves cutting the transverse carpal tunnel ligament (TCL) to release pressure on the median nerve. In traditional open surgery (Open Carpal Tunnel Release, OCTR) a wide incision is made in the wrist to fully visualise the ligament and surrounding structures. In 1989, Chow and Okutsu described separately two similar endoscopic techniques for carpal tunnel release (Endoscopic Carpal Tunnel Release, ECTR) [1,2]. ECTR is expected theoretically to have better outcomes in terms of pain, speed of healing and return to normal activities because it is minimally invasive and leaves structures overlying the TCL intact.

Contrary to expectations, several studies in the 1990s demonstrated an unacceptably high risk of complications, adding to skepticism about the new treatment [3–6]. Complication rates reported in the literature ranged from 2% to 35% [7,8]. The main argument supporting this criticism is that the surgeon is partially ‘blind’ during ECTR. This entails two main risks, these being failure to identify the distal edge of the TCL, resulting in incomplete release with subsequent recurrence and reoperation and damage to other structures, particularly to anatomical variants of the median nerve and branches [5,6,9]. Although more recent studies have described an equal complication rate with ECTR and conventional OCTR, controversy remains [10,11].

The aim of our study is to evaluate whether this skepticism is supported by evidence from randomized control trials by synthesizing data on the safety of ECTR in comparison to OCTR and investigate whether their relative safety has changed over time.

## Methods

### Data Sources and Searches

Our study is based on a recent systematic review undertaken by the Neuromuscular Disease Group of the Cochrane Collaboration, published in The Cochrane Library [12].We included all randomized or quasi-randomized controlled trials that compare any ECTR with any OCTR technique (with or without additional interventions such as lengthening of flexor retinaculum, internal neurolysis, epineurotomy or tenosynovectomy). Trials studying techniques with minimal incisions (mini-open techniques) were excluded. We accepted the definition of “mini-open technique” as given by the authors. Studies that only compared different endoscopic techniques against one another were also excluded.

No language restriction was applied. We included patients with clinical diagnosis of CTS as provided by the authors. No electrophysiological confirmation was required. Studies with patients with secondary CTS were excluded.

To identify relevant trials we searched MEDLINE (January 1966 to November 2013), EMBASE (January 1980 to November 2013), the Cochrane Neuromuscular Disease Group Specialized Register (November 2013) and CENTRAL (2013, issue 11 in The Cochrane Library) (S1 Appendix). We hand searched reference lists of included studies.

### Assessment of study limitations

The risk of bias in the included trials was assessed by two authors using the Cochrane Collaboration's Risk of Bias tool and is described in detail elsewhere [12,13].

### Data extraction

Safety was assessed by the incidence of major and minor complications, recurrences and re-operations. The total time needed to return to work or to return to daily activities was also assessed. When outcomes were provided at different time points, we extracted the total number of complications that were observed until the end of the study. Two authors (HV, GS) extracted data independently using standardized forms. A detailed taxonomy of complications classified into major and minor is provided in S2 Appendix.

### Data synthesis

We performed a random-effects meta-analysis for each outcome in Stata (StataCorp, 2011) using the inverse-variance method [14,15]. Treatment effects were summarized using odds ratio (OR) for binary outcomes and mean differences (MD) for continuous outcome. The assessment of the presence of statistical heterogeneity was based on the magnitude of the heterogeneity standard deviation (*τ*) estimated using the method of moments. We included predictive intervals to provide a range for the likely occurrence of complications in a new study [16]. We performed cumulative meta-analysis in order to display potential alteration of conclusions, how evidence was accumulated and whether the conclusions have changed over the years [17]. We also performed random-effects meta-regression using the year of the study publication as a covariate to examine potential time trends in the relative safety of the two interventions. To aid interpretation, the covariate was set to be the difference between year of publication in each study and the year of publication of the oldest study. Heterogeneity standard deviation (*τ*) was estimated using method of moments and the variance of the estimated coefficient was modified as suggested by Knapp and Hartung. For studies in which zero events have reported occurred in one of the two groups, a continuity correction of 0.5 was added. An alternative imputation method for handling rare events (the ‘reciprocal of the opposite arm size’) and Bayesian synthesis of data using a binomial likelihood and a bivariate model was also employed in a sensitivity analysis (S3 Appendix) [18]. [19].

As one person can have more than one complication, the total number of complications was modeled using a Poisson likelihood (S3 Appendix). We consider that our search strategy was complete and all efforts have been made to identify unpublished material; hence we a priori assumed that the probability of publication bias is low. Further indications of selective outcome reporting bias were evaluated via contour-enhanced funnel plots. [20]

The confidence in the conclusions of the analyses was judged following the GRADE system [21]. To evaluate whether the existing evidence is conclusive or future studies are likely to alter the meta-analysis result, we applied recently developed methodology based on the conditional power of an updated meta-analysis and extended funnel plots [22–25] (S3 Appendix).

The PRISMA checklist was followed when reporting this study (S1 PRISMA Checklist).

## Results

### Description of included studies

**Fig 1:**
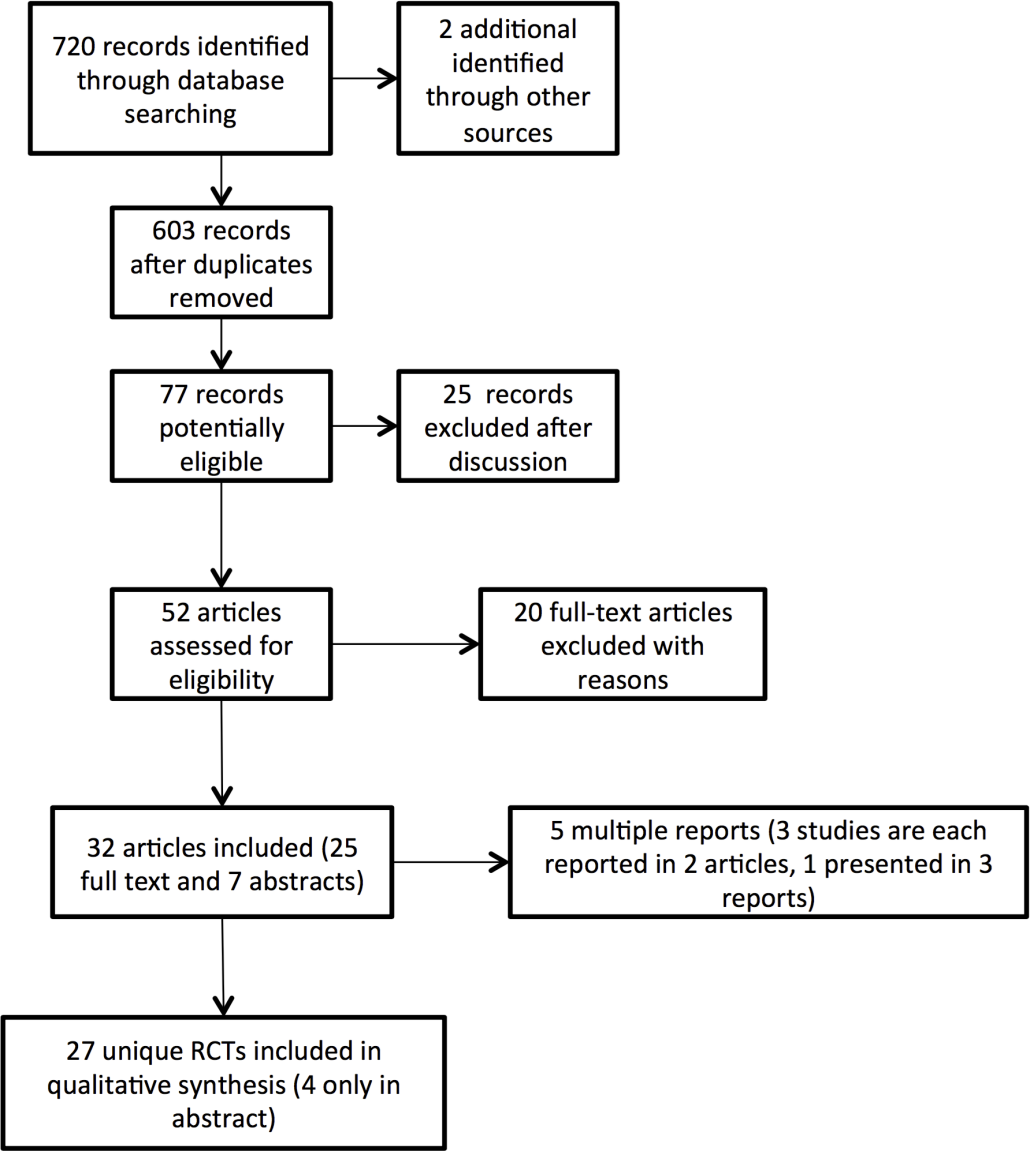
Study flow diagram.

Twenty-seven unique randomized control trials fulfilled the inclusion criteria and were selected [8,10,11,26–50] (Fig 1).One study was published in two manuscripts (in German and in English) and the two reports were considered jointly [28,51]. Atroshi et al. presented short-term and long-term results from the same group of patients in 2006 and 2009 [11,52]. For Foucher et al. results were retrieved from a manuscript and an abstract [35,53,54]. Sorensen et al. and Larsen et al. also represented the same study and data was extracted from both publications [44,50]. Four of the studies were presented only as an abstract [36,38,40,48].

### Techniques presented in the trials

In 10 studies, Agee’s technique was used [10,26,28,34,35,37,41–43,45]; an alternative one-portal endoscopic technique was performed in six studies [32,46–50]. Chow’s two-portal technique was performed in 8 studies [8,11,27,29–31,33,39]. In three studies the exact ECTR technique was not described [36,38,40]. Four studies included exclusively patients with bilateral CTS [34,36,38,45]. In 10 studies some (but not all) of the patients had bilateral CTS [10,26,27,29,32,33,39,40,46,47]. (S1 Table) summarizes the characteristics of the studies.

### Study limitations

The overall results from the risk of bias assessments are shown in S1 File. The majority of the evidence comes from studies at high or unclear risk of bias. The method of randomization was judged as appropriate in only eight studies. In Schafer et al. the treatment was allocated according to the day of the week (odd/even) (quasi-randomized trial) [43], while in the remaining 18 studies, the randomization method was not described. The allocation was adequately concealed in only four studies [11,29,47,50], inadequate in four [10,26,30,43] and not clearly described in the remaining 20 trials. The participants and personnel were not blinded in any of the included studies. This was anticipated as different incisions occur for open and endoscopic release.

Three studies were judged at high risk of attrition bias [27,30,31]. In Aslani et al. 9% of the participants were lost; the authors explain neither the distribution of this loss nor the reasons. Dumortier et al. also had an increased rate of missing data and in Eichhorn et al. ECTR patients that intraoperatively converted to open were excluded from the final analysis. For the rest of the studies insufficient information was provided in the manuscript to draw a safe conclusion. Only five of the studies were free of selective reporting [11,32,37,47,50]. In seven of the published reports it was clear that not all the predefined outcomes were sufficiently reported [10,29,31,39,42,43,45]. Generally, reporting was poor; no numerical outcome data was reported in [54] and no standard deviations were given nor could they be extracted in 6 studies [26,34,41,45,46,49].

Finally, only seven studies were clearly reported to be free from financial support from industry [10,11,27,29,41,47,50]. In Agee et al. the authors declared conflict of interest; no information was provided by the remainder of the studies. Baseline differences in important patient characteristics were found in two studies [33,42]. No baseline differences were found in Ejini et al., while the rest provided insufficient information.

### Safety and success-related outcomes

Only Incoll et al. and Tuzuner et al. did not report complications of any sort [38,47]. Fifteen studies reported on recurrence (3 with no events), 16 studies reported on reoperation (5 with no events), 25 studies reported on major complications (15 with no events) and 24 studies reported on minor complications (5 with no events).

#### Recurrences

**Fig 2:**
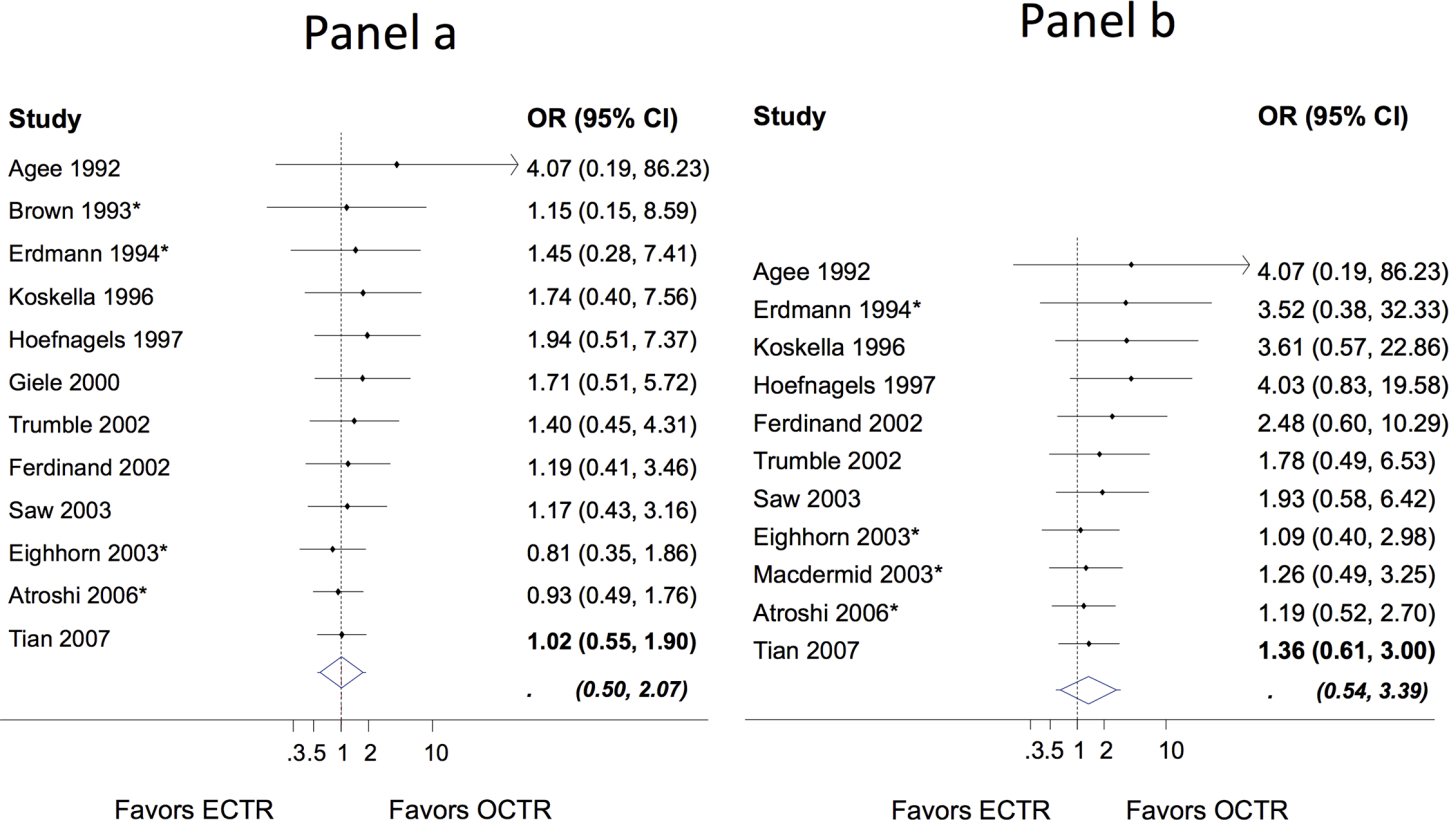
Cumulative odds ratios (OR) and 95% confidence interval (CI) for recurrence (panel a) and reoperations (panel b). The extended lines outside the diamond in the final meta-analytic summary shows the 95% prediction intervals. Asterisk indicates that the study has used a two-portal ECTR.

A total of 878 ECTR and 806 OCTR cases were recorded in the 15 studies that provided events for the “recurrence “outcome. From those, 24 ECTR and 19 OCTR cases recurred. The meta-analysis revealed no difference between endoscopic and open release (OR 1.02; 95% CI 0.55 to 1.90) (Fig 2A), (S2 File). The heterogeneity standard deviation *τ* was estimated to be zero and the 95% prediction interval (0.50 to 2.07) indicates that future studies may show a two-fold benefit for either surgical procedure. The cumulative meta-analysis illustrates a decreased OR for ECTR vs open release since 1992, suggesting a learning effect over the years. The meta-regression suggested that for every year after 1992 the OR has a relative decrease of 3% (regression coefficient on OR scale 0.97 (95% CI 0.84 to 1.14); suggesting that on average more recent studies tend to decrease the relative safety of OCTR. However this association did not reach statistical significance.

#### Reoperations

The outcome was explicitly considered in 16 studies. Eleven studies reported 28 reoperations from 1596 operations (20 ECTR and 8 OCTR reoperations from 869 ECTR and 727 OCTR cases). ECTR had to be converted to open release in 15 cases, in 5 studies, due to intraoperative difficulties [11,28,35,37,42]. In three cases re-operations were prompted by the injury of the superficial palmar branch (one in the ECTR [29] which was converted to open and two in the OCTR [33,34]).

No differences in the incidence of reoperation were found between ECTR and OCTR release (OR 1.36; OR 0.61 to 3.00) (Fig 2B). There was no between study heterogeneity (*τ* = 0) while based on the prediction interval, it is unlikely for a future study to show a beneficial effect of one technique over the other (0.54 to 3.39). The cumulative forest plot in Fig 2B shows that the initial insignificant advantage of OCTR was moderated with the conduct of new studies suggesting a learning effect of ECTR.

The meta-regression suggested that for every year after 1992 the OR has a relative decrease of 8% (regression coefficient on OR scale 0.92; 95% CI 0.75 to 1.12); suggesting that on average more recent studies tend to decrease the relative safety of OCTR. However, this association did not reach statistical significance.

#### Major complications

Major complications were found very infrequently. From 25 studies reporting major complications as an outcome, only 10 of them reported events of major complications. In total, 12 ECTR and 12 OCTR cases experienced a major complication (from 1366 ECTR and 1199 OCTR cases treated).

Agee 1992 reported one injury to the deep motor branch of the ulnar nerve in an OCTR-treated patient [26] while Eriji report a case treated with ECTR who developed symptoms compatible with common digital nerve injury [32]. Atroshi et al. had no nerve, vascular, or tendon injuries, and no wound complications at one year in a follow up study. However 5 ECTR and 3 OCTR patients with moderate or severe pain were reported at 5 years [11,52]. Complex regional pain syndrome (CRPS) was the most frequently found major complication; two cases (one mild and one severe) were recorded in the 25 hands treated with open release in Tuzuner et al., two cases (one in each group) were reported by Benedetti et al., one case reported in the ECTR group of Foucher et al. [47][51,54] and two cases with symptoms consistent with CRPS in the OCTR group reported in Malhotra et al. [41]. Persistent mild or severe symptoms of CRPS were judged as a major complication. In Hoefnagels et al. an endoscopic knife broken intraoperatively and one other an increased postoperative hypesthesia that required revision surgery.

**Fig 3:**
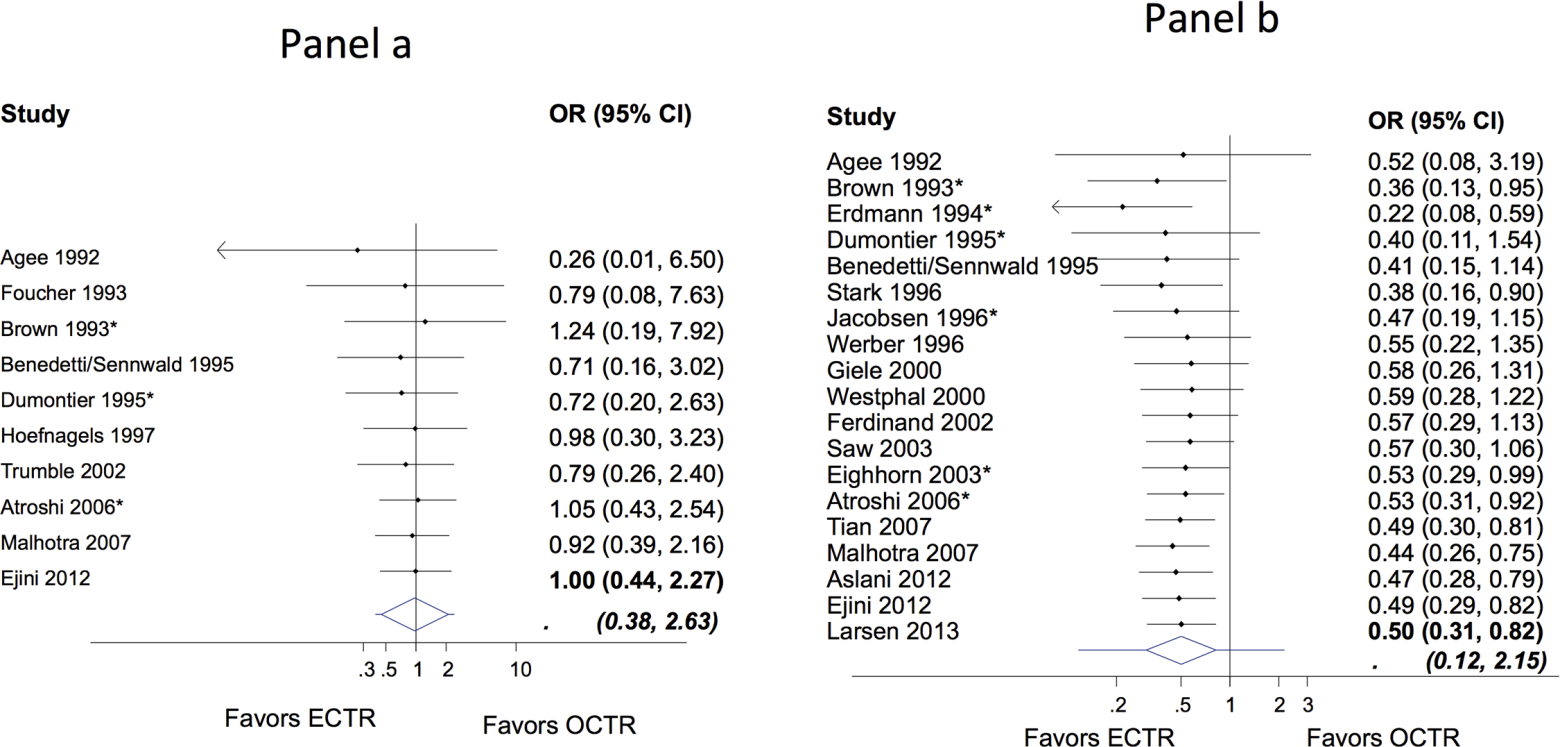
Cumulative odds ratios (OR) and 95% confidence interval (CI) for minor (panel a) and major (panel b) complications. The extended lines outside the diamond in the final meta-analytic summary shows the 95% prediction intervals. Asterisk indicates that the study has used a two-portal ECTR.

Meta-analysis of the 25 studies did not reveal any differences between ECTR and OCTR (OR 1.00; 95% CI 0.44 to 2.27) although uncertainty in the estimation was important (Fig 3A). No important heterogeneity was observed (*τ* = 0) and consequently the predictive interval is slightly wider than the confidence interval (0.38 to 2.63). There is no evidence of a trend of the effect estimates over years (Fig 3A). Accounting for year of publication in a meta-regression model resulted in statistically non-significant association; for every year after 1992 the OR has a relative increase of 3% (regression coefficient on OR scale 1.03; 95% CI 0.88 to 1.20); suggesting that on average more recent studies tend to increase the relative safety of OCTR.

#### Minor complications

In total, there were 183 minor complications from 2442 hands in the 24 studies that reported this outcome (63 ECTR and 120 OCTR minor complications out of the 1275ECTR and 1167 OCTR cases). The meta-analysis revealed that ECTR resulted on average in a lower rate of minor complications when compared with OCTR (OR 0.50; 95% CI 0.31, 0.82) (Fig 3B). The summary effect indicates that ECTR is associated with an average relative decrease in odds of minor complication of 50% compared to OCTR. However, there is large between study heterogeneity (*τ* = 0.64) and the 95% predictive interval (0.12 to 2.15) indicates that ECTR may not always be beneficial in future studies. No trend of the effect estimates is evident over years (Fig 3B). The meta-regression yielded a non-statistically significant regression coefficient; for every year after 1992 the OR has a relative increase of 1% (regression coefficient on OR scale 1.01; 95% CI 0.93 to 1.10). Heterogeneity standard deviation *τ* was estimated at 0.69 suggesting that the year of publication cannot explain the between-studies variability.

Further analysis of minor complications revealed that ECTR was associated with a higher rate of transient nerve problems (for example neurapraxia, numbness, paraesthesia) compared to OCTR and that OCTR was associated with a higher rate of wound problems compared to ECTR. From the 24 studies that reported minor complications, 13 reported events with transient neuropraxia with spontaneous symptom relief in the first postoperative weeks. The meta-analysis suggested that the odds for transient neuropraxia after ECTR is on average 2.42 times the odds after OCTR (OR 2.42; 95% CI 1.22 to 4.80) (Figure A in S2 File). Fourteen studies reported cases suffering from wound or scar problems, including postoperative infections, hypertrophic scarring or scar tenderness. Synthesis of the data revealed that the odds for wound or scar problems is 76% lower after ECTR when compared to conventional OCTR (OR 0.24; 95% CI 0.15 to 0.40) (Figure B in S2 File).

#### Total complications

Twenty-five studies provided data for at least one complication (minor, major or recurrence and reoperation). In total, 278 complications (119 ECTR and 159 OCTR) were reported in 7,227 patients-years. One study was excluded from the analysis as it did not provide the length of follow-up (Mackenzie 2000). The summary estimate derived from Poisson meta-analysis suggested that the rate of total complications does not differ between ECTR and OCTR (rate ratio 0.82; 95% CI 0.52, 1.51) (rate ratio greater than 1 favors OCTR). The heterogeneity standard deviation was estimated at 0.85 with a 95% predictive interval for the rate ratio of (0.12 to 6.55) suggesting that a new study could favor any of the two interventions.

#### Time to return to work or to daily activities

**Fig 4:**
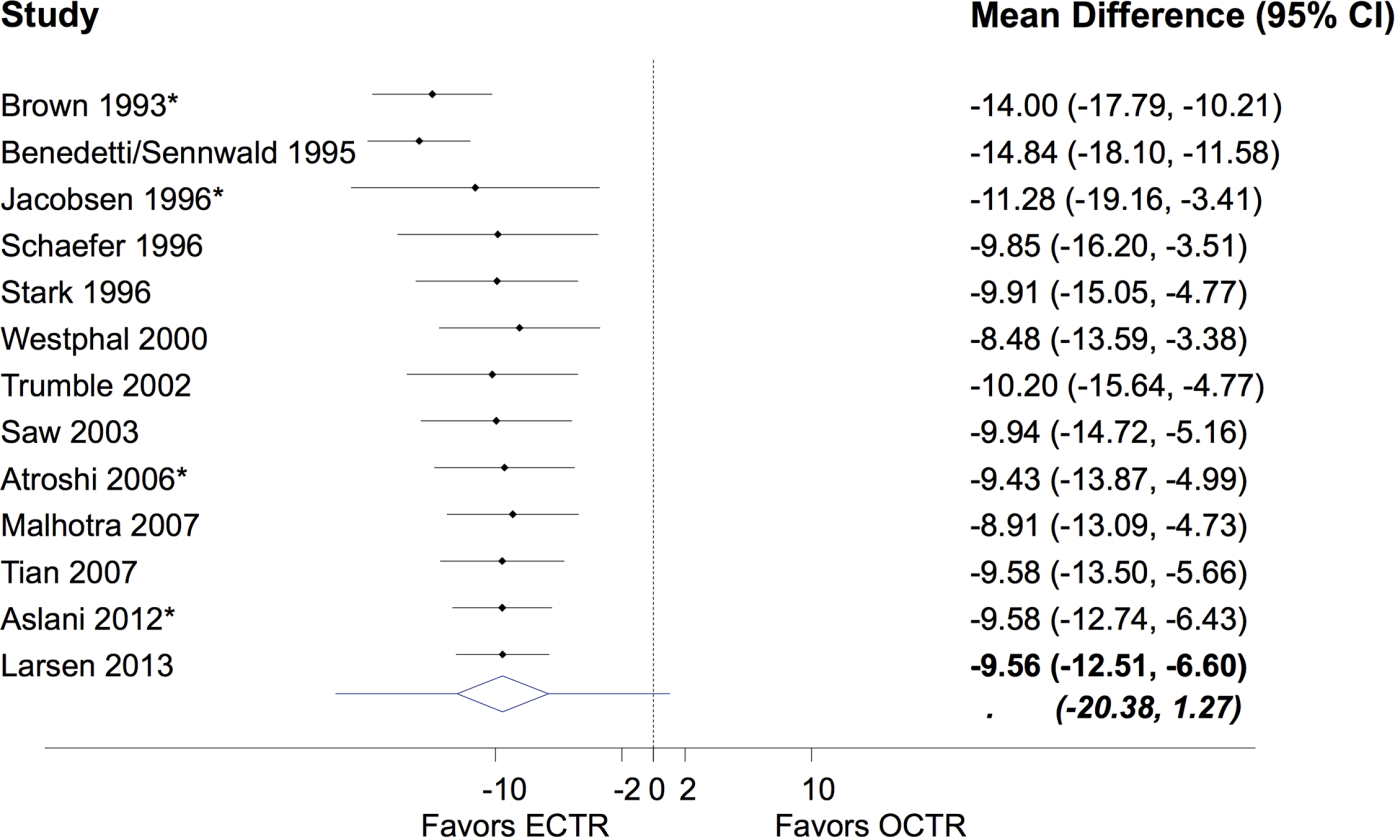
Cumulative mean difference (MD) and 95% confidence interval (CI) for complications time to return to work or daily activities. The extended lines outside the diamond in the final meta-analytic summary shows the 95% prediction intervals. Asterisk indicates that the study has used a two-portal ECTR.

In six studies that reported this outcome but did not provide standard deviations we assumed a value identical to Saw et al. as the most representative of the studies. The analysis revealed a statistically and clinically significant reduction of time out of work or daily activities with ECTR; patients treated with ECTR returned to work or to daily activities on average 10 days earlier that those in the OCTR group (mean difference -9.56; 95% CI -12.51 to -6.60) (Fig 4). Divergences between studies resulted in a large between study heterogeneity (*τ* = 4.68). A possible explanation for this large variability is that the flexibility and nature of work and daily activities might be substantially different in the included study settings. This increased diversity is also presented in the predictive interval which is rather wide (-20.38 to 1.27) showing that future studies are likely to show beneficial effect either for ECTR or OCTR. However, the great majority of the expected relative effects in future studies are in favor of ECTR. There is no statistically significant evidence of a trend for the effect estimates over years (Fig 4). The meta-regression did not reveal any important association between time to return to work and year of study publication (regression coefficient 0.07; 95% CI = -0.51 to 0.65) and failed to explain the residual heterogeneity (*τ* was estimated at 5.34).

#### Sensitivity analysis

Crude forest plots for the outcomes recurrences, reoperations, major complications, minor complications and time to return to work/daily activities are displayed in Figures C to G in S2 File. Alternative imputation approaches for handling rare events and Bayesian synthesis of the data using the binomial likelihood or the bivariate model showed similar results for the analyses of recurrences, reoperations, major and minor complications (Figures H to K in S2 File,). Given the low to very low quality of evidence, we performed a post-hoc sensitivity analysis excluding studies with high risk of bias regarding allocation concealment for the outcomes recurrences, reoperations, major and minor complications. Results were consistent to the main analysis and only minimal differences occurred (Figures A to D in S3 File).

### Is the current evidence convincing and conclusive?

The credibility of the results as assessed by GRADE is presented in S2 Table. The low number of events in the dichotomous outcomes and the high or moderate risk of bias in the included studies for all outcomes are the main reasons for downgrading the evidence to low and very low levels. No convincing evidence of small study effects was found. Although small studies tended to favor the safety of OCTR (Figures L to Q in S2 File), these generally represented the early studies. The cumulative meta-analysis suggesting a learning effect with ECTR would be the most likely explanation for any small study effect that occurred. Overall, the confidence in the available evidence is low to very low.

We calculated the additional sample size required in a future study which, when added to the present meta-analysis would show that the result for each non-significant outcome turns statistically significant in favour of ECTR (Figures P to X in S2 File.). For all outcomes the power is very low (smaller than 50%) even with the inclusion of 5000 additional patients. Therefore, the probability of detecting statistically significant results in a future meta-analysis for recurrence, reoperations and major complications is very low and the current conclusions are unlikely to change. Note that the required sample size to change the meta-analysis conclusions in favour of OCTR would not materially change for the outcomes recurrence and major complications whereas it would be smaller for the outcome reoperation. The funnel plots in Figures Y to AB in S2 File. show the potential conclusions of an updated meta-analysis if the findings of a new trial were to be added to the existing meta-analysis. Confirming the analysis using the conditional power, visual inspection of the funnel plots implies that it is unlikely for a new trial to change the conclusions of the meta-analyses in terms of statistical significance for recurrence, reoperations, major complications and minor complications.

Although it is unlikely that a very large new study will change the conclusions of the current meta-analysis, the credibility of the included studies and their high risk of bias call for more high-quality evidence to draw firm conclusions about the relative safety of the two procedures.

## Discussion

To our knowledge, this is the most comprehensive and up-to-date systematic review of randomized trials regarding the complications of endoscopic and open release for the treatment of CTS. We employed a variety of synthesis methods and we demonstrated that there is no evidence suggesting that either of the techniques is safer than the other. There seems to be a small advantage of ECTR with respect to minor complications and a clear advantage in terms of days to return to work or to daily activities. These conclusions are unlikely to change in future studies.

The most important limitation of our study is that we included only RCTs. Due to small sample size and the nature of the outcome (complications are generally rare), the power of our analysis is low. It is of interest to compare the results of the present study to a meta-analysis of cohort studies although any association found in the latter could be due to the biases operating in observational studies. A systematic review including 54 publications of any level of evidence (9516 endoscopic and 1203 open releases from case reports to randomized trials) published 15 years ago suggested that ECTR was comparable to OCTR in terms of the incidence of irreversible nerve damage. However, case reports might indicate a small risk of unacceptable complications after ECTR, like transection of the median nerve[3]. A similar review included 68 articles published until 2001 [55] and also concluded that ECTR and OCTR had similar complication rates. An interesting finding was that, although ECTR was first described in 1989, more than twice as many complications of ECTR than OCTR were reported in the literature between 1966 to 2001 [55]. The debate about the safety and complications of ECTR as a novel technique had possibly led to over-reporting its complications relative to those of OCTR, suggesting a high reporting bias. Another explanation might be the lack of experience in performing a technically demanding novel technique such as the ECTR, that led to an increased rate of complications.

ECTR appears to result in a higher incidence of transient neuropraxias. The insertion of the cutting device intraoperatively may compress or stretch the diseased median nerve, contributing to a transient apraxia [56]. In almost all the cases the reported neuropraxias are transient, subside within days to 2–3 weeks and do not affect the final outcome of the carpal tunnel release.

The incision of the TCL during ECTR is not accompanied by injury of the overlying subcutaneous nerves of the palm. This probably explains the fewer painful scars following endoscopic techniques. By contrast, the palmar incision used in OCTR results in an incision of skin and subcutaneous tissue, increasing the risk of painful neuromas. Use of the hand and repetitive injury of the extended scar may also add to the higher risk for hypertrophic or hypersensitive scars after OCTR. The extended incision in the palm may also extend the immobilization time and increase the postoperative pain, both factors contributing to a higher incidence of CRPS.

ECTR patients return to work or daily activities earlier than those treated with an open technique. The greater surgical trauma of open release is associated with increased pain [57]. Sanati et al also demonstrate a superiority of minimally invasive techniques over conventional open release in recovery time; however, the authors included both mini-open techniques and ECTR under the group “minimal invasive techniques” [58]. They also highlighted the inconsistency of return to work as an outcome. This inconsistency is likely to arise from different definitions used, social and economic factors (the generosity of the public system in terms of sick leave), and the occupations of trial participants. However, the effect of such inconsistency is rather small when only randomized controlled trials are considered, as in our study.

A theoretical disadvantage of ECTR is of not directly identifying the distal edge of the TCL, which may potentially lead to incomplete release and recurrence [59,60]. However, there is still controversy regarding the importance of the division of the distal end of the TCL. [61][62]. Cobb and Cooney showed that dividing the distal 4mm of the TCL had no impact on carpal arch widening when compared with incomplete division of the ligament [63]. The findings of our study suggest that ECTR is equally effective clinically, presumably resulting in a complete release of the carpal tunnel. It is unclear though if this is correlated to a complete division of the TCL.

Similar outcomes were also found in a recent systematic review that assessed 15 randomized trials. The authors based on a limited number of studies, confirmed the higher rate of irreversible nerve problems and less skin issues after ECTR, followed by a faster return to work [64].

It has been suggested that ECTR has a relatively high learning curve [65,66]. Although ECTR is considered a safe procedure, meticulous training is mandatory before starting clinical practice. Instructional courses and practice in cadavers are highly recommended and have been shown to reduce the incidence of complications [4,60]. Tight access to the carpal tunnel for cannula assembly is a frequent technical difficulty, which is hazardous to the median nerve. Impaired visualization of the TCL may also increase the risk of complications. When surgeons encounter difficulties they should, particularly if less experienced, consider discontinuing and converting ECTR to conventional OCTR [56]. In this review, only 13 conversions to open release were mentioned. We detected that recurrences and re-operations tend to decrease in the ECTR arm compared to OCTR in more recent studies. This might be the results of the learning curve, small study effects, publication bias in earlier trials or might be due to the fact that modifications of the ECTR aimed at correcting early deficiencies were introduced with time. The use of one or two portals was also associated with time and might explain the changes in the relative efficacy over the years. Studies involving the one-portal technique tended to be slightly older (median year 2000; interquantile range 1996 to 2007) than studies with two portals (2 median 003; interquantile range 1995 to 2006) for those used two-portal ECTR. Overall, based on the available evidence, patients to undergo a carpal tunnel release should choose the procedure considering their relative efficacy and the skills of the surgeon rather than concerns about their relative safety.

## Conclusion

Evidence of low quality suggests that ECTR is associated with less time spent out of work or daily activities; patients treated with ECTR return to work on average 10 days earlier than those treated with OCTR. The quality of evidence is low to very low regarding complications, reoperations and recurrence of symptoms and does not favor either of the interventions; future studies are unlikely to alter this conclusion primarily due to the rare nature of the outcomes. Low quality evidence suggests an uncertain advantage of ECTR with respect to minor complications. The effect of a learning curve might be responsible for reduced recurrences and reoperations with ECTR in studies that are more recent.

## Supporting Information

S1 PRISMA Checklist (DOC)

S1 Appendix Search Strategy. (DOCX)

S2 Appendix Definitions of Complications as assessed in the Review. (DOCX)

S3 Appendix Details regarding the models used as well as supplementary statistical analyses. (PDF)

S1 File Figure A. Risk of bias graph: review authors’ judgments about each risk of bias item presented as percentages across all included studies. Figure B. Risk of bias summary: review authors’ judgments about each risk of item for each included study. (PDF)

S2 File Figure A. Random effects meta-analysis for transient neuropraxia in Stata using the inverse variance method. Continuity correction of 0.5 has been applied for rare events. Figure B. Random effects meta-analysis for wound or scar problems in Stata using the inverse variance method. Continuity correction of 0.5 has been applied for rare events. Figure C. Random effects meta-analysis for recurrences in Stata using the inverse variance method. Continuity correction of 0.5 has been applied for rare events. Figure D. Random effects meta-analysis for reoperations in Stata using the inverse variance method. Continuity correction of 0.5 has been applied for rare events. Figure E. Random effects meta-analysis for major complications in Stata using the inverse variance method. Continuity correction of 0.5 has been applied for rare events. Figure F. Random effects meta-analysis for minor complications in Stata using the inverse variance method. Continuity correction of 0.5 has been applied for rare events. Figure G. Random effects meta-analysis for time to return to work in Stata using the inverse variance method. Figure H. Random effects meta-analysis for recurrences in Stata using the inverse variance method. Treatment arm correction has been applied for rare events. Figure I. Random effects meta-analysis for reoperations in Stata using the inverse variance method. Treatment arm correction has been applied for rare events. Figure J. Random effects meta-analysis for major complications in Stata using the inverse variance method. Treatment arm correction has been applied for rare events. Figure K. Random effects meta-analysis for minor complications in Stata using the inverse variance method. Treatment arm correction has been applied for rare events. Figure L. Contoured-enhanced funnel plot for the outcome recurrence. Figure M. Contoured-enhanced funnel plot for the outcome reoperation. Figure N. Contoured-enhanced funnel plot for the outcome major complications. Figure O. Contoured-enhanced funnel plot for the outcome minor complications. Figure P. Conditional power of an updated meta-analysis to detect an OR of 0.84 for the outcome of recurrence assuming one single hypothetical study is added. Figure Q. Conditional power of an updated meta-analysis to detect an OR of 0.74 for the outcome of reoperation assuming one single hypothetical study is added. Figure R. Conditional power of an updated meta-analysis to detect an OR of 0.99 for the outcome of major complications assuming one single hypothetical study is added. Figure S. Conditional power of an updated meta-analysis to detect an OR of 0.7 for the outcome of recurrence assuming one single hypothetical study is added. Figure T. Conditional power of an updated meta-analysis to detect an OR of 0.6 for the outcome of reoperation assuming one single hypothetical study is added. Figure U. Conditional power of an updated meta-analysis to detect an OR of 0.8 for the outcome of major complications assuming one single hypothetical study is added. Figure V. Power analysis of an updated meta-analysis based on simulations of new studies for the outcome of recurrence. Inference is based on statistical significance. The new study is simulated from the normal distribution. Figure W. Power analysis of an updated meta-analysis based on simulations of new studies for the outcome of reoperation. Inference is based on statistical significance. The new study is simulated from the normal distribution. Figure X. Power analysis of an updated meta-analysis based on simulations of new studies for the outcome of major complications. Inference is based on statistical significance. The new study is simulated from the normal distribution. Figure Y. Extended funnel plot for the outcome of recurrence (fixed effects). Figure Z. Extended funnel plot for the outcome of reoperation (fixed effects). Figure AA. Extended funnel plot for the outcome of major complications (fixed effects). Figure AB. Extended funnel plot for the outcome of minor complications (fixed effects). (PDF)

S3 File Figure A. Random effects meta-analysis for recurrence in Stata using the inverse variance method. Continuity correction of 0.5 has been applied for rare events. Studies with high risk of bias regarding allocation concealment have been excluded. Figure B. Random effects meta-analysis for reoperation in Stata using the inverse variance method. Continuity correction of 0.5 has been applied for rare events. Studies with high risk of bias regarding allocation concealment have been excluded. Figure C. Random effects meta-analysis for major complication in Stata using the inverse variance method. Continuity correction of 0.5 has been applied for rare events. Studies with high risk of bias regarding allocation concealment have been excluded. Figure D. Random effects meta-analysis for minor complication in Stata using the inverse variance method. Continuity correction of 0.5 has been applied for rare events. Studies with high risk of bias regarding allocation concealment have been excluded.
(PDF)

S1 Table The basic characteristics of the included studies and the detailed complications are reported by the authors. (PDF)

S2 Table (DOCX)
