# Supplementary material for: Endoscopic and Open Release Similarly Safe for the Treatment of Carpal Tunnel Syndrome. A Systematic Review and Meta-Analysis
Source: PLoS One. 2015 Dec 16;10(12):e0143683. doi: 10.1371/journal.pone.0143683 (PMC4682940; doi:10.1371/journal.pone.0143683)
Supplement: S1 Appendix — (DOCX) [file pone.0143683.s002.docx]

**1 Medline (OvidSP) search strategy**

Database: Ovid MEDLINE(R) <1946 to November Week 1 2013>

Search Strategy:

--------------------------------------------------------------------------------

1 randomized controlled trial.pt. (389866)

2 controlled clinical trial.pt. (89904)

3 randomized.ab. (287333)

4 placebo.ab. (156850)

5 drug therapy.fs. (1767223)

6 randomly.ab. (199448)

7 trial.ab. (302482)

8 groups.ab. (1276425)

9 or/1-8 (3299027)

10 exp animals/ not humans.sh. (4060470)

11 9 not 10 (2809295)

12 Carpal Tunnel Syndrome.mp. or Carpal Tunnel Syndrome/ (7915)

13 (carp$ tunn$ or tunn$ syndrom$ or carp$ syndrom$).mp. (9575)

14 (nerve entrapment or nerve compression or entrapment neuropath$).mp. (11216)

15 median nerve entrapment.mp. (99)

16 nerve compression syndromes/ (9072)

17 or/12-16 (19390)

18 endoscop$.mp. (159054)

19 OCTR.mp. (35)

20 ECTR.mp. (59)

21 releas$.mp. (600120)

22 or/18-21 (757515)

23 11 and 17 and 22 (328)

24 23 and 20121110:20131118.(ed). (21)

**2 Embase (OvidSP) search strategy**

Database: Embase <1980 to 2013 Week 46>

Search Strategy:

--------------------------------------------------------------------------------

1 crossover-procedure/ (38971)

2 double-blind procedure/ (118651)

3 randomized controlled trial/ (360008)

4 single-blind procedure/ (18506)

5 (random$ or factorial$ or crossover$ or cross over$ or cross-over$ or placebo$ or (doubl$ adj blind$) or (singl$ adj blind$) or assign$ or allocat$ or volunteer$).tw. (1303033)

6 or/1-5 (1385895)

7 exp animals/ (19025289)

8 exp humans/ (14995220)

9 7 not (7 and 8) (4030069)

10 6 not 9 (1245034)

11 limit 10 to embase (962420)

12 Carpal Tunnel Syndrome.mp. or Carpal Tunnel Syndrome/ (11573)

13 (carp$ tunn$ or tunn$ syndrom$ or carp$ syndrom$).mp. (14487)

14 (nerve entrapment or nerve compression or entrapment neuropath$).mp. (13134)

15 nerve compression/ (11098)

16 or/12-15 (25116)

17 carpal tunnel release/ (61)

18 (endoscop$ or releas$ or OCTR or ECTR).mp. (1055110)

19 or/17-18 (1055110)

20 11 and 16 and 19 (201)

21 20 and 20121015:20131118.(dd). (13)

**3 The Cochrane Library (CENTRAL)**

#1 "Carpal Tunnel Syndrome"
#2 ("nerve entrapment" or "nerve compression" or "entrapment neuropathy" or "entrapment neuropathies")
#3 carpal
#4 #1 or (#2 and #3)
#5 endoscop* or OCTR or ECTR or releas*
#6 #4 and #5

**4 search for ongoing trials**

Databases: *http://www.clinicaltrials.gov, http://www.controlled-trials.com* (ISRCTN Register, Action Medical Research (UK), NIH ClinicalTrials.gov, The Wellcome Trust (UK), UK trials (UK)) and http://www.who.int/ictrp/en/
