# Supplementary material for: Endoscopic and Open Release Similarly Safe for the Treatment of Carpal Tunnel Syndrome. A Systematic Review and Meta-Analysis
Source: PLoS One. 2015 Dec 16;10(12):e0143683. doi: 10.1371/journal.pone.0143683 (PMC4682940; doi:10.1371/journal.pone.0143683)
Supplement: S2 Appendix — (DOCX) [file pone.0143683.s003.docx]

## Definitions of Complications as assessed in the Review

## Minor Complications

- Recovery up to the latest follow up of the paper (except from Sudeck)
- Any scar problems (hypertrophy, tethering, tenderness)
- Any problem that was resolved at final follow up (eg pillar pain)
- Hypaesthesias, Paresthesias, Numbness when resolved by the latest follow up
- Scar pain characterised as “mild” was considered a complication only when reported at more than 2 years follow up
- With multiple time points; minor complications for pain or scar/palmar pain were measured at 3 months.

## Major Complications

- Sudeck
- Permanent nerve injury (eg Median nerve or digital nerves) (except from median cutaneous nerve)
- Moderate or severe pain at FU more than 1 year

## Recurrence

- “recurrence” as defined by the authors
- no improvement in symptoms at latest FU
- incomplete release

## Total Complications

- the summary of Minor Complications + Major Complications + Recurrences

We avoided double counting events as much as possible.

## OVERALL COMMENTS

- Whenever possible we counted number of hands with at least one minor or major complication.
- When minor, major or total complications were reported by the authors we recorded their number accepting the authors’ definition.
- In minor complications maybe not all categories were reported, though seem to be measured. In such cases, we reported it.
- When it was reported that no complications, or recurrences, or reoperations were observed or when we deducted from the text we put “0”. When nothing was reported we assumed the outcome was not measured.
- Some papers did not make clear whether recurrences were “0”or they were not reported. When calculated the total complications we added what available, so assuming that NR=0 for recurrence.
