# Supplementary material for: Endoscopic and Open Release Similarly Safe for the Treatment of Carpal Tunnel Syndrome. A Systematic Review and Meta-Analysis
Source: PLoS One. 2015 Dec 16;10(12):e0143683. doi: 10.1371/journal.pone.0143683 (PMC4682940; doi:10.1371/journal.pone.0143683)
Supplement: S3 Appendix — (PDF) [file pone.0143683.s004.pdf]

Appendix 3: Further statistical analysis

Details regarding the models used as well as supplementary statistical analyses and codes are presented below.

1. **Random-effects meta-analysis in Stata of recurrence, reoperation, minor complications and major complications using the 'reciprocal of the opposite arm size' continuity corrections**

Sweeting et al. proposed the 'reciprocal of the opposite arm size' method, according to which the continuity factors equal the reciprocal of the sample size in the opposite treatment arm (J. Sweeting et al., 2004). Meta-analysis results using the particular method are shown in Appendix Table 1 and Appendix 2 Figure 1: Random effects meta-analysis for transient neuropraxia in Stata using the inverse variance method. Continuity correction of 0.5 has been applied for rare events.

Appendix 2 Figure 2: Random effects meta-analysis for wound or scar problems in Stata using the inverse variance method. Continuity correction of 0.5 has been applied for rare events.

Appendix 2 Figure 3: Random effects meta-analysis for recurrences in Stata using the inverse variance method. Continuity correction of 0.5 has been applied for rare events.

Appendix 2 Figure 4: Random effects meta-analysis for reoperations in Stata using the inverse variance method. Continuity correction of 0.5 has been applied for rare events.

Appendix 2 Figure 5: Random effects meta-analysis for major complications in Stata using the inverse variance method. Continuity correction of 0.5 has been applied for rare events.

Appendix 2 Figure 6: Random effects meta-analysis for minor complications in Stata using the inverse variance method. Continuity correction of 0.5 has been applied for rare events.

Appendix 2 Figure 7: Random effects meta-analysis for time to return to work in Stata using the inverse variance method.

Appendix 2 Figure 8: Random effects meta-analysis for recurrences in Stata using the inverse variance method. Treatment arm correction has been applied for rare events.

Appendix 2 Figure 9: Random effects meta-analysis for reoperations in Stata using the inverse variance method. Treatment arm correction has been applied for rare events.

Appendix 2 Figure 10: Random effects meta-analysis for major complications in Stata using the inverse variance method. Treatment arm correction has been applied for rare events.

Appendix 2 Figure 11: Random effects meta-analysis for minor complications in Stata using the inverse variance method. Treatment arm correction has been applied for rare events.

Appendix 2 Figure 12: Contoured-enhanced funnel plot for the outcome recurrence.

Appendix 2 Figure 13: Contoured-enhanced funnel plot for the outcome reoperation.

Appendix 2 Figure 14: Contoured-enhanced funnel plot for the outcome major complications.

Appendix 2 Figure 15: Contoured-enhanced funnel plot for the outcome minor complications.

Appendix 2 Figure 16: Conditional power of an updated meta-analysis to detect an OR of 0.84 for the outcome of recurrence assuming one single hypothetical study is added

Appendix 2 Figure 17: Conditional power of an updated meta-analysis to detect an OR of 0.74 for the outcome of reoperation assuming one single hypothetical study is added

Appendix 2 Figure 18: Conditional power of an updated meta-analysis to detect an OR of 0.99 for the outcome of major complications assuming one single hypothetical study is added

Appendix 2 Figure 19: Conditional power of an updated meta-analysis to detect an OR of 0.7 for the outcome of recurrence assuming one single hypothetical study is added

Appendix 2 Figure 20: Conditional power of an updated meta-analysis to detect an OR of 0.6 for the outcome of reoperation assuming one single hypothetical study is added

Appendix 2 Figure 21: Conditional power of an updated meta-analysis to detect an OR of 0.8 for the outcome of major complications assuming one single hypothetical study is added

Appendix 2 Figure 22: Power analysis of an updated meta-analysis based on simulations of new studies for the outcome of recurrence. Inference is based on statistical significance. The new study is simulated from the normal distribution.

Appendix 2 Figure 23: Power analysis of an updated meta-analysis based on simulations of new studies for the outcome of reoperation. Inference is based on statistical significance. The new study is simulated from the normal distribution.

Appendix 2 Figure 24: Power analysis of an updated meta-analysis based on simulations of new studies for the outcome of major complications. Inference is based on statistical significance. The new study is simulated from the normal distribution.

Appendix 2 Figure 25: Extended funnel plot for the outcome of recurrence (fixed effects)

Appendix 2 Figure 26: Extended funnel plot for the outcome of reoperation (fixed effects)

Appendix 2 Figure 27: Extended funnel plot for the outcome of major complications (fixed effects)

Appendix 2 Figure 28: Extended funnel plot for the outcome of minor complications (fixed effects)

. Zero trials (trials with no events in both groups) were excluded. Meta-analyses were performed in Stata. Appendix Table 1 implies that ECTR has a lower incidence of minor complications than OCTR whereas no significant difference has been detected in terms of the rest three outcomes. Results were consistent to the main analysis using

the conventional continuity correction of 0.5. All predictive intervals indicate that neither intervention is more effective in all considered settings; a future study may suggest greater safety with ECTR, OCTR or no difference at all. The predictive interval is calculated using the formula $t_{k-2}\sqrt{se^2 + \tau^2}$ where t_{k-2} is the t distribution with $k - 2$ degrees of freedom, se is the standard error and τ^2 is the heterogeneity (Higgins et al., 2009). When heterogeneity is zero the predictive interval is slightly larger than the confidence interval as the t-statistic is always greater than the corresponding normal deviate. All forest plots are ordered chronologically so that any time trend is depictable; no evidence of a trend of the effect estimates over years was observed.

2. Random-effects Bayesian meta-analysis of the outcomes recurrence, reoperation, minor complications and major complications

We performed Bayesian meta-analysis using WinBUGS for the outcomes including zero trials (Appendix Table 1). We used empirical evidence to specify a plausible prior distribution for the heterogeneity parameter (Turner et al., 2012). For a semi-objective and a 'non-pharmacological versus any' intervention comparison type a log-normal distribution is suggested for heterogeneity, $\tau \sim LN(-2.89, 1.91)$ (Turner et al., 2012). Reoperation is associated with larger uncertainty compared to the analysis performed in Stata. According to the rest of comparisons, both point estimates and confidence intervals are similar to those obtained in Stata.

3. Bivariate Bayesian meta-analysis of the outcomes recurrence, reoperation minor complications and major complications

Four trials included only patients with bilateral CTS whereas in 10 studies some but not all of the patients had bilateral CTS (see Included studies). We performed a bivariate Bayesian model in order to take into account the correlation in the outcomes. Let the binomial distributions

$$r_{iE} \sim Bin(p_{iE}, n_{iE})$$
$$r_{iO} \sim Bin(p_{iO}, n_{iO})$$

for the number of events in the ith study in the ECTR and OCTR groups respectively. The quantities p_{iE} and p_{iO} denote the probabilities of an event and n_{iE} and n_{iO} are the sample sizes in the two groups. The log-odds for the OCTR group in the ith study is denoted by u_i and the log(OR) is θ_i. Then the model is parameterized as

$$logit(p_{iO}) = u_i$$
$$logit(p_{iE}) = u_i + \theta_i$$

where u_i and $(u_i + \theta_i)$ follow a bivariate normal distribution. In particular,

$$u_i \sim N(0, 0.001)$$
$$(u_i + \theta_i)|u_i \sim N(mth_i, vth_i)$$

with

$mth_i = m + r_i\sqrt{\frac{s_u}{s_\theta}}(u_i)$ where $m \sim N(0, 0.01)$, $s_u = 1/0.01$, $s_\theta = \tau^2$ and r_i denotes the correlation coefficient. The variance vth_i equals $(1 - r_i^2)s_\theta$. We use $r_i = \frac{hands_i - patients_i}{patients_i}$ as study specific correlation coefficient. This measure is bounded $(0 \leq r_i \leq 1)$ and equals 0 if no bilateral exists in a trial and 1 if only patients with bilateral CTS are included. For those studies that we know that bilateral CTS exists but we do not know its extent we impute a correlation coefficient of 0.5 (Appendix Table 2).

The results of the bivariate Bayesian meta-analysis assuming a study specific correlation coefficient are shown in Appendix Table 3. Zero trials were not excluded from the analysis and informative prior has been used for heterogeneity $\left(\tau^2 \sim LN(-2.89, 1.91^2)\right)$. Results were consistent to those obtained in Stata using both continuity corrections and to those obtained from the univariate Bayesian meta-analysis performed in WinBUGS. Inference about relative safety between interventions does not change with the consideration of the correlation introduced by bilateral CTS.

4. Poisson meta-analysis for safety outcomes

We consider the variable of total complications as the sum of the events in the outcomes recurrence, reoperations, minor complications and major complications, rt. We assume that the total complications are drawn from a Poisson distribution

$$rt_{Oi} \sim Poi(\lambda_{Oi} \cdot L_{Oi})$$
$$rt_{Ei} \sim Poi(\lambda_{Ei} \cdot L_{Ei})$$

where λ_{Oi} and λ_{Ei} are the rates of events per month in the ith study for the OCTR and the ECTR groups respectively. The quantities L_{Oi} and L_{Ei} are the products of the study's sample size and its duration in months for the two groups. The log-rate for the

OCTR group in the ith study is denoted by u_i and the log rate ratio of ECTR versus OCTR is δ_i. Then the model is parameterized as

$$log(\lambda_{Oi}) = u_i$$

$$log(\lambda_{Ei}) = u_i + \delta_i$$

where

$$u_i \sim N(0,0.0001)$$

and

$$\delta_i \sim N(m, \tau^2)$$

with

$$m \sim N(0, 0.01)$$

5. **Univariate and bivariate Bayesian meta-analysis for time to return to work**

We initially analyzed the outcome time to return to work in WinBUGS without taking into consideration the bilateral effect and then we performed a bivariate Bayesian meta-analysis model. We assume that studies have different correlations according to the extent of bilateral CTS as described in section 3. Appendix Table 1 shows the results from the univariate and the bivariate meta-analyses for the time to return to work outcome. A half normal distribution is used $\left(\tau \sim N(0,0.01)I(0,)\right)$ for the heterogeneity variance. Appendix Table 1 implies that ECTR is significantly better than OCTR in terms of time to return to work for both assumptions regarding the correlation.

6. **Small study effects**

We evaluated small study effects via contour-enhanced funnel plots (**Error! Reference source not found.**). There is an evidence of small study effects for the outcome minor complications suggesting that small studies tend to favor OCTR.

7. **Is the current evidence convincing and conclusive?**

<u>**Conditional power of a meta-analysis**</u>

Given the fact that the outcomes recurrence, reoperation, major complications and minor complications are rare, the existing evidence might not be adequate to conclude which intervention is safer. We investigated whether meta-analysis results would change if further evidence is accumulated. We calculated the conditional power of an updated meta-analysis for each non-significant outcome after the addition of a hypothetical new study. The alternative ORs are assumed to be either equal to the observed effect sizes or effect sizes indicating a relative benefit of ECTR versus OCTR in terms of complications. The reason underlying the choice of the alternative effect sizes lies on our aim to investigate whether there is a need for further research under the assumption that new studies will show a better performance of ECTR compared to OCTR in terms of complications. **Error! Reference source not found.** suggest that the power to detect the pre-specified alternative effects remains lower than 50% for the outcomes recurrence, reoperation and major complications even with an inclusion of 5000 patients.

Roloff's method of conditional power requires two parameters of heterogeneity, one estimated from the existing meta-analysis (old) and one assumed for the future data (new). We used the DerSimonian and Laird estimate to specify old estimate of heterogeneity and we used empirical evidence to specify the value of the new heterogeneity (Turner et al., 2012). The continuity correction used to handle studies with 0 events in one of the two groups is the treatment arm correction.

Power of an updated meta-analysis based on simulations of new studies

Sutton et al. developed a method to estimate the power of an updated meta-analysis based on simulations of new studies (Sutton et al., 2007). Several criteria on which to base inference and consequently power could be considered. The conventional approach is based on the statistical significance of the pooled estimate. The power is calculated as the proportion of times that the meta-analysis result is significant. The power curves in **Error! Reference source not found.** show the power of the updated meta-analysis for the outcomes recurrence, reoperation and major complications considering a new study with sample size specified by the horizontal axis. A random effects model is assumed and the heterogeneity is estimated with the DerSimonian-Laird method. The continuity correction used to handle studies with 0 events in one of the two groups is the treatment arm correction. The new study is simulated from the normal distribution. The number of simulations on which the estimated power is based is 1000. The power that an updated meta-analysis would change conclusions in

terms of statistical significance after the inclusion of a new study is very low (**Error! Reference source not found.**.

Extended funnel plots

Langan et al. developed a method to investigate the impact of including new studies in a meta-analysis appending several augmentations to the funnel plot (Langan et al., 2012). The extended funnel plots in **Error! Reference source not found.** show the potential conclusions of an updated meta-analysis if the findings of a new trial were to be added to the existing meta-analysis. As heterogeneity is small, we assume a fixed-effect meta-analysis.

The extended funnel plots in **Error! Reference source not found.** indicate that it is not likely that a new trial could show a statistical significant result for the comparison 'ECTR versus OCTR' in terms of the outcomes recurrence, reoperation and major complications because the shaded areas are relative small. However, studies with very small standard error (smaller than 1) could change conclusions to a significant effect in either direction. As none of the observed studies is located in the shaded areas it is not considered likely that a new trial will change meta-analysis conclusions.

The color code in **Error! Reference source not found.** indicates that it is very likely that the inclusion of a new study will lead to a conclusion that ECTR is superior compared to OCTR in terms of minor complications. An additional study that would lie in the small non-shaded right hand region of **Error! Reference source not found.** would demonstrate an OR in the opposite direction of the current meta-analysis and thus the updated meta-analysis would obtain a non-significant result. Thus, the only case that the updated meta-analysis could change inferences about the best technique in terms of minor complications is the conduct of a big new study that would show an extreme result in the opposite direction from this of the current meta-analysis; as none of the existing studies lies in the non-shaded region we consider that it unlikely for the meta-analysis to change conclusions with the addition of new studies.

8. Winbugs codes

We present the Winbugs codes for the sensitivity analyses we performed regarding the outcomes recurrence, reoperation, major complications and minor complications.

8.1 Winbugs code for the Bayesian meta-analysis assuming a binomial distribution

```
#ns=number of studies
#r.E and r.O=number of events in treatment E and O
#n.E and n.O=sample size in treatment E and O
#tau.sq=heterogeneity
```

```
model {
	for (i in 1:ns) {

##binomial likelihood of number of events for each arm of study i
		r.O[i] ~ dbin(p.O[i],n.O[i])
	  r.E[i] ~ dbin(p.E[i],n.E[i])

##parameterization of the 'true' effect of each study i
		logit(p.O[i]) <- u[i]
		logit(p.E[i]) <- u[i]+theta[i]

##distribution of random effects
		theta[i] ~ dnorm(mean,prec)
		u[i] ~ dnorm(0,.0001)}

##prior distribution for summary lnOR
mean ~ dnorm(0,.01)

##prior distribution for log-odds in baseline arm of study i
	#for (i in 1:ns) {u[i] ~ dnorm(0,.0001)}

##prior distribution for heterogeneity semi-objective non-pharmacological
	tausq ~ dlnorm(-2.89,0.27) #log-normal prior with mean -2.89, precision 1/(1.91)^2
	prec <- 1/tausq
	tau <- pow(tausq,0.5)

##OR
		OR <- exp(mean)
		LOR <- mean
		theta.new ~ dnorm(mean,prec)
		OR.new <- exp(theta.new)

	}
   }
```

8.2 Winbugs code for the Bayesian bivariate model

```
#ns=number of studies
#r.E and r.O=number of events in treatment E and O
#n.E and n.O=sample size in treatment E and O
#tau.sq=heterogeneity

model {
	for (i in 1:ns) {

##binomial likelihood of number of events for each arm of study i
		r.O[i] ~ dbin(p.O[i],n.O[i])
	  r.E[i] ~ dbin(p.E[i],n.E[i])

##parameterization of the 'true' effect of each study i
		logit(p.O[i]) <- u[i]
		logit(p.E[i]) <- u[i]+theta[i]

##distribution of random effects
		theta[i] ~ dnorm(mean,prec)
		u[i] ~ dnorm(0,.0001)}

##prior distribution for summary lnOR
```

```
mean ~ dnorm(0,.01)

##prior distribution for log-odds in baseline arm of study i
        #for (i in 1:ns) {u[i] ~ dnorm(0,.0001)}

##prior distribution for heterogeneity semi-objective non-pharmacological
        tau ~ dlnorm(-2.89,0.27) #log-normal prior with mean -2.89, precision 1/(1.91)^2
        #tau ~ dunif(0,100)
        prec <- 1/tausq
        tausq <- pow(tau,2)


##OR
                OR <- exp(mean)
                LOR <- mean
                theta.new ~ dnorm(mean,prec)

        }
    }
```

8.3 Winbugs code for the Poisson model regarding the outcome total complications

```
#ns=number of studies
#r.E and r.O=number of events in treatment E and O
#n.E and n.O=sample size in treatment E and O
#tau.sq=heterogeneity

model {
        for (i in 1:ns) {
        theta.O[i] <- lamda.O[i]*L.O[i] #rate*exposure
        theta.E[i] <- lamda.E[i]*L.E[i]

##poisson likelihood of number of events for each arm of study i
                r.O[i] ~ dpois(theta.O[i])
         r.E[i] ~ dpois(theta.E[i])

##parameterization of the 'true' effect of each study i
                log(lamda.O[i]) <- u[i]
                log(lamda.E[i]) <- u[i]+delta[i]

##distribution of random effects
                delta[i] ~ dnorm(mean,prec)
                u[i] ~ dnorm(0,.0001)}

##prior distribution for summary lnOR
mean ~ dnorm(0,.01)

##prior distribution for log-odds in baseline arm of study i
        #for (i in 1:ns) {u[i] ~ dnorm(0,.0001)}

##prior distribution for heterogeneity semi-objective non-pharmacological
        #tau ~ dlnorm(-2.89,0.27) #log-normal prior with mean -2.89, precision 1/(1.91)^2
        tau ~ dnorm(0,1)I(0,)
        #tau ~ dunif(0,100)
        prec <- 1/tausq
        tausq <- pow(tau,2)
```

```
##RateR
			RateR <- exp(mean)
			LRateR <- mean
			theta.new ~ dnorm(mean,prec)

		}
}
```

Appendix Table 1: Sensitivity analysis for the outcomes recurrence, reoperations, major complications and minor complications. Summary estimates are derived from meta-analysis using random effects model. OR greater than 1 favors OCTR.

Method of analysis	OR (95% CI)	Predictive interval	τ
**Recurrence**			
'Reciprocal of the opposite arm size' continuity correction	0.84 (0.41, 1.71)	(0.37, 1.71)	0
Bayesian meta-analysis using the binomial distribution	1.18 (0.57, 2.49)	(0.39, 4.11)	0.23
Bivariate Bayesian meta-analysis	1.15 (0.57, 2.50)	(0.34, 4.41)	0.25
**Reoperation**			
'Reciprocal of the opposite arm size' continuity correction	0.74 (0.23, 2.37)	(0.19, 2.83)	0
Bayesian meta-analysis using the binomial distribution	2.11 (0.85, 10.01)	(0.37, 34.24)	0.39
Bivariate Bayesian meta-analysis	2.06 (0.75, 6.00)	(0.41, 14.49)	2.00
**Major complications**			
'Reciprocal of the opposite arm size' continuity correction	0.99 (0.32, 3.02)	(0.26, 3.68)	0
Bayesian meta-analysis using the binomial distribution	0.93 (0.36, 2.45)	(0.21, 4.12)	0.26
Bivariate Bayesian meta-analysis	0.87 (0.37, 2.20)	(0.21, 3.45)	0.25
**Minor complications**			
'Reciprocal of the opposite arm size' continuity correction	0.46 (0.29, 0.73)	(0.14, 1.52)	0.52
Bayesian meta-analysis using the binomial distribution	0.47 (0.29, 0.83)	(0.11, 2.42)	0.61
Bivariate Bayesian meta-analysis	0.47 (0.29, 0.82)	(0.09, 2.86)	0.68

Appendix Table 2. Number of hands, number of patients and correlation coefficients for these studies that part of the patients had bilateral CTS.

Study	Hands	Patients	Correlation coefficient
Agee et al., 1992	147	122	0.20
Aslani et al., 2012	Non-reported	Non-reported	0.50
Brown et al., 1993	169	151	0.12
Ejiri et al., 2012	Non-reported	Non-reported	0.50
Erdmann, 1994	105	71	0.48
Jacobsen and Rahme, 1996	32	29	0.10
Koskella K and Alexander, 1996	17	16	0.06
Tian et al., 2007	70	62	0.13
Trumble et al., 2002	192	147	0.31
Tuzuner et al., 2008	16	13	0.23

Appendix Table 3. Summary estimates for the time to return to work outcome derived from univariate and bivariate Bayesian meta-analysis using random effects model. MD greater than 0 favors OCTR.

Time to return to work				
**MD (95% CI)**	**Predictive interval**	**Number of studies**	**τ**	**Correlation coefficient**
-9.51 (-13.08, -5.85)	(-22.01, 3.06)	13	5.51	0
-10.13 (-13.65, -6.88)	(-23.28, 2.64)	13	5.76	Study specific

Figure legends:

Appendix 2 Figure 1: Random effects meta-analysis for transient neuropraxia in Stata using the inverse variance method. Continuity correction of 0.5 has been applied for rare events.

Appendix 2 Figure 2: Random effects meta-analysis for wound or scar problems in Stata using the inverse variance method. Continuity correction of 0.5 has been applied for rare events.

Appendix 2 Figure 3: Random effects meta-analysis for recurrences in Stata using the inverse variance method. Continuity correction of 0.5 has been applied for rare events.

Appendix 2 Figure 4: Random effects meta-analysis for reoperations in Stata using the inverse variance method. Continuity correction of 0.5 has been applied for rare events.

Appendix 2 Figure 5: Random effects meta-analysis for major complications in Stata using the inverse variance method. Continuity correction of 0.5 has been applied for rare events.

Appendix 2 Figure 6: Random effects meta-analysis for minor complications in Stata using the inverse variance method. Continuity correction of 0.5 has been applied for rare events.

Appendix 2 Figure 7: Random effects meta-analysis for time to return to work in Stata using the inverse variance method.

Appendix 2 Figure 8: Random effects meta-analysis for recurrences in Stata using the inverse variance method. Treatment arm correction has been applied for rare events.

Appendix 2 Figure 9: Random effects meta-analysis for reoperations in Stata using the inverse variance method. Treatment arm correction has been applied for rare events.

Appendix 2 Figure 10: Random effects meta-analysis for major complications in Stata using the inverse variance method. Treatment arm correction has been applied for rare events.

Appendix 2 Figure 11: Random effects meta-analysis for minor complications in Stata using the inverse variance method. Treatment arm correction has been applied for rare events.

Appendix 2 Figure 12: Contoured-enhanced funnel plot for the outcome recurrence.

Appendix 2 Figure 13: Contoured-enhanced funnel plot for the outcome reoperation.

Appendix 2 Figure 14: Contoured-enhanced funnel plot for the outcome major complications.

Appendix 2 Figure 15: Contoured-enhanced funnel plot for the outcome minor complications.

Appendix 2 Figure 16: Conditional power of an updated meta-analysis to detect an OR of 0.84 for the outcome of recurrence assuming one single hypothetical study is added

Appendix 2 Figure 17: Conditional power of an updated meta-analysis to detect an OR of 0.74 for the outcome of reoperation assuming one single hypothetical study is added

Appendix 2 Figure 18: Conditional power of an updated meta-analysis to detect an OR of 0.99 for the outcome of major complications assuming one single hypothetical study is added

Appendix 2 Figure 19: Conditional power of an updated meta-analysis to detect an OR of 0.7 for the outcome of recurrence assuming one single hypothetical study is added

Appendix 2 Figure 20: Conditional power of an updated meta-analysis to detect an OR of 0.6 for the outcome of reoperation assuming one single hypothetical study is added

Appendix 2 Figure 21: Conditional power of an updated meta-analysis to detect an OR of 0.8 for the outcome of major complications assuming one single hypothetical study is added

Appendix 2 Figure 22: Power analysis of an updated meta-analysis based on simulations of new studies for the outcome of recurrence. Inference is based on statistical significance. The new study is simulated from the normal distribution.

Appendix 2 Figure 23: Power analysis of an updated meta-analysis based on simulations of new studies for the outcome of reoperation. Inference is based on statistical significance. The new study is simulated from the normal distribution.

Appendix 2 Figure 24: Power analysis of an updated meta-analysis based on simulations of new studies for the outcome of major complications. Inference is based on statistical significance. The new study is simulated from the normal distribution.

Appendix 2 Figure 25: Extended funnel plot for the outcome of recurrence (fixed effects)

Appendix 2 Figure 26: Extended funnel plot for the outcome of reoperation (fixed effects)

Appendix 2 Figure 27: Extended funnel plot for the outcome of major complications (fixed effects)

Appendix 2 Figure 28: Extended funnel plot for the outcome of minor complications (fixed effects)

References

Higgins, J.P.T., Thompson, S.G., and Spiegelhalter, D.J. (2009). A re-evaluation of random-effects meta-analysis. J. R. Stat. Soc. Ser. A Stat. Soc. *172*, 137–159.

J. Sweeting, M., J. Sutton, A., and C. Lambert, P. (2004). What to add to nothing? Use and avoidance of continuity corrections in meta-analysis of sparse data. Stat. Med. *23*, 1351–1375.

Langan, D., Higgins, J.P.T., Gregory, W., and Sutton, A.J. (2012). Graphical augmentations to the funnel plot assess the impact of additional evidence on a meta-analysis. J. Clin. Epidemiol. *65*, 511–519.

Sutton, A.J., Cooper, N.J., Jones, D.R., Lambert, P.C., Thompson, J.R., and Abrams, K.R. (2007). Evidence-based sample size calculations based upon updated meta-analysis. Stat. Med. *26*, 2479–2500.

Turner, R.M., Davey, J., Clarke, M.J., Thompson, S.G., and Higgins, J.P. (2012). Predicting the extent of heterogeneity in meta-analysis, using empirical data from the Cochrane Database of Systematic Reviews. Int. J. Epidemiol. *41*, 818–827.
