# Supplementary material for: Endoscopic and Open Release Similarly Safe for the Treatment of Carpal Tunnel Syndrome. A Systematic Review and Meta-Analysis
Source: PLoS One. 2015 Dec 16;10(12):e0143683. doi: 10.1371/journal.pone.0143683 (PMC4682940; doi:10.1371/journal.pone.0143683)
Supplement: S1 File — (PDF) [file pone.0143683.s005.pdf]

Figure A

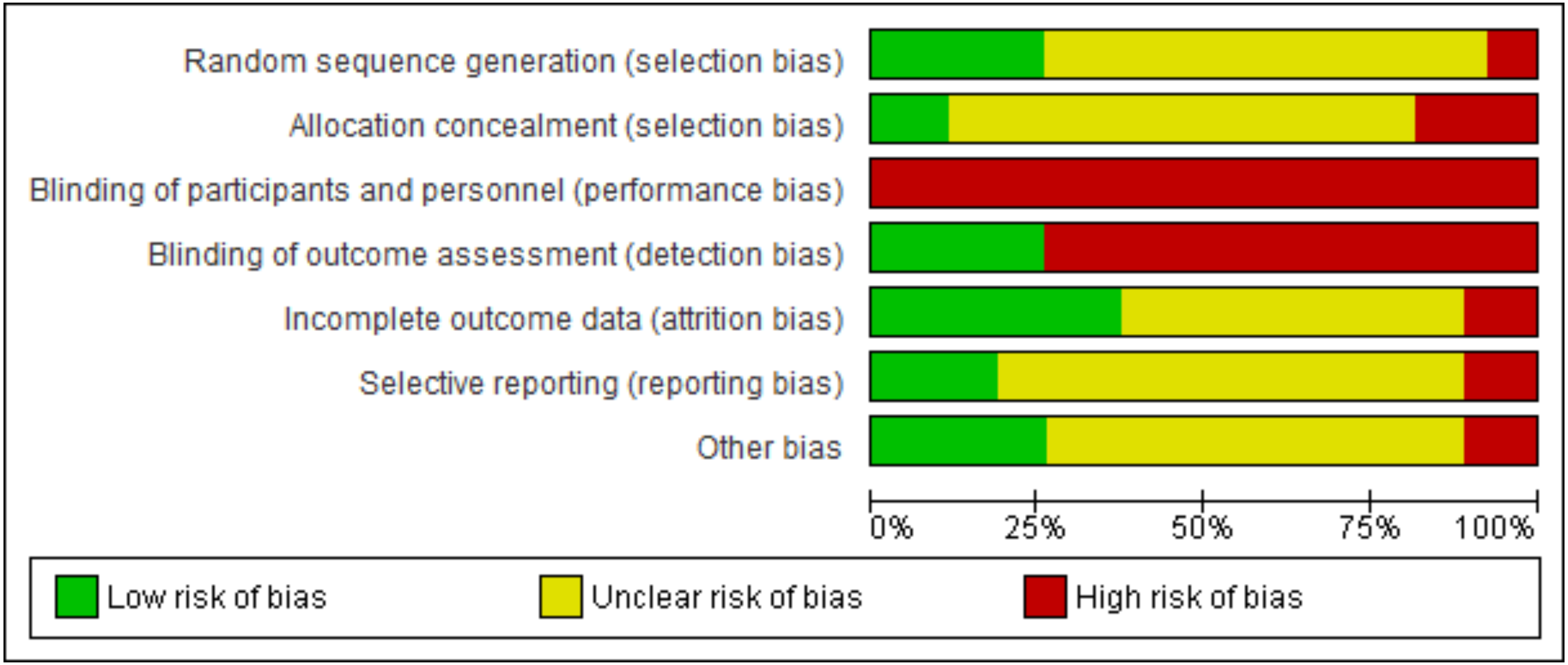
Random sequence generation (selection bias)
Allocation concealment (selection bias)
Blinding of participants and personnel (performance bias)
Blinding of outcome assessment (detection bias)
Incomplete outcome data (attrition bias)
Selective reporting (reporting bias)
Other bias
0%
25%
50%
75%
100%
Low risk of bias
Unclear risk of bias
High risk of bias

Figure B

	Westphal 2000	Werber 1996	Tuzuner 2008	Trumble 2002	Tian 2007	Stark 1996	Schaefer 1996	Saw 2003	Malhotra 2007	Macdermid 2003	Larsen 2013	Koskella 1996	Jacobsen 1996	Incoll 2004	Hoefnagels 1997	Giele 2000	Foucher 1993	Ferdinand 2002	Erdmann 1994	Ejiri 2012	Eighhorn 2003	Dumontier 1995	Brown 1993	Benedetti/Sennwald 1995	Atroshi 2006	Aslani 2012	Agee 1992
Random sequence generation (selection bias)	?	?	+	?	?	?	−	+	?	?	+	?	?	?	?	?	?	+	?	+	?	?	−	+	+	?	?
Allocation concealment (selection bias)	?	?	+	−	?	?	−	?	?	?	+	?	?	?	?	?	?	?	?	?	?	−	−	?	+	?	−
Blinding of participants and personnel (performance bias)	−	−	−	−	−	−	−	−	−	−	−	−	−	−	−	−	−	−	−	−	−	−	−	−	−	−	−
Blinding of outcome assessment (detection bias)	−	−	−	+	−	−	−	+	−	+	+	−	−	+	−	−	−	+	−	−	−	−	+	−	−	−	−
Incomplete outcome data (attrition bias)	?	?	+	?	?	+	+	+	?	?	+	?	+	?	+	?	?	?	?	+	−	−	?	+	+	−	?
Selective reporting (reporting bias)	?	?	+	−	?	−	?	?	?	?	+	?	?	?	+	?	?	?	?	+	?	?	−	?	+	?	?
Other bias	?	?	+	+	?	?	?	−	+	?	+	?	?	?	+	?	?	?	−	?	?	?	+	?	+	?	−
