# Supplementary material for: Endoscopic and Open Release Similarly Safe for the Treatment of Carpal Tunnel Syndrome. A Systematic Review and Meta-Analysis
Source: PLoS One. 2015 Dec 16;10(12):e0143683. doi: 10.1371/journal.pone.0143683 (PMC4682940; doi:10.1371/journal.pone.0143683)
Supplement: S2 File — (PDF) [file pone.0143683.s006.pdf]

Figure A

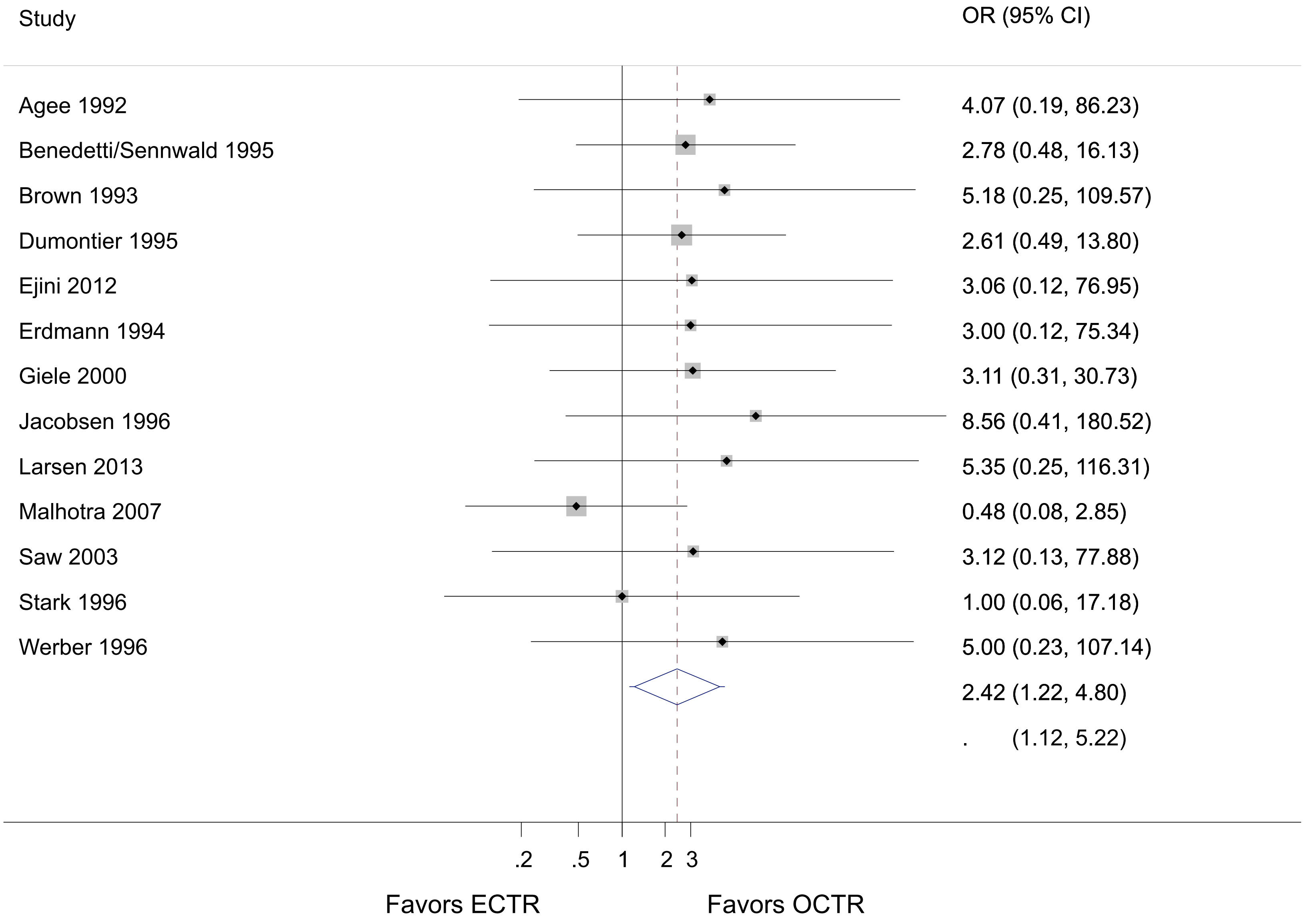

Study
OR (95% CI)
Agee 1992
4.07 (0.19, 86.23)
Benedetti/Sennwald 1995
2.78 (0.48, 16.13)
Brown 1993
5.18 (0.25, 109.57)
Dumontier 1995
2.61 (0.49, 13.80)
Ejini 2012
3.06 (0.12, 76.95)
Erdmann 1994
3.00 (0.12, 75.34)
Giele 2000
3.11 (0.31, 30.73)
Jacobsen 1996
8.56 (0.41, 180.52)
Larsen 2013
5.35 (0.25, 116.31)
Malhotra 2007
0.48 (0.08, 2.85)
Saw 2003
3.12 (0.13, 77.88)
Stark 1996
1.00 (0.06, 17.18)
Werber 1996
5.00 (0.23, 107.14)
2.42 (1.22, 4.80)
. (1.12, 5.22)
.2
.5
1
2
3
Favors ECTR
Favors OCTR

Figure B

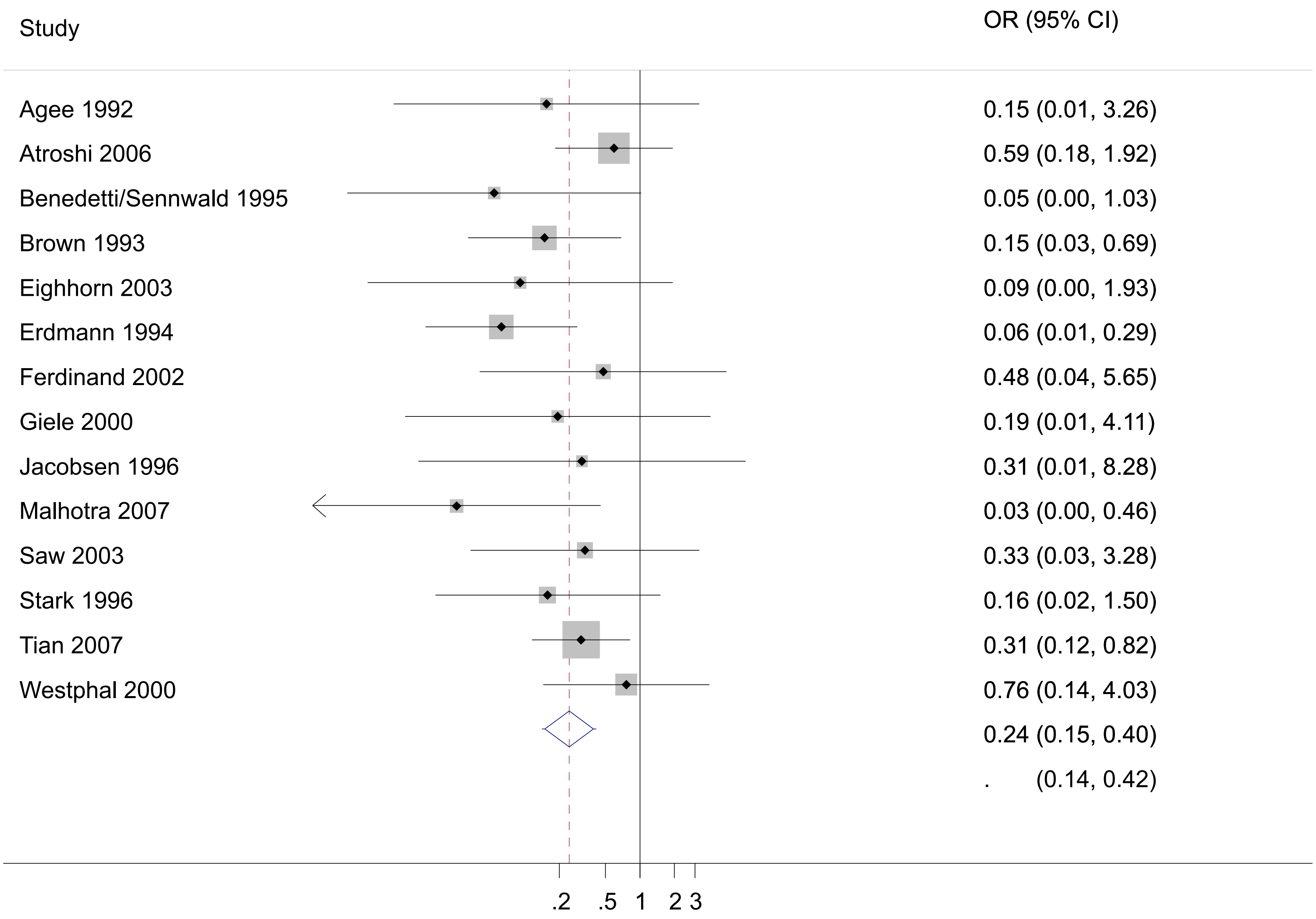
Study
OR (95% CI)
Agee 1992
0.15 (0.01, 3.26)
Atroshi 2006
0.59 (0.18, 1.92)
Benedetti/Sennwald 1995
0.05 (0.00, 1.03)
Brown 1993
0.15 (0.03, 0.69)
Eighhorn 2003
0.09 (0.00, 1.93)
Erdmann 1994
0.06 (0.01, 0.29)
Ferdinand 2002
0.48 (0.04, 5.65)
Giele 2000
0.19 (0.01, 4.11)
Jacobsen 1996
0.31 (0.01, 8.28)
Malhotra 2007
0.03 (0.00, 0.46)
Saw 2003
0.33 (0.03, 3.28)
Stark 1996
0.16 (0.02, 1.50)
Tian 2007
0.31 (0.12, 0.82)
Westphal 2000
0.76 (0.14, 4.03)
0.24 (0.15, 0.40)
. (0.14, 0.42)
.2
.5
1
2
3

Figure C

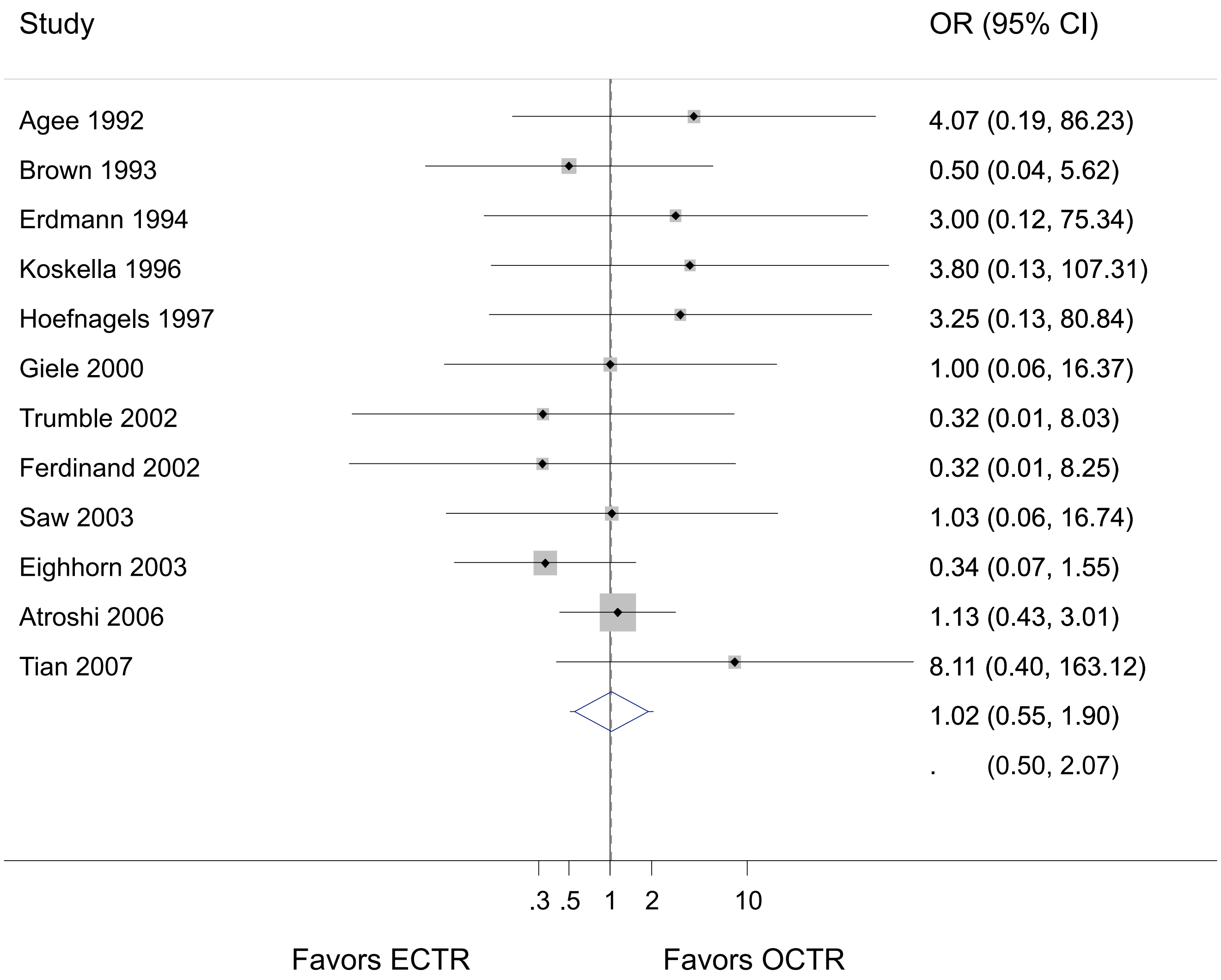
Study
OR (95% CI)
Agee 1992
4.07 (0.19, 86.23)
Brown 1993
0.50 (0.04, 5.62)
Erdmann 1994
3.00 (0.12, 75.34)
Koskella 1996
3.80 (0.13, 107.31)
Hoefnagels 1997
3.25 (0.13, 80.84)
Giele 2000
1.00 (0.06, 16.37)
Trumble 2002
0.32 (0.01, 8.03)
Ferdinand 2002
0.32 (0.01, 8.25)
Saw 2003
1.03 (0.06, 16.74)
Eighhorn 2003
0.34 (0.07, 1.55)
Atroshi 2006
1.13 (0.43, 3.01)
Tian 2007
8.11 (0.40, 163.12)
1.02 (0.55, 1.90)
. (0.50, 2.07)
.3
.5
1
2
10
Favors ECTR
Favors OCTR

Figure D

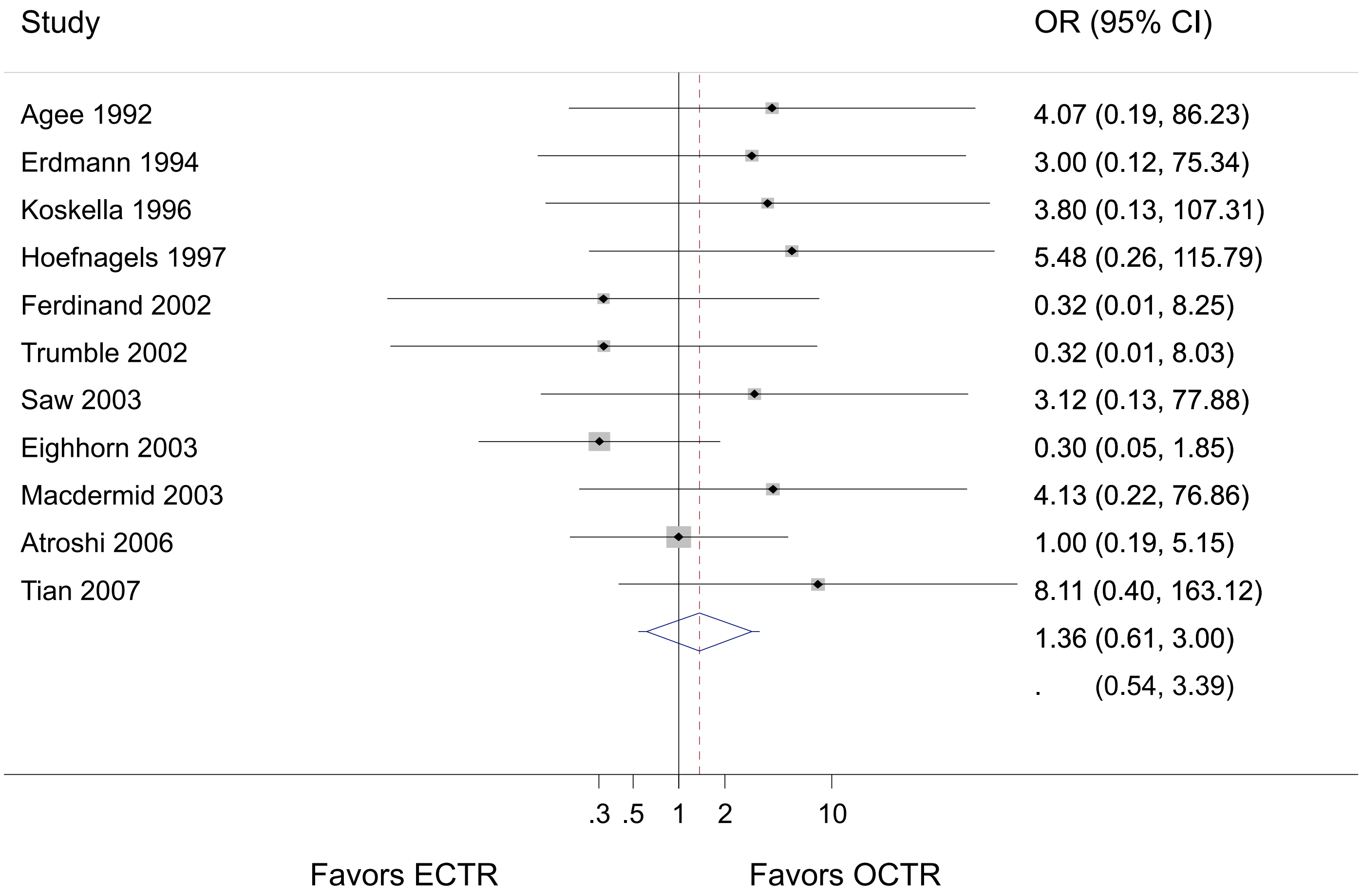
Study
OR (95% CI)
Agee 1992
4.07 (0.19, 86.23)
Erdmann 1994
3.00 (0.12, 75.34)
Koskella 1996
3.80 (0.13, 107.31)
Hoefnagels 1997
5.48 (0.26, 115.79)
Ferdinand 2002
0.32 (0.01, 8.25)
Trumble 2002
0.32 (0.01, 8.03)
Saw 2003
3.12 (0.13, 77.88)
Eighhorn 2003
0.30 (0.05, 1.85)
Macdermid 2003
4.13 (0.22, 76.86)
Atroshi 2006
1.00 (0.19, 5.15)
Tian 2007
8.11 (0.40, 163.12)
1.36 (0.61, 3.00)
. (0.54, 3.39)
.3
.5
1
2
10
Favors ECTR
Favors OCTR

Figure E

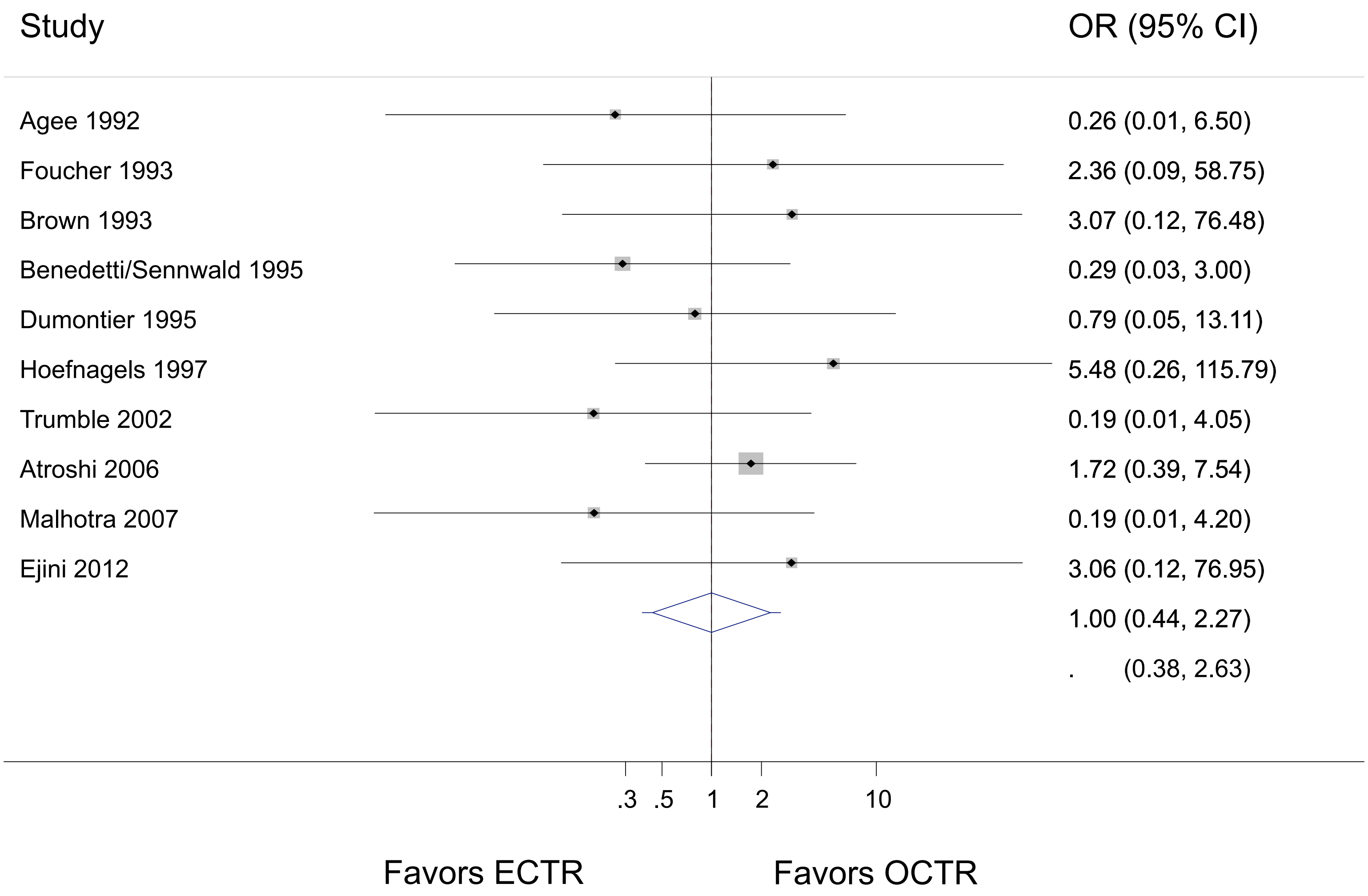

Study
OR (95% CI)
Agee 1992
0.26 (0.01, 6.50)
Foucher 1993
2.36 (0.09, 58.75)
Brown 1993
3.07 (0.12, 76.48)
Benedetti/Sennwald 1995
0.29 (0.03, 3.00)
Dumontier 1995
0.79 (0.05, 13.11)
Hoefnagels 1997
5.48 (0.26, 115.79)
Trumble 2002
0.19 (0.01, 4.05)
Atroshi 2006
1.72 (0.39, 7.54)
Malhotra 2007
0.19 (0.01, 4.20)
Ejini 2012
3.06 (0.12, 76.95)
1.00 (0.44, 2.27)
. (0.38, 2.63)
.3
.5
1
2
10
Favors ECTR
Favors OCTR

Figure F

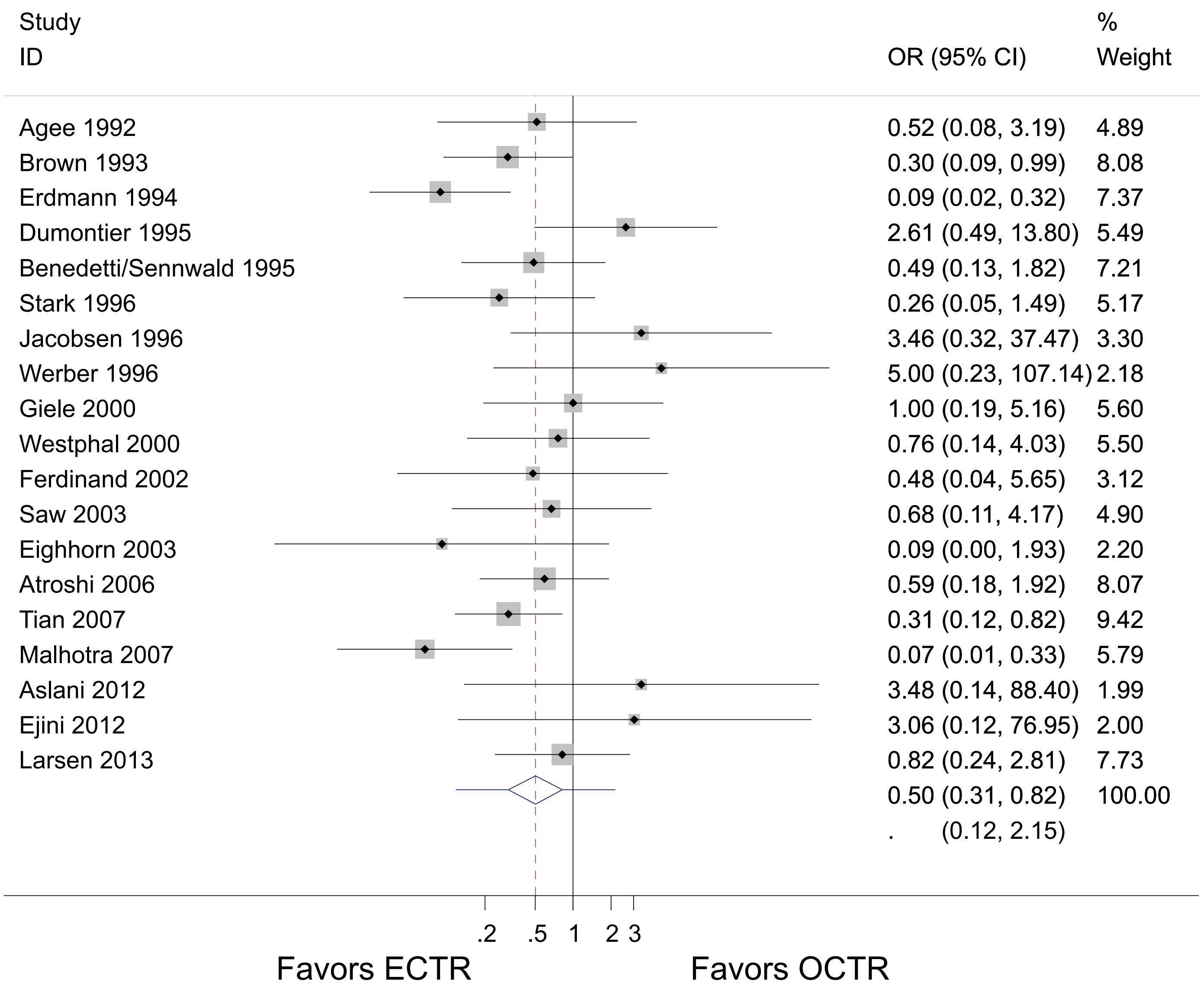

Study
ID
OR (95% CI)
%
Weight
Agee 1992
0.52 (0.08, 3.19)
4.89
Brown 1993
0.30 (0.09, 0.99)
8.08
Erdmann 1994
0.09 (0.02, 0.32)
7.37
Dumontier 1995
2.61 (0.49, 13.80)
5.49
Benedetti/Sennwald 1995
0.49 (0.13, 1.82)
7.21
Stark 1996
0.26 (0.05, 1.49)
5.17
Jacobsen 1996
3.46 (0.32, 37.47)
3.30
Werber 1996
5.00 (0.23, 107.14)
2.18
Giele 2000
1.00 (0.19, 5.16)
5.60
Westphal 2000
0.76 (0.14, 4.03)
5.50
Ferdinand 2002
0.48 (0.04, 5.65)
3.12
Saw 2003
0.68 (0.11, 4.17)
4.90
Eighhorn 2003
0.09 (0.00, 1.93)
2.20
Atroshi 2006
0.59 (0.18, 1.92)
8.07
Tian 2007
0.31 (0.12, 0.82)
9.42
Malhotra 2007
0.07 (0.01, 0.33)
5.79
Aslani 2012
3.48 (0.14, 88.40)
1.99
Ejini 2012
3.06 (0.12, 76.95)
2.00
Larsen 2013
0.82 (0.24, 2.81)
7.73
0.50 (0.31, 0.82)
100.00
. (0.12, 2.15)
.2
.5
1
2
3
Favors ECTR
Favors OCTR

Figure G

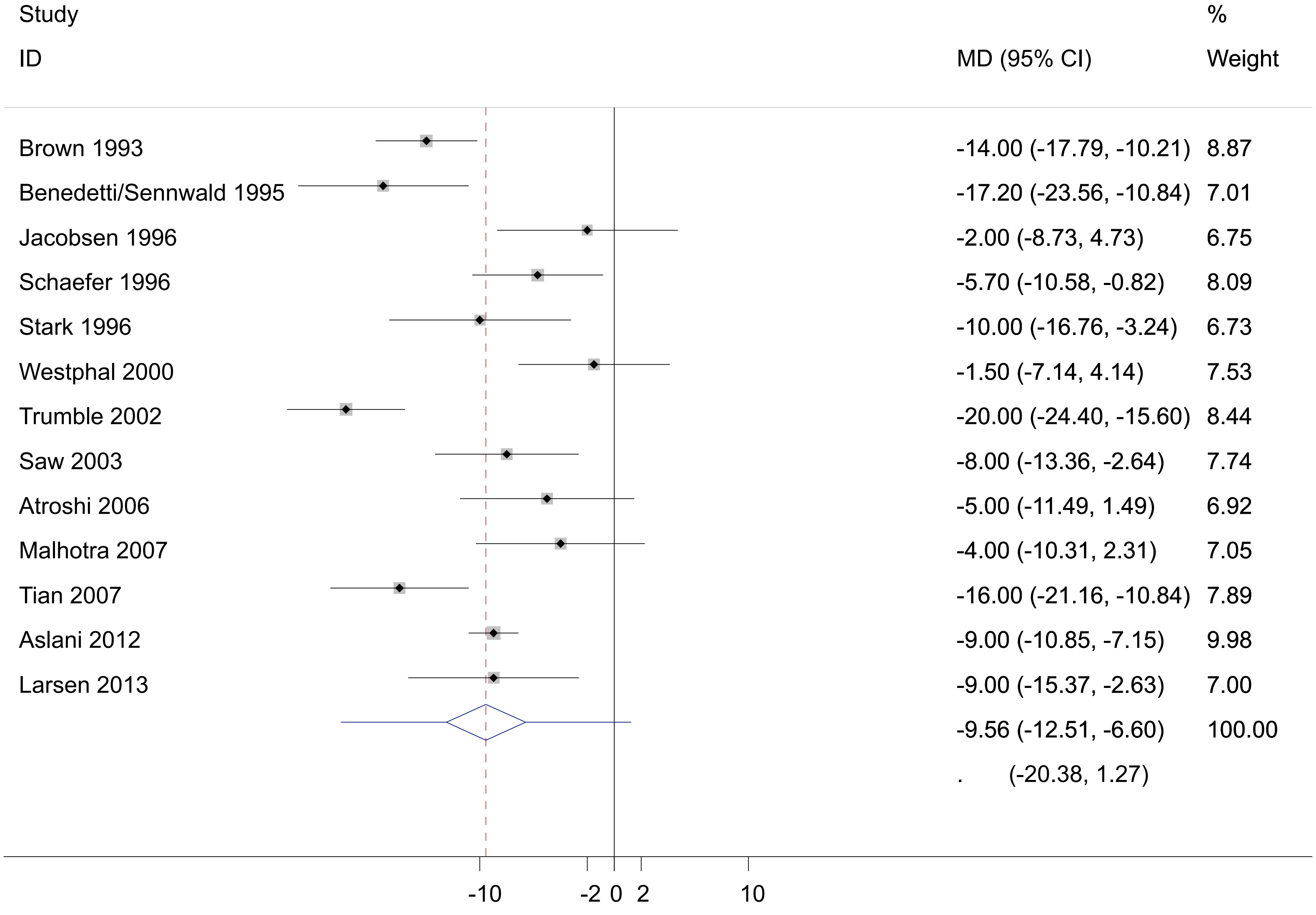

Study
ID
MD (95% CI)
%
Weight
Brown 1993 -14.00 (-17.79, -10.21) 8.87
Benedetti/Sennwald 1995 -17.20 (-23.56, -10.84) 7.01
Jacobsen 1996 -2.00 (-8.73, 4.73) 6.75
Schaefer 1996 -5.70 (-10.58, -0.82) 8.09
Stark 1996 -10.00 (-16.76, -3.24) 6.73
Westphal 2000 -1.50 (-7.14, 4.14) 7.53
Trumble 2002 -20.00 (-24.40, -15.60) 8.44
Saw 2003 -8.00 (-13.36, -2.64) 7.74
Atroshi 2006 -5.00 (-11.49, 1.49) 6.92
Malhotra 2007 -4.00 (-10.31, 2.31) 7.05
Tian 2007 -16.00 (-21.16, -10.84) 7.89
Aslani 2012 -9.00 (-10.85, -7.15) 9.98
Larsen 2013 -9.00 (-15.37, -2.63) 7.00
-9.56 (-12.51, -6.60) 100.00
. (-20.38, 1.27)
-10
-2 0 2
10

Figure H

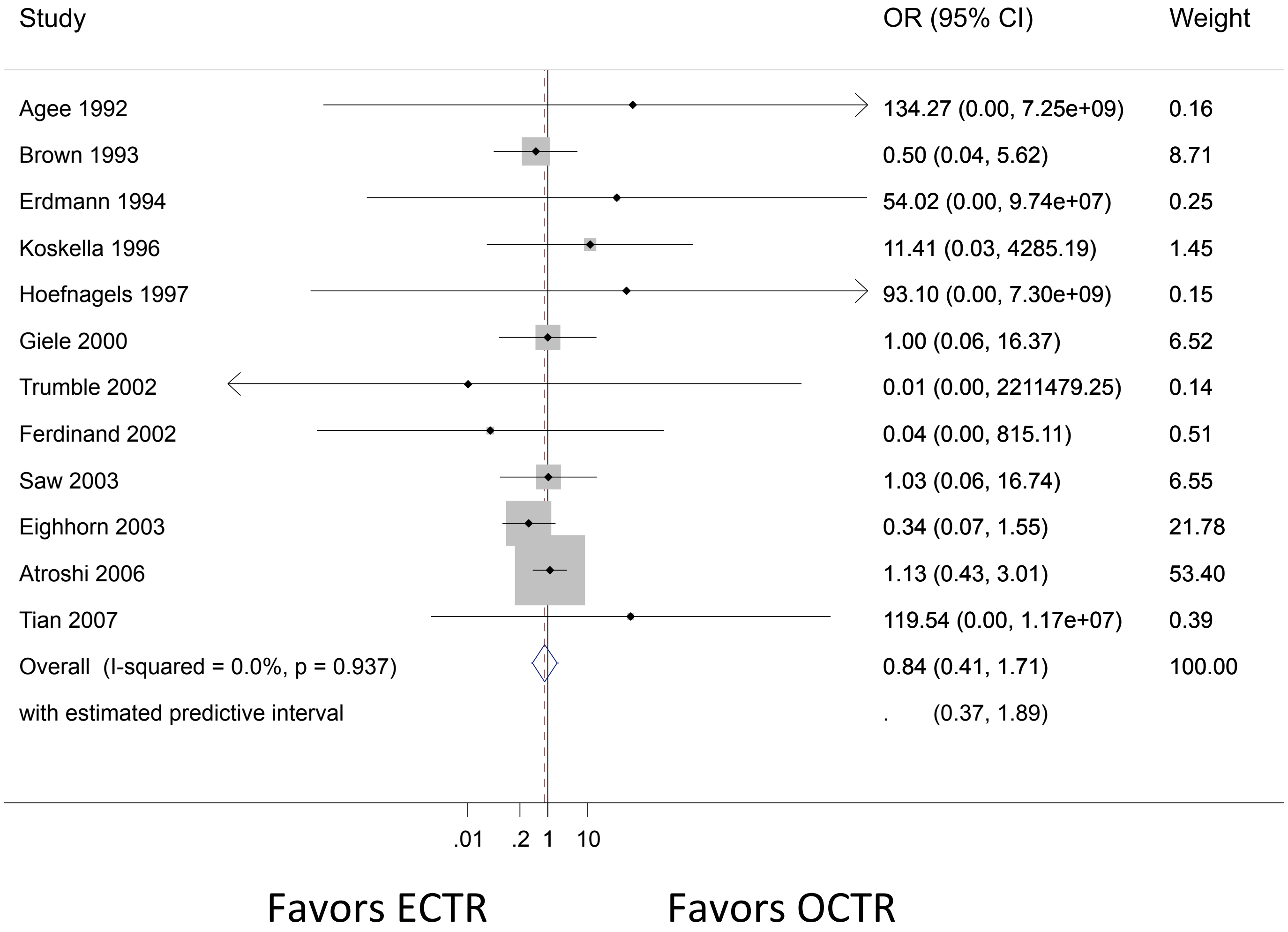
Study
OR (95% CI)
Weight
Agee 1992
134.27 (0.00, 7.25e+09)
0.16
Brown 1993
0.50 (0.04, 5.62)
8.71
Erdmann 1994
54.02 (0.00, 9.74e+07)
0.25
Koskella 1996
11.41 (0.03, 4285.19)
1.45
Hoefnagels 1997
93.10 (0.00, 7.30e+09)
0.15
Giele 2000
1.00 (0.06, 16.37)
6.52
Trumble 2002
0.01 (0.00, 2211479.25)
0.14
Ferdinand 2002
0.04 (0.00, 815.11)
0.51
Saw 2003
1.03 (0.06, 16.74)
6.55
Eighhorn 2003
0.34 (0.07, 1.55)
21.78
Atroshi 2006
1.13 (0.43, 3.01)
53.40
Tian 2007
119.54 (0.00, 1.17e+07)
0.39
Overall (I-squared = 0.0%, p = 0.937)
0.84 (0.41, 1.71)
100.00
with estimated predictive interval
. (0.37, 1.89)
.01
.2
1
10
Favors ECTR
Favors OCTR

Figure I

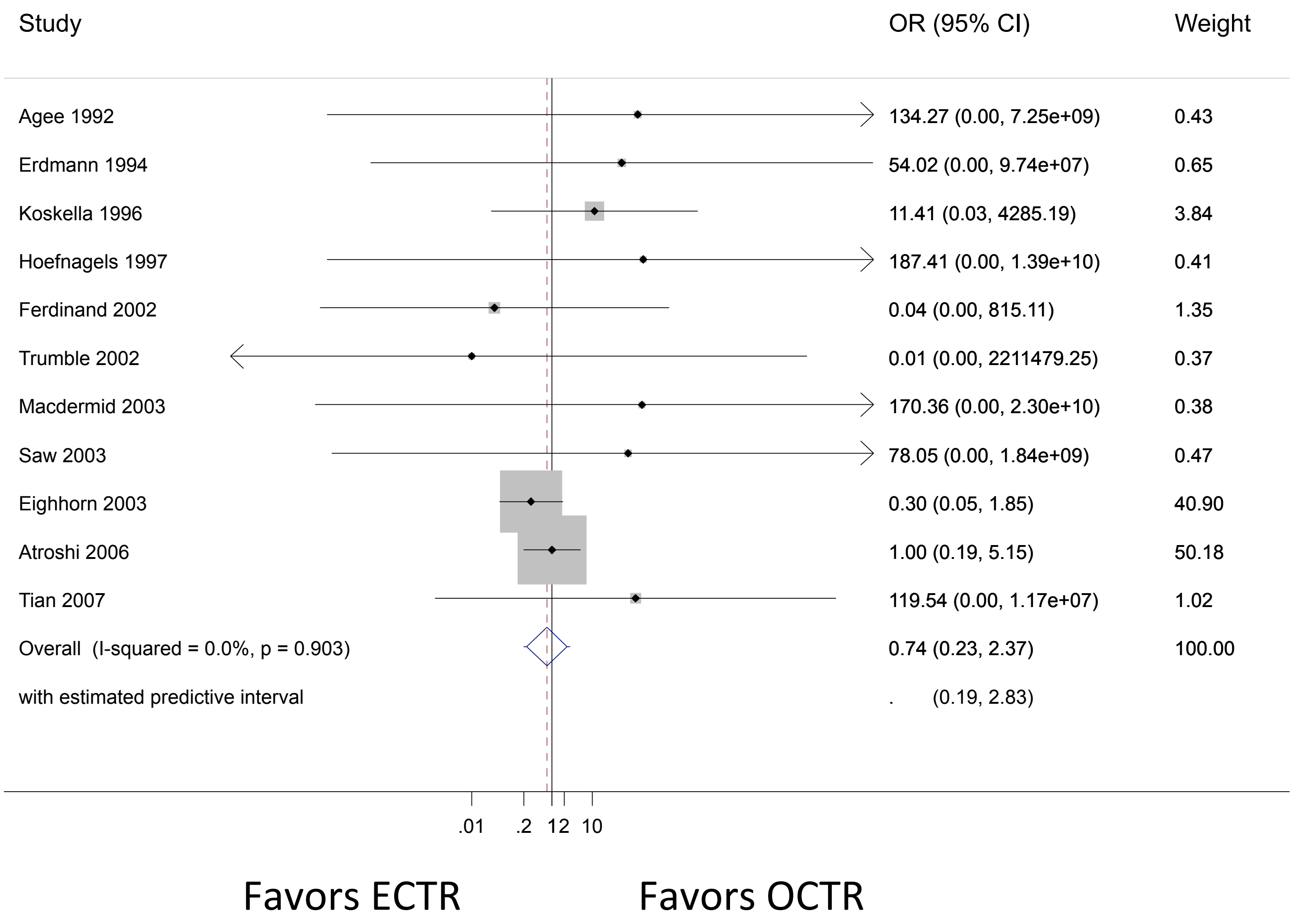

Study
OR (95% CI)
Weight
Agee 1992
134.27 (0.00, 7.25e+09)
0.43
Erdmann 1994
54.02 (0.00, 9.74e+07)
0.65
Koskella 1996
11.41 (0.03, 4285.19)
3.84
Hoefnagels 1997
187.41 (0.00, 1.39e+10)
0.41
Ferdinand 2002
0.04 (0.00, 815.11)
1.35
Trumble 2002
0.01 (0.00, 2211479.25)
0.37
Macdermid 2003
170.36 (0.00, 2.30e+10)
0.38
Saw 2003
78.05 (0.00, 1.84e+09)
0.47
Eighhorn 2003
0.30 (0.05, 1.85)
40.90
Atroshi 2006
1.00 (0.19, 5.15)
50.18
Tian 2007
119.54 (0.00, 1.17e+07)
1.02
Overall (I-squared = 0.0%, p = 0.903)
0.74 (0.23, 2.37)
100.00
with estimated predictive interval
. (0.19, 2.83)
.01
.2
1
2
10
Favors ECTR
Favors OCTR

Figure J

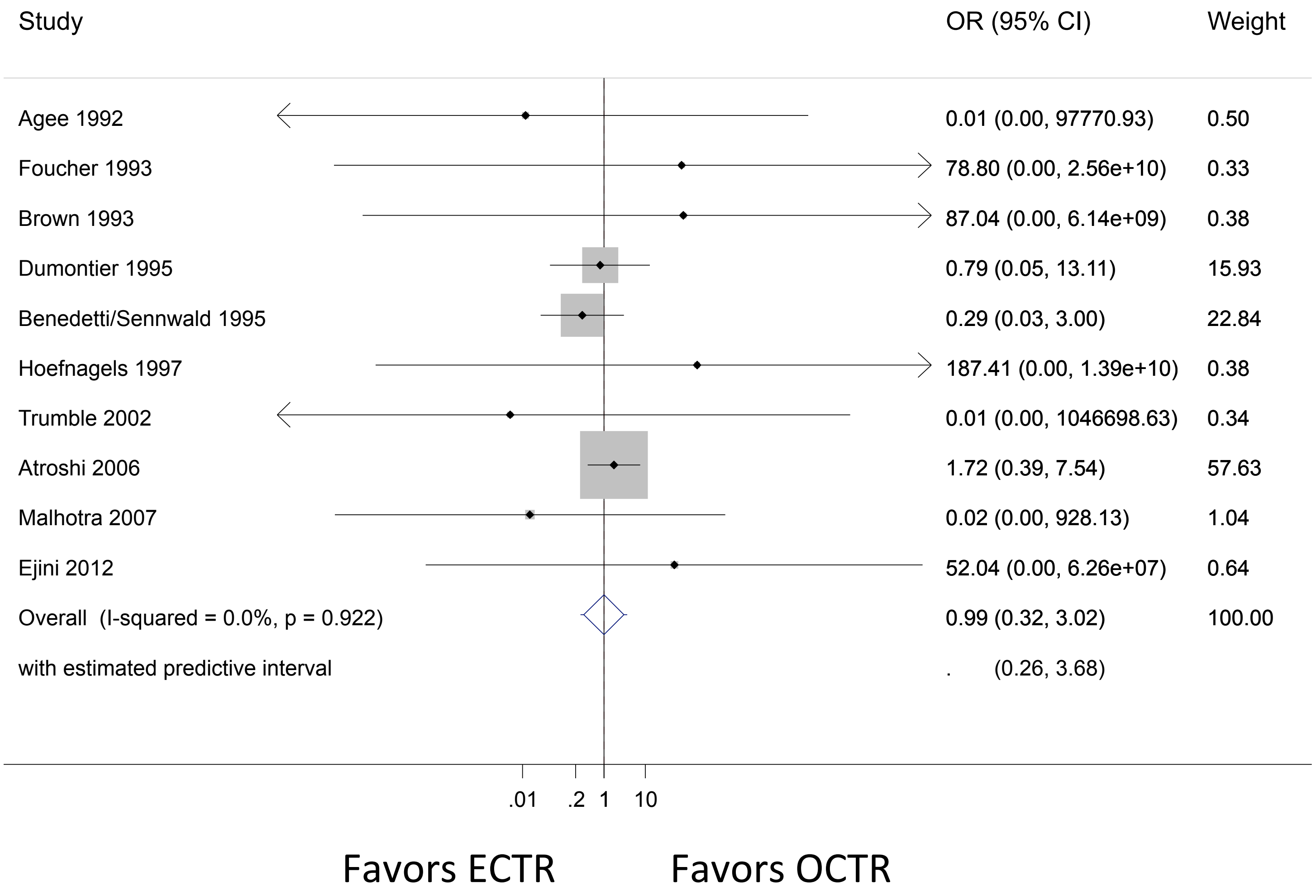
Study
OR (95% CI)
Weight
Agee 1992
0.01 (0.00, 97770.93)
0.50
Foucher 1993
78.80 (0.00, 2.56e+10)
0.33
Brown 1993
87.04 (0.00, 6.14e+09)
0.38
Dumontier 1995
0.79 (0.05, 13.11)
15.93
Benedetti/Sennwald 1995
0.29 (0.03, 3.00)
22.84
Hoefnagels 1997
187.41 (0.00, 1.39e+10)
0.38
Trumble 2002
0.01 (0.00, 1046698.63)
0.34
Atroshi 2006
1.72 (0.39, 7.54)
57.63
Malhotra 2007
0.02 (0.00, 928.13)
1.04
Ejini 2012
52.04 (0.00, 6.26e+07)
0.64
Overall (I-squared = 0.0%, p = 0.922)
0.99 (0.32, 3.02)
100.00
with estimated predictive interval
. (0.26, 3.68)
.01
.2
1
10
Favors ECTR
Favors OCTR

Figure K

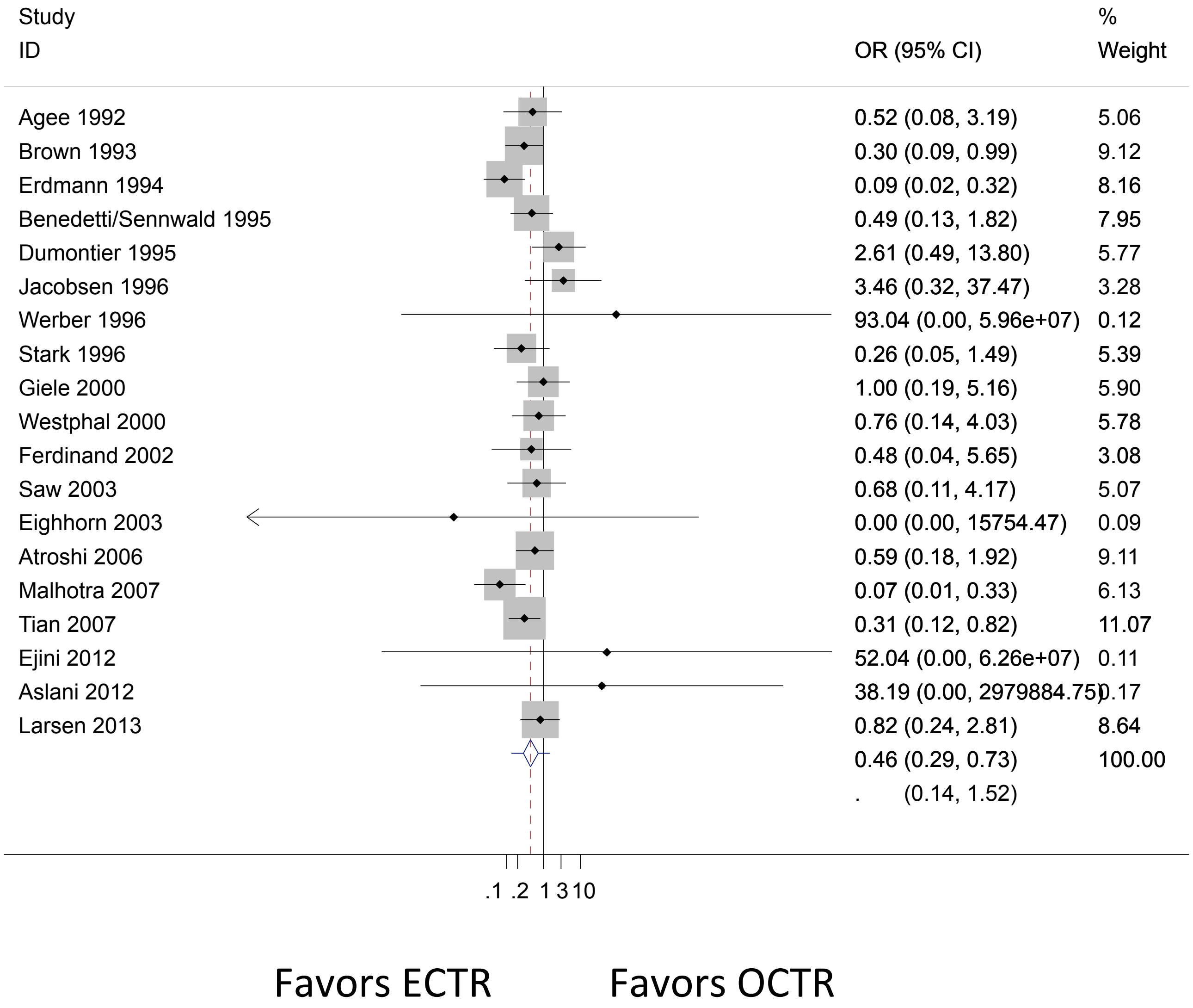
Study
ID
OR (95% CI)
%
Weight
Agee 1992 0.52 (0.08, 3.19) 5.06
Brown 1993 0.30 (0.09, 0.99) 9.12
Erdmann 1994 0.09 (0.02, 0.32) 8.16
Benedetti/Sennwald 1995 0.49 (0.13, 1.82) 7.95
Dumontier 1995 2.61 (0.49, 13.80) 5.77
Jacobsen 1996 3.46 (0.32, 37.47) 3.28
Werber 1996 93.04 (0.00, 5.96e+07) 0.12
Stark 1996 0.26 (0.05, 1.49) 5.39
Giele 2000 1.00 (0.19, 5.16) 5.90
Westphal 2000 0.76 (0.14, 4.03) 5.78
Ferdinand 2002 0.48 (0.04, 5.65) 3.08
Saw 2003 0.68 (0.11, 4.17) 5.07
Eighhorn 2003 0.00 (0.00, 15754.47) 0.09
Atroshi 2006 0.59 (0.18, 1.92) 9.11
Malhotra 2007 0.07 (0.01, 0.33) 6.13
Tian 2007 0.31 (0.12, 0.82) 11.07
Ejini 2012 52.04 (0.00, 6.26e+07) 0.11
Aslani 2012 38.19 (0.00, 2979884.75) 0.17
Larsen 2013 0.82 (0.24, 2.81) 8.64
0.46 (0.29, 0.73) 100.00
. (0.14, 1.52)
.1 .2 1 3 10
Favors ECTR
Favors OCTR

Figure L

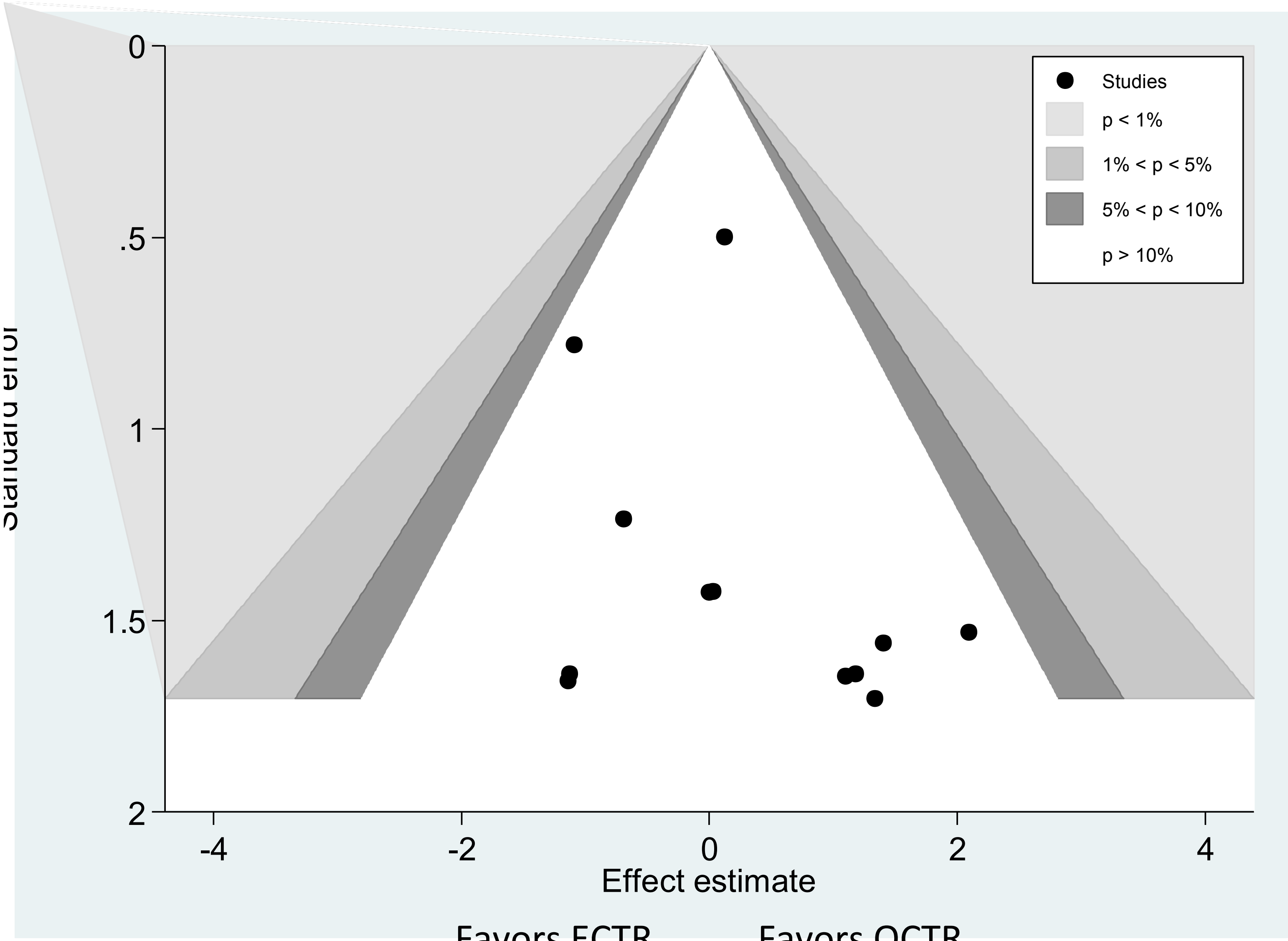

Favors ECTR Favors OCTR

Figure M

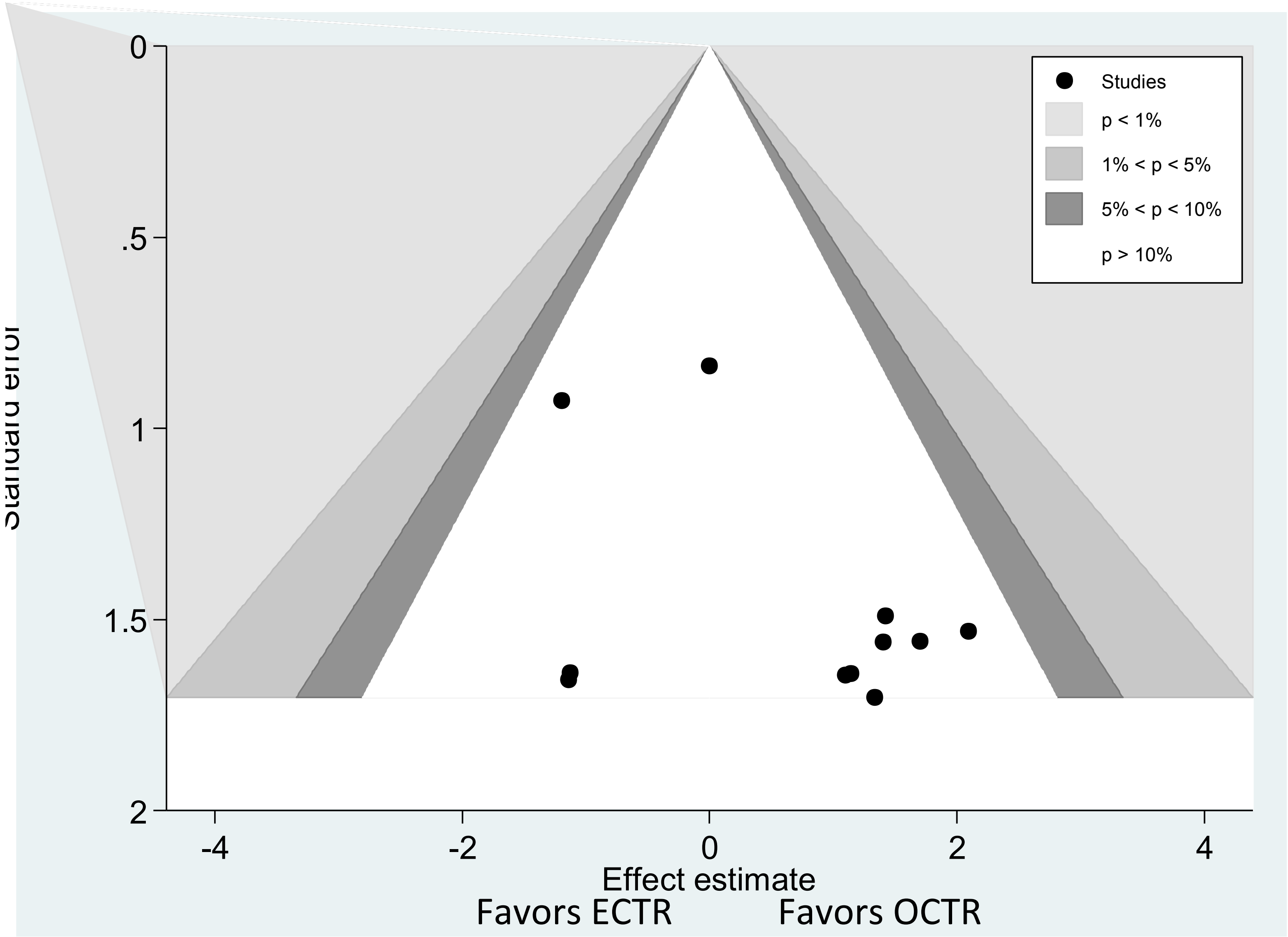
Standard error
Effect estimate
Studies
p < 1%
1% < p < 5%
5% < p < 10%
p > 10%
Favors ECTR
Favors OCTR

Figure N

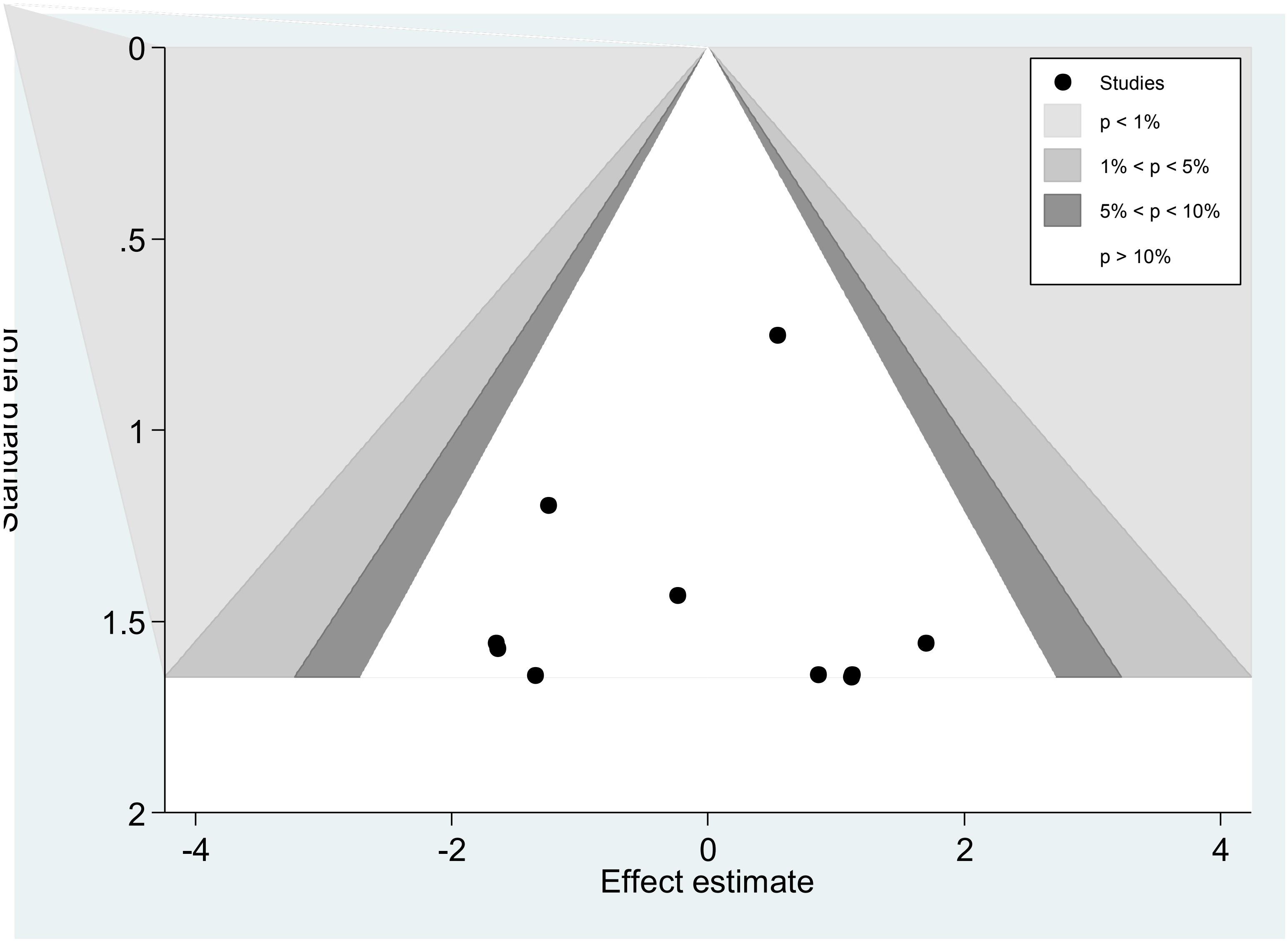

Favors ECTR Favors OCTR

Figure O

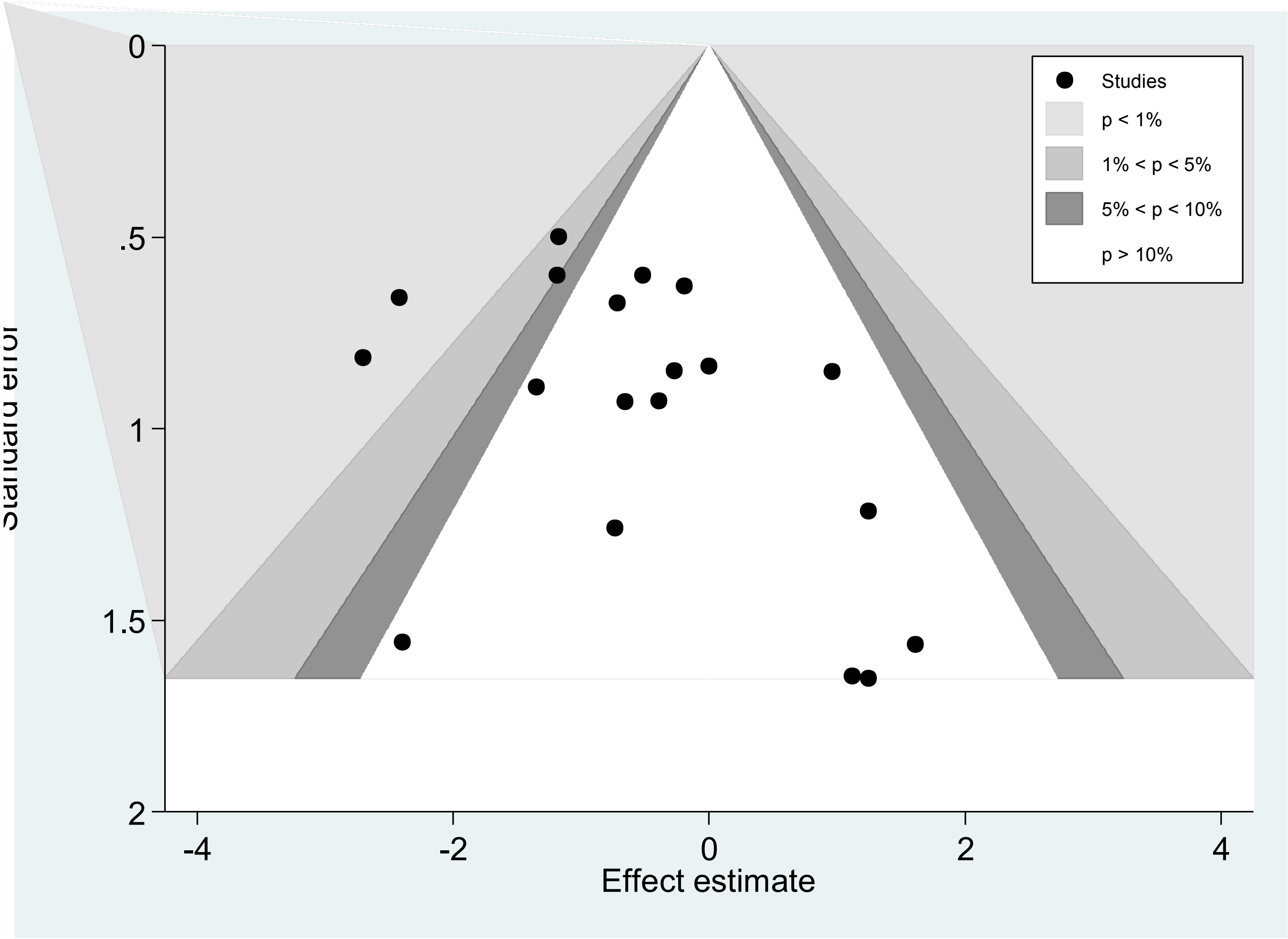

Favors ECTR Favors OCTR

Figure P

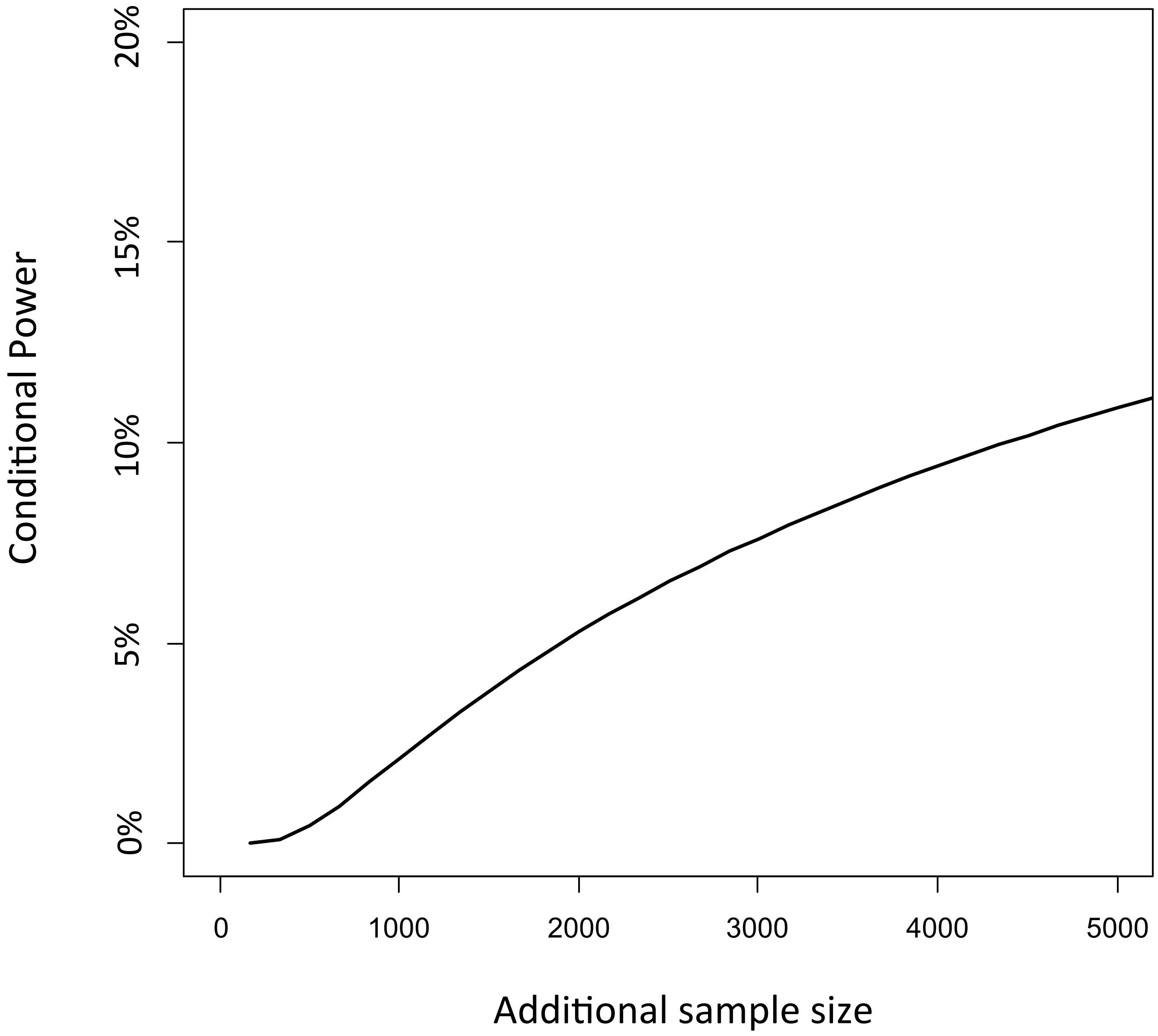

20%
15%
10%
5%
0%
Conditional Power
0
1000
2000
3000
4000
5000
Additional sample size

Figure Q

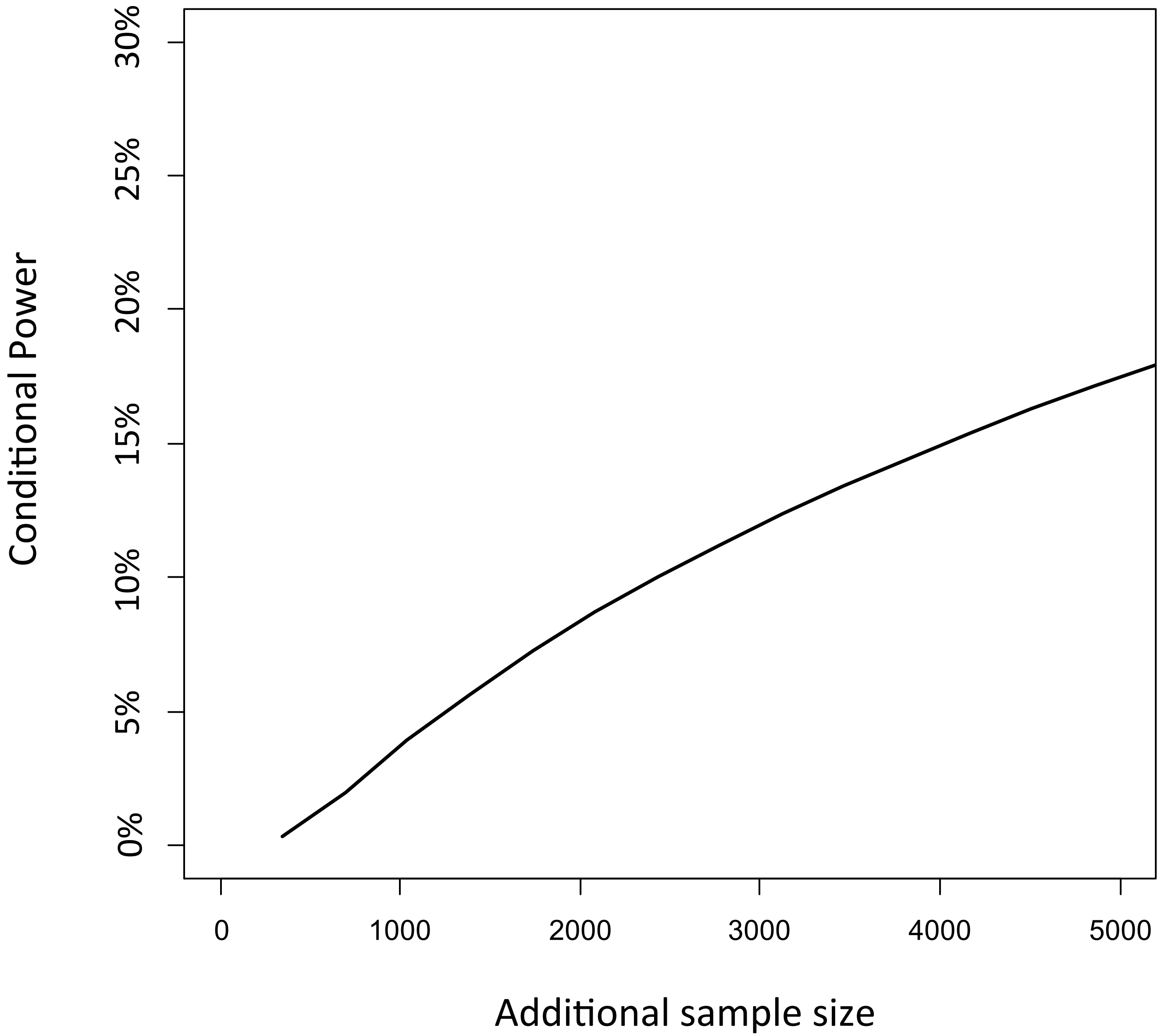

Conditional Power
30%
25%
20%
15%
10%
5%
0%
0
1000
2000
3000
4000
5000
Additional sample size

Figure R

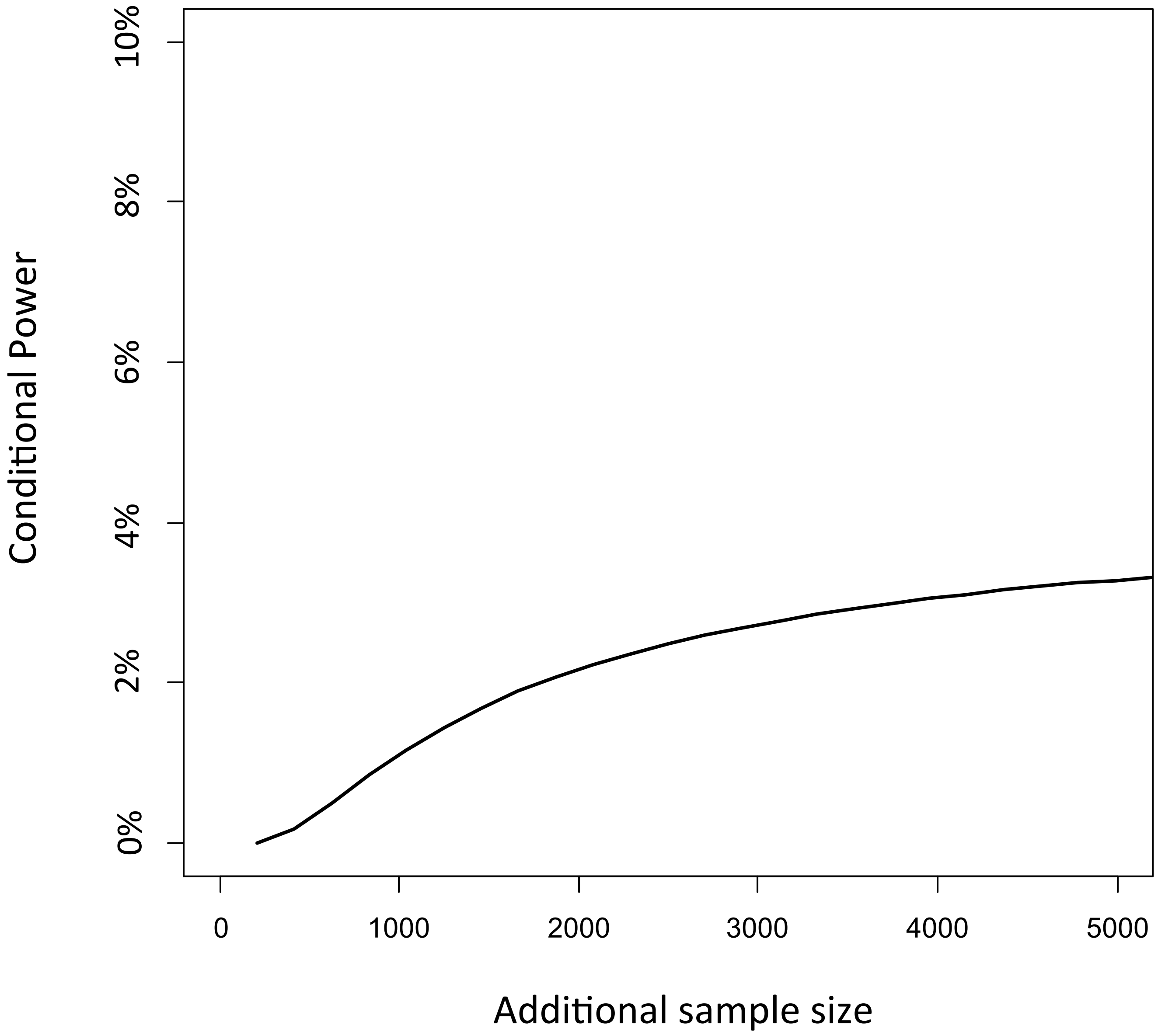

Conditional Power
10%
8%
6%
4%
2%
0%
0
1000
2000
3000
4000
5000
Additional sample size

Figure S

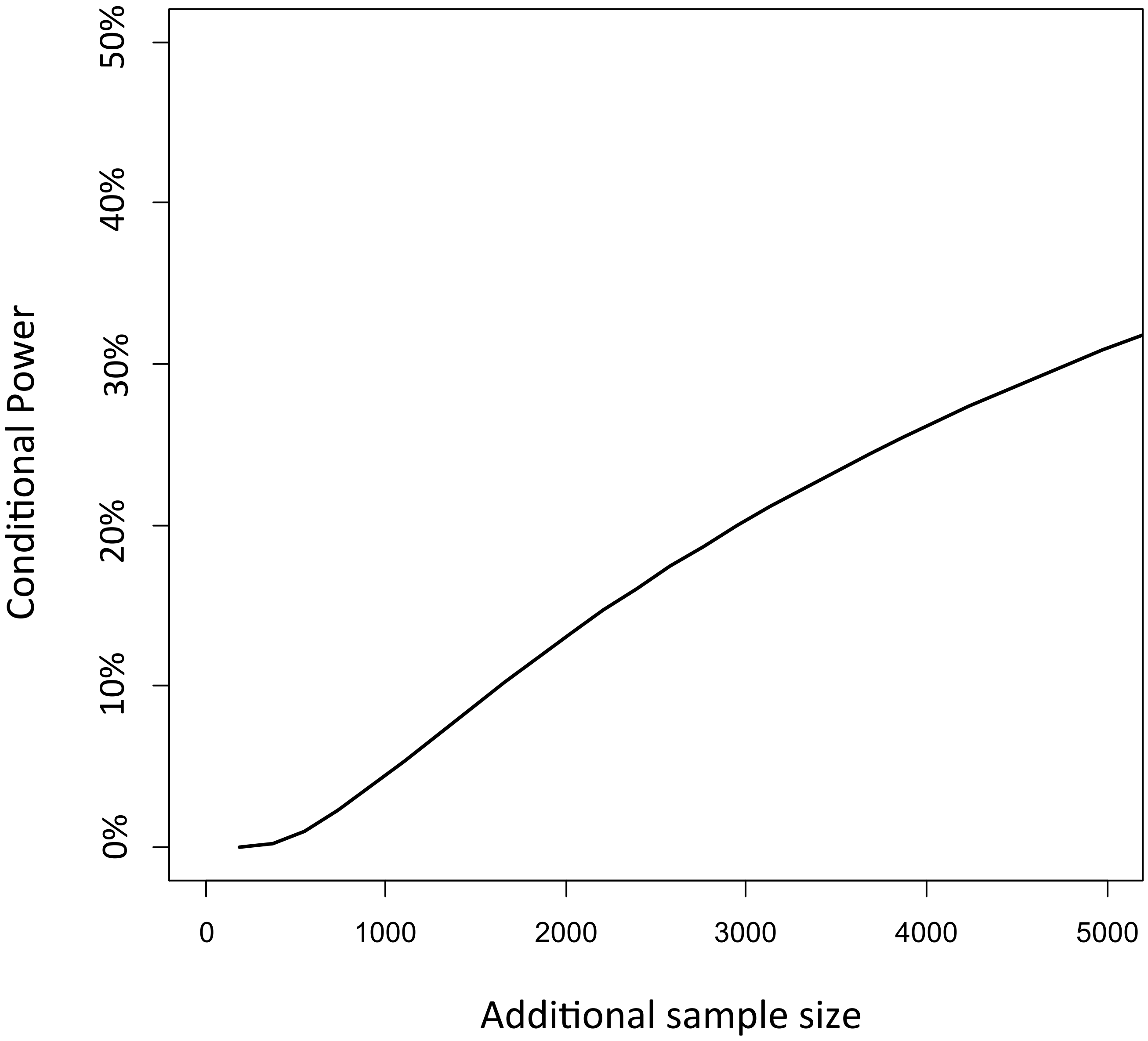

Conditional Power
0%
10%
20%
30%
40%
50%
0
1000
2000
3000
4000
5000
Additional sample size

Figure T

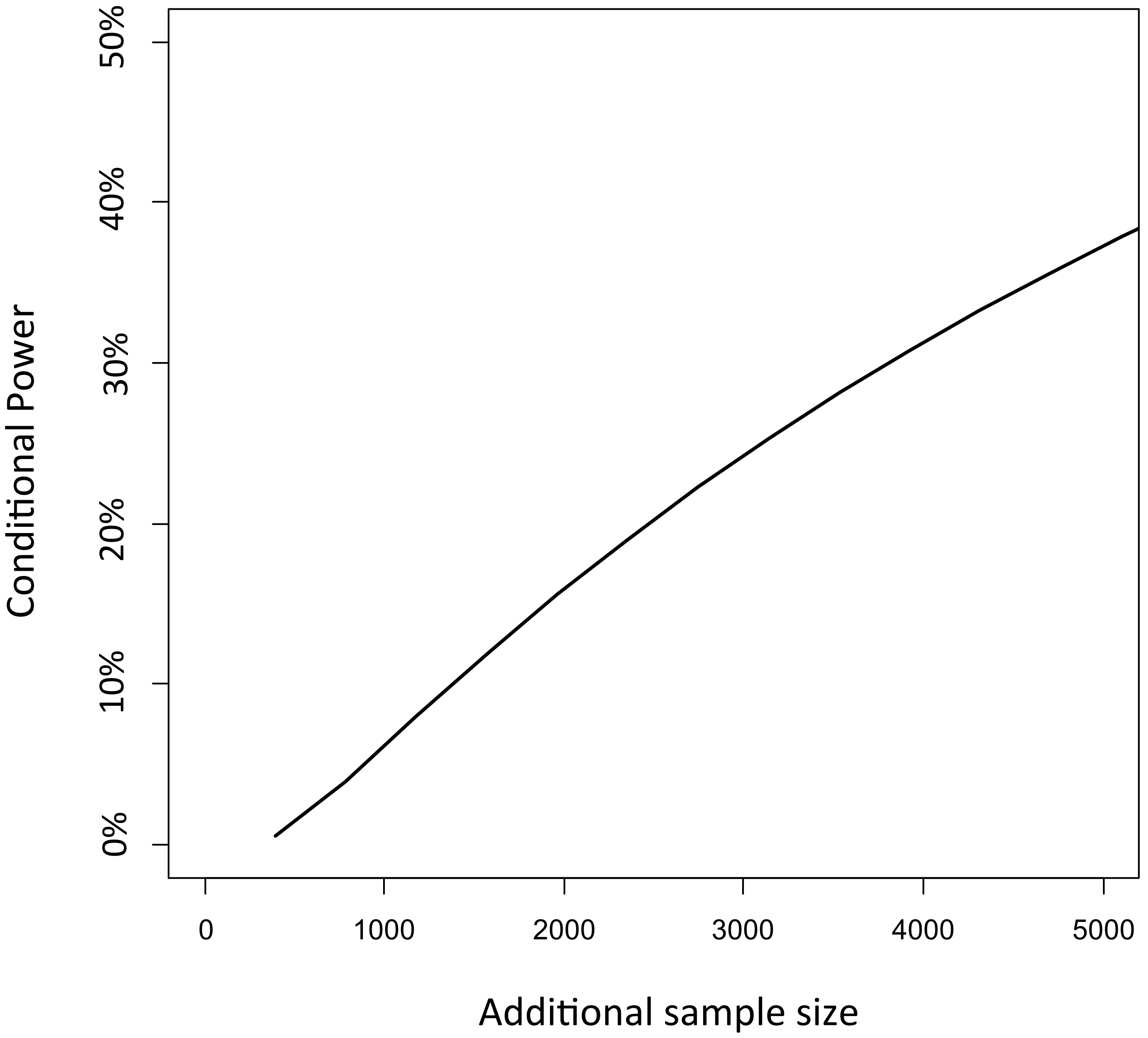

Conditional Power
50%
40%
30%
20%
10%
0%
0
1000
2000
3000
4000
5000
Additional sample size

Figure U

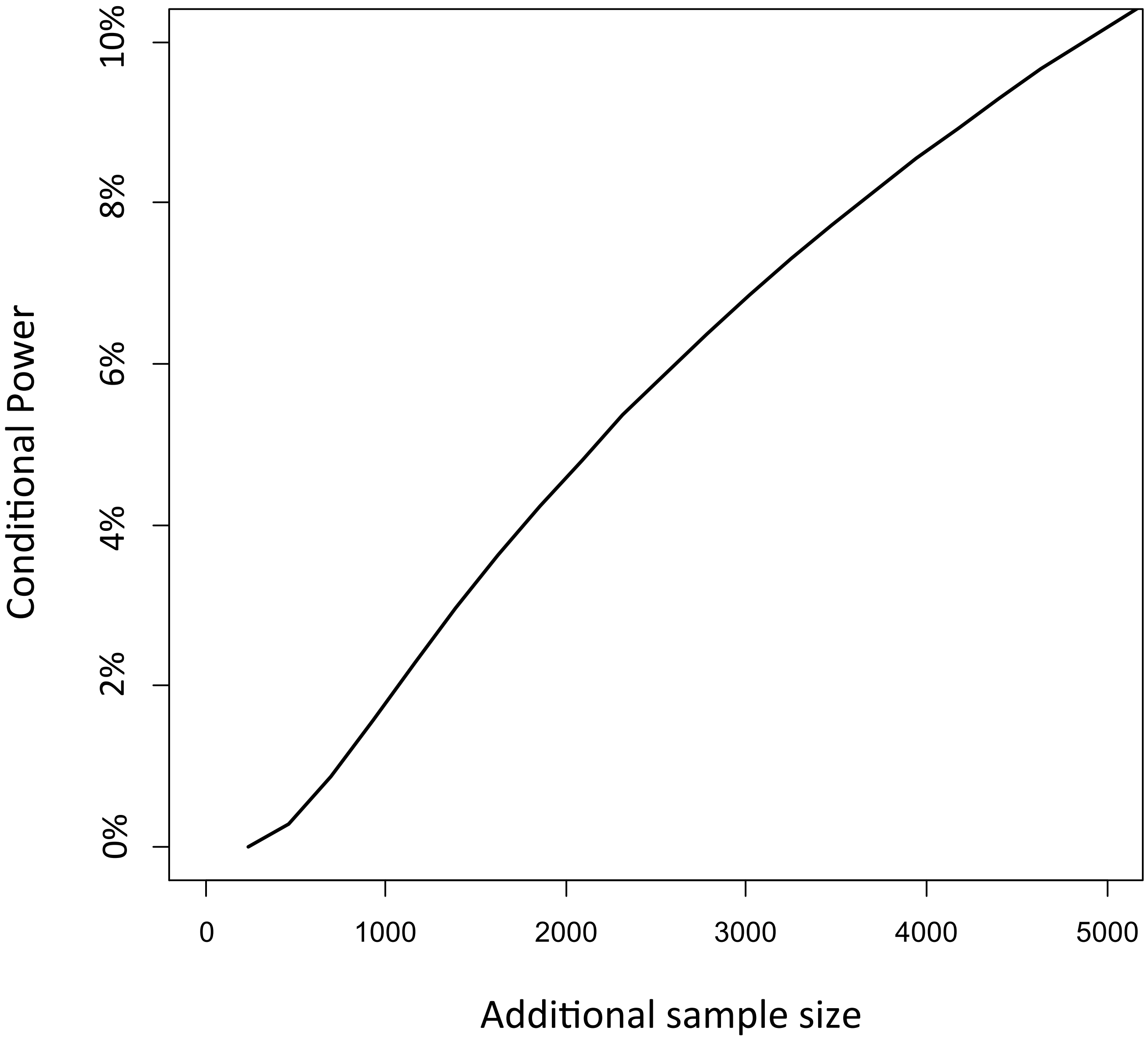

10%
8%
6%
4%
2%
0%
Conditional Power
0
1000
2000
3000
4000
5000
Additional sample size

Figure V

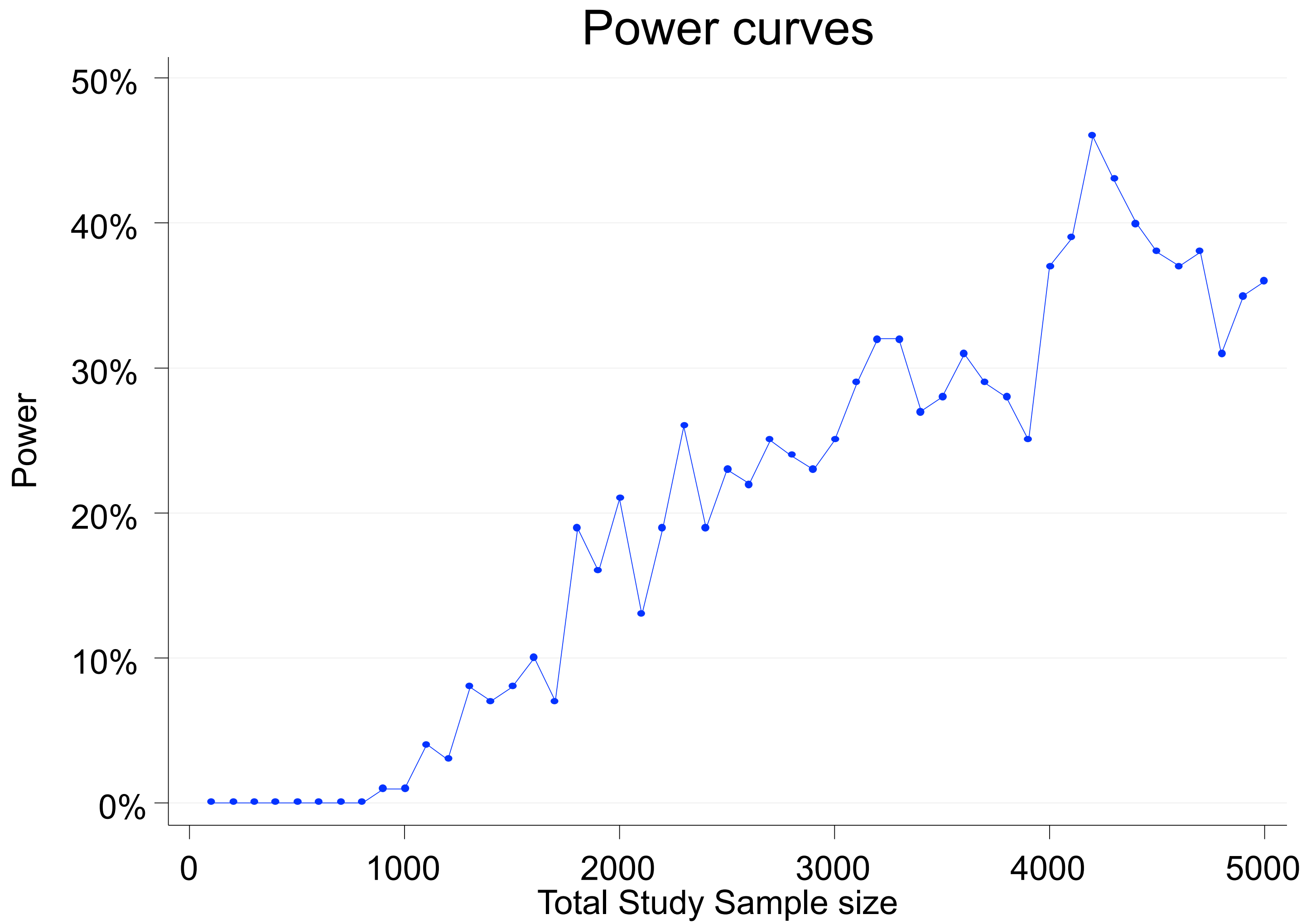

Power curves
50%
40%
30%
20%
10%
0%
Power
0
1000
2000
3000
4000
5000
Total Study Sample size

Figure W

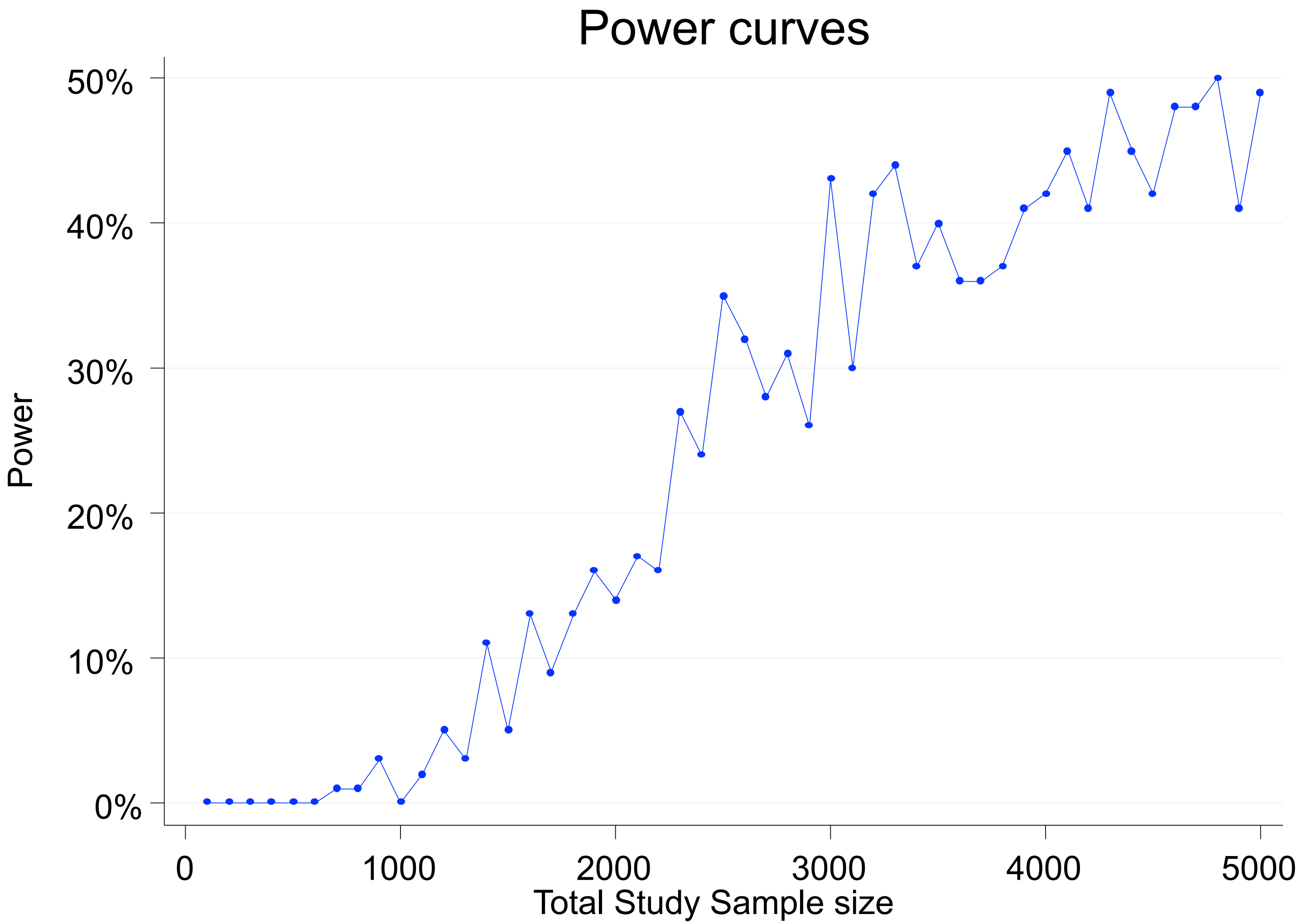

Power curves
50%
40%
30%
20%
10%
0%
Power
0
1000
2000
3000
4000
5000
Total Study Sample size

Figure X

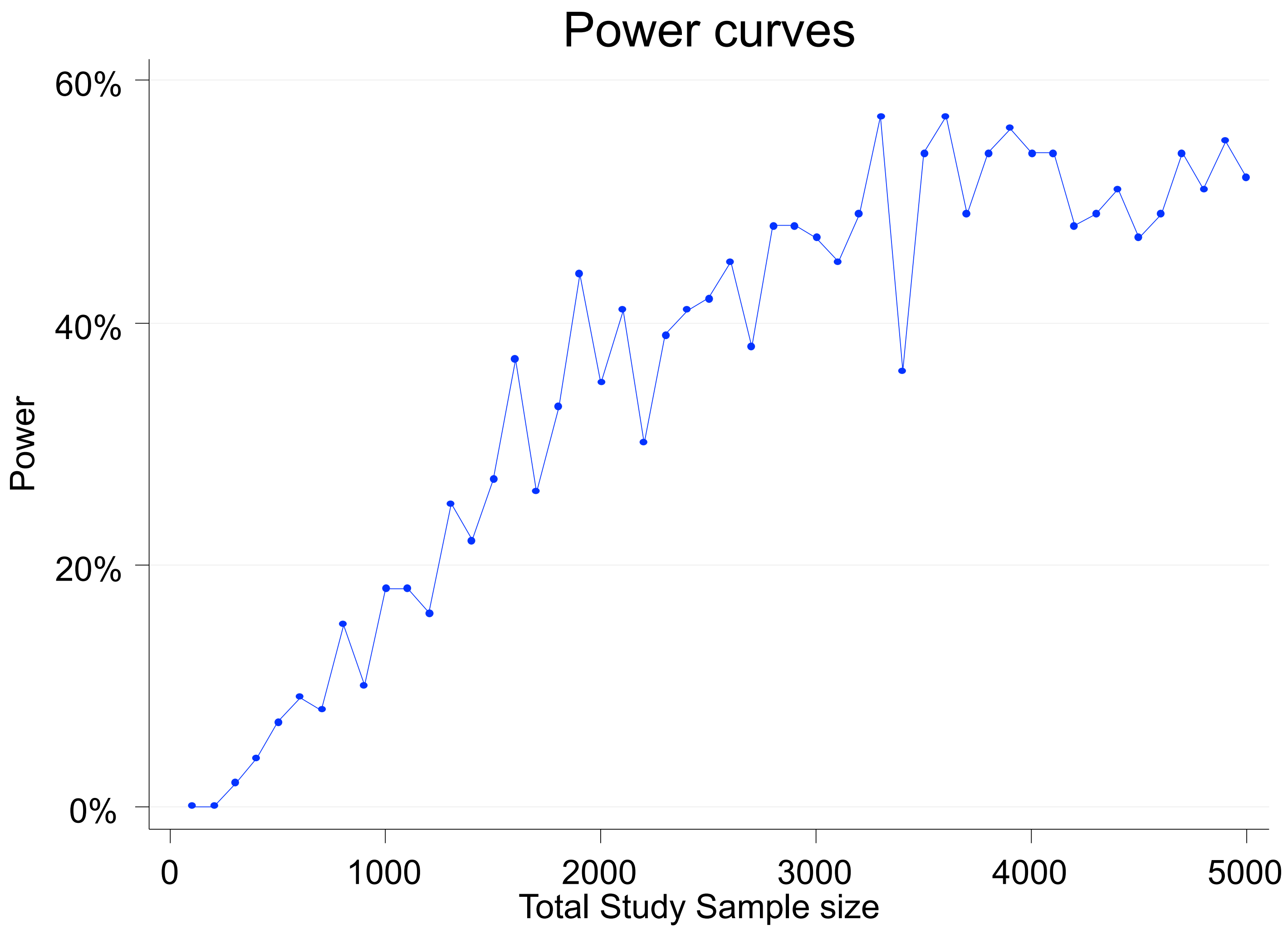

Power curves
60%
40%
20%
0%
Power
0
1000
2000
3000
4000
5000
Total Study Sample size

Figure Y

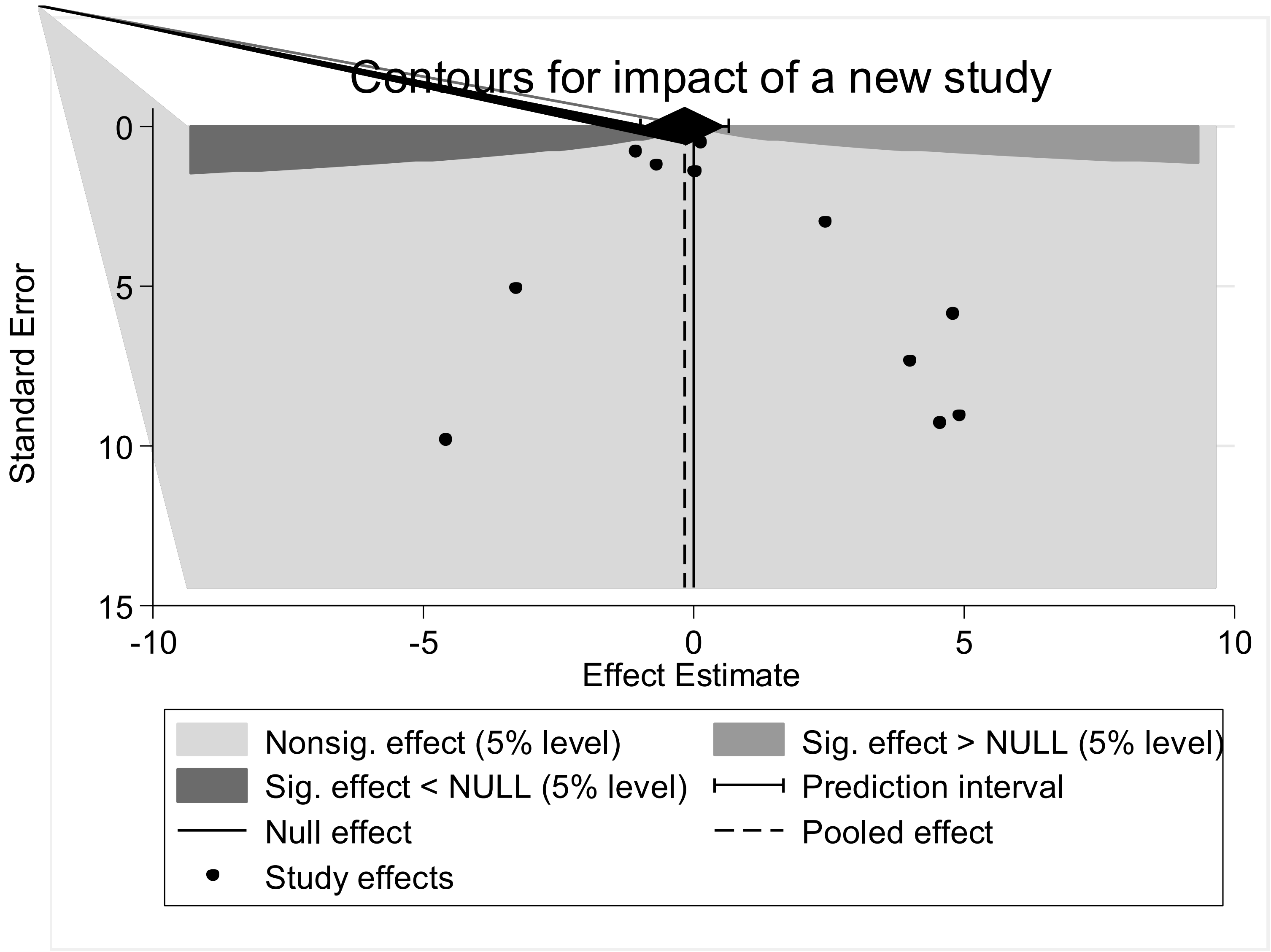

Contours for impact of a new study
Standard Error
0
5
10
15
-10
-5
0
5
10
Effect Estimate
Nonsig. effect (5% level)
Sig. effect > NULL (5% level)
Sig. effect < NULL (5% level)
Prediction interval
Null effect
Pooled effect
Study effects

Figure Z

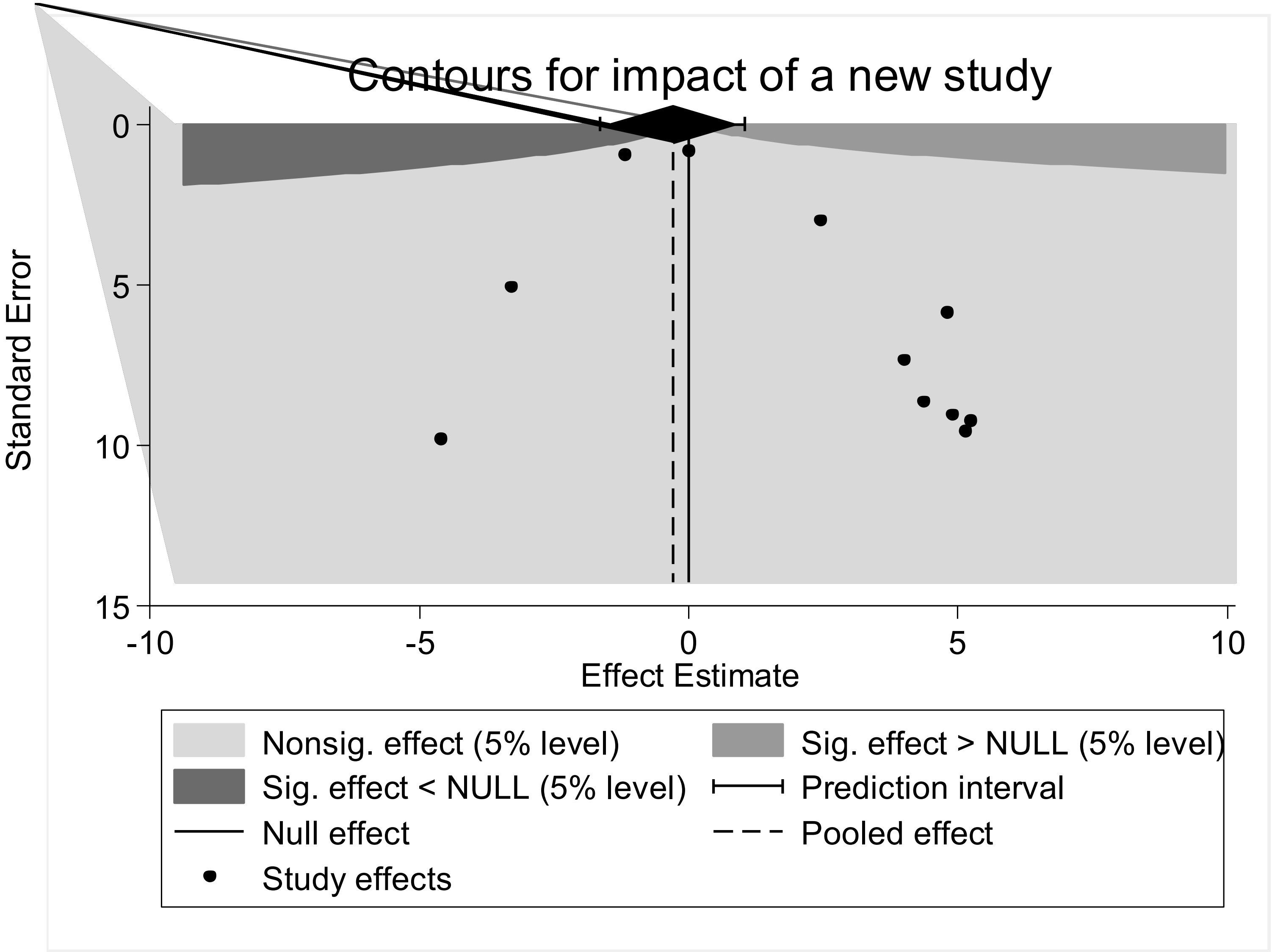

Contours for impact of a new study
Standard Error
0
5
10
15
-10
-5
0
5
10
Effect Estimate
Nonsig. effect (5% level)
Sig. effect > NULL (5% level)
Sig. effect < NULL (5% level)
Prediction interval
Null effect
Pooled effect
Study effects

Figure AA

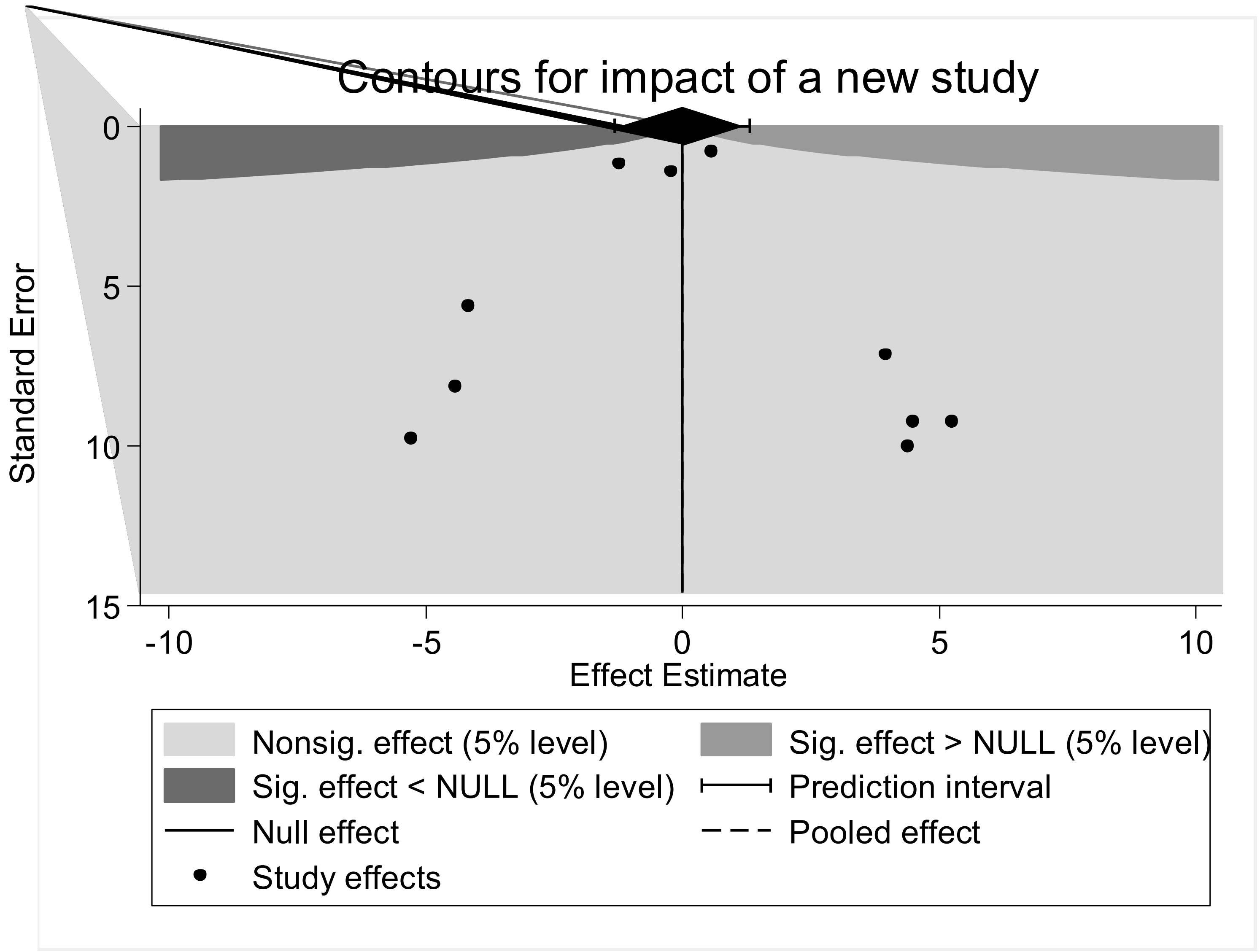
Contours for impact of a new study
Standard Error
0
5
10
15
-10
-5
0
5
10
Effect Estimate
Nonsig. effect (5% level)
Sig. effect > NULL (5% level)
Sig. effect < NULL (5% level)
Prediction interval
Null effect
Pooled effect
Study effects

Figure AB

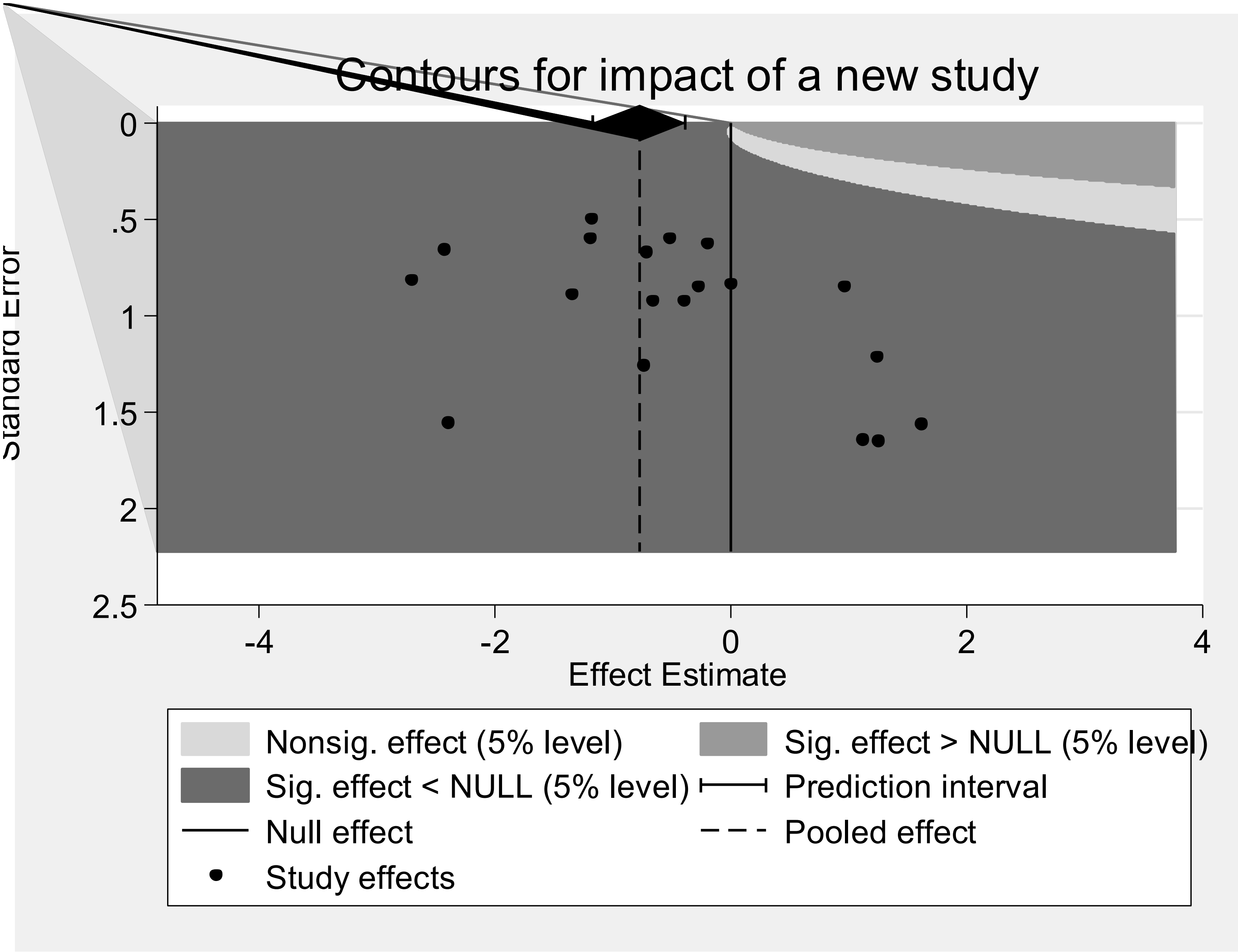
Contours for impact of a new study
Standard Error
0
.5
1
1.5
2
2.5
-4
-2
0
2
4
Effect Estimate
Nonsig. effect (5% level)
Sig. effect > NULL (5% level)
Sig. effect < NULL (5% level)
Prediction interval
Null effect
Pooled effect
Study effects
