# Supplementary figures and images for: Endoscopic and Open Release Similarly Safe for the Treatment of Carpal Tunnel Syndrome. A Systematic Review and Meta-Analysis
Source: PLoS One. 2015 Dec 16;10(12):e0143683. doi: 10.1371/journal.pone.0143683 (PMC4682940; doi:10.1371/journal.pone.0143683)

Figure A

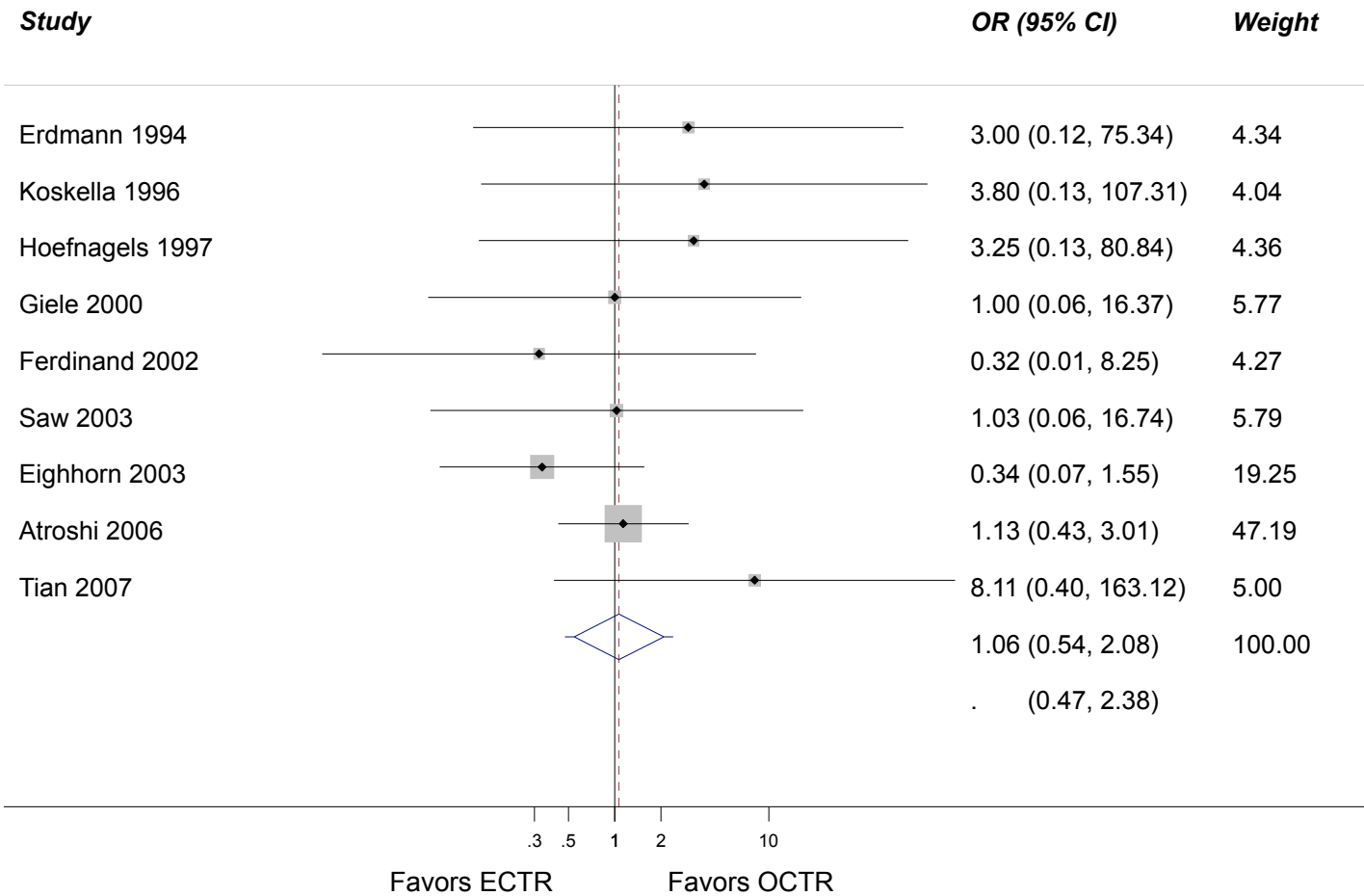

Figure B

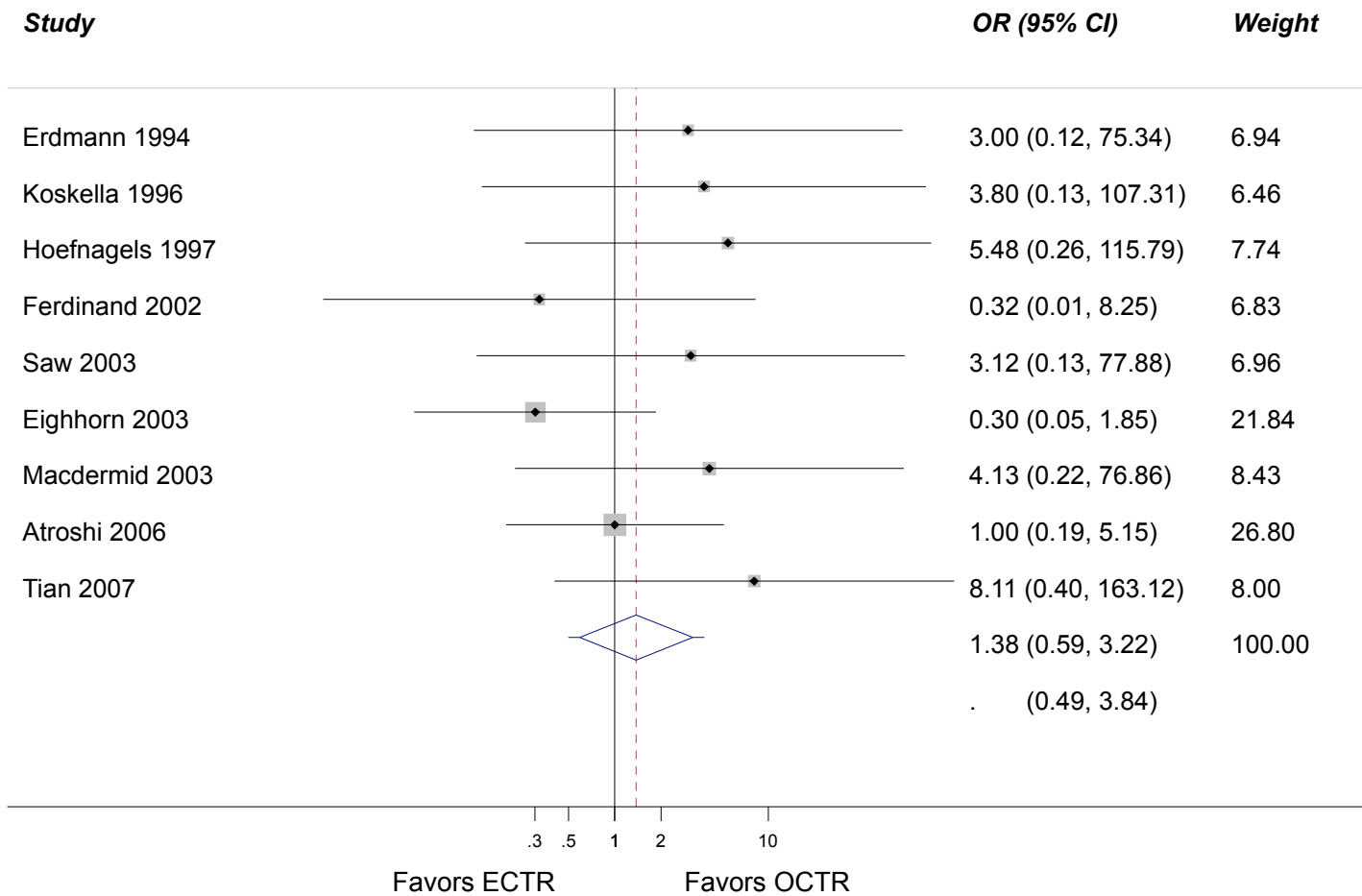

Figure C

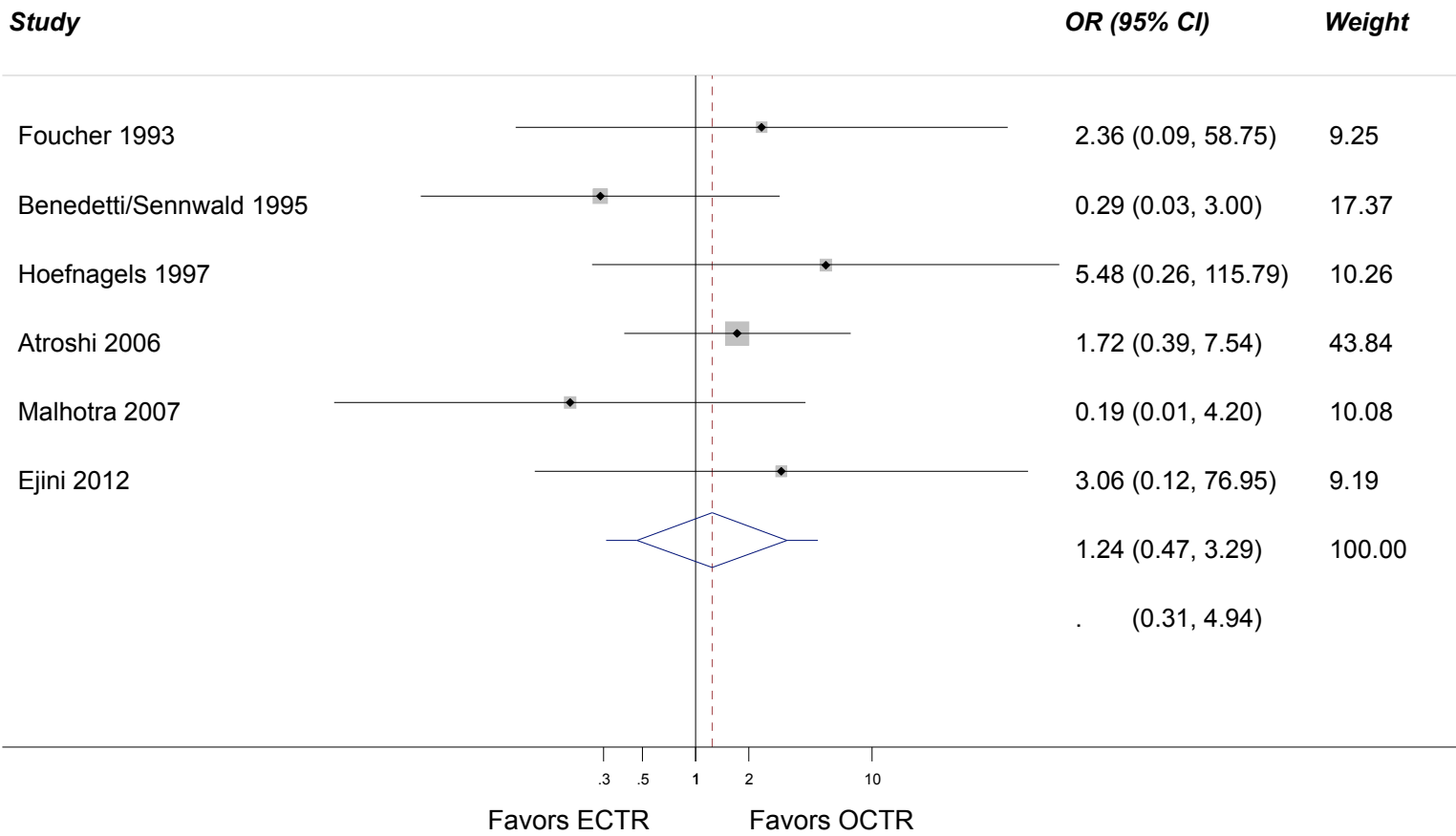

Figure D

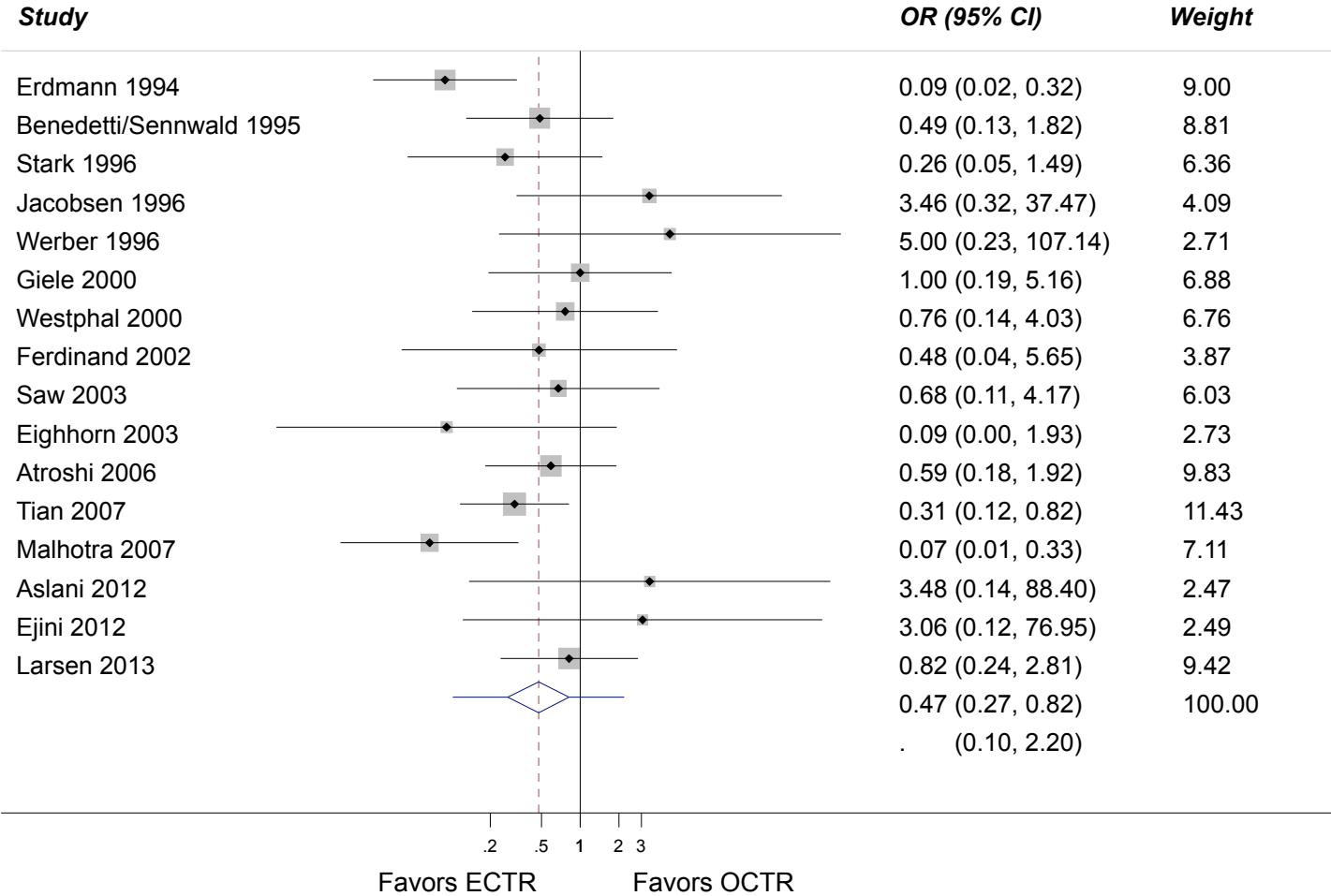

Supplement: S3 File — Continuity correction of 0.5 has been applied for rare events. Studies with high risk of bias regarding allocation concealment have been excluded. (PDF) [file pone.0143683.s007.pdf]
