# Supplementary material for: Endoscopic and Open Release Similarly Safe for the Treatment of Carpal Tunnel Syndrome. A Systematic Review and Meta-Analysis
Source: PLoS One. 2015 Dec 16;10(12):e0143683. doi: 10.1371/journal.pone.0143683 (PMC4682940; doi:10.1371/journal.pone.0143683)
Supplement: S1 Table — (PDF) [file pone.0143683.s008.pdf]

	CHARACTERISTICS					
	intervention	age	Sample size	bilateral	Publication	latest follow up
**Agee 1992**	one portal / Agee techique	NR	122 participants/ 147 hands	25 bilateral	Full publication	**6m**
**Aslani 2012**	two portal / Chow technique	mean 54.2 (range 30–65)	68 participants (+28 mini-open)	8 bilateral (out of total 96)	Full publication	**4m**
**Atroshi 2009**	two portal / Chow technique	mean 44 (range 25-60)	128 participants	no bilateral	Full publication	**5y**
**Benedetti 1996 / Sennwald 1995**	one portal / Agee techique	ECTR mean age 48.6 years / OCTR mean age 57 years	47 participants	NR	Full publication	**13m**
**Brown 1993**	two portal / Chow technique	mean 55 (range 25-87)	151 participants /169 hands	18 bilateral	Full publication	**3m**
**Dumortier 1995**	two portal / Chow technique	men mean 50.7/ women mean 53.4	96 participants	NR	Full publication	**3m**
**Eighghorn 2003**	two portal / Chow technique	NR	60 hands OCTR, 128 hands ECTR		Full publication	**12m**
**Ejiri 2012**	one portal techique	mean 59 (range 40-82)	79 participants/101 hands	22 bilateral	Full publication	**3m**
**Erdman 1994**	two portal / Chow technique	ECTR mean 52.7/OCTR mean 54.1	71 participants /105 hands	34 bilateral	Full publication	**12m**
**Ferdinand 2002**	one portal / Agee techique	mean 54.9	25 participants/50 hands	only bilateral	Full publication	**12m**
**Foucher 1993**	one portal / Agee techique	NR	251 hands		Full publication	**3m**
**Giele 2000**	NR	51 years (range 27- 91)	60 participants /120 hands	only bilateral	abstract	**3m**
**Hoefnagels 1997**	one portal / Agee techique	51 years (range 21-87)	178 participants	no bilateral	Full publication	**3m**
**Incoll 2004**	NR	NR	20 participants/40 hands	only bilateral	abstract	**1.5m**
**Jacobsen 1996**	two portal / Chow technique	mean 46 (range 24-59)	29 participants/31 hands	4 bilateral	Full publication	**6m**
**Koskella 1996**	NR	mean 50.4	16 participants/17 hands	1 bilateral	abstract	**12m**
**Larsen 2013**	one portal / Menon techique	ECTR mean 54/ OCTR mean 54	60 participants	no bilateral	Full publication	**6m**
**Mac Dermid 2003**	two portal / Chow technique	ECTR mean 45/ OCTR mean 53	123 participants	NR	Full publication	**3m**
**Malhotra 2007**	one portal / Agee techique	ECTR mean 44.6/ OCTR mean 45.3	60 participants /61 hands	1 bilateral	Full publication	**6m**
**Saw 2003**	one portal / Agee techique	ECTR mean 54/ OCTR mean 50	150 participants	no bilateral	Full publication	**3m**
**Schaefer 1996**	one portal / Agee techique	mean 53	101 participants	NR	Full publication	**9m**
**Stark 1996**	one portal / Agee techique	mean 53	20 participants/40 hands	only bilateral	Full publication	**8m**
**Tian 2007**	one portal / Okutsu techique	mean 52 (range 30-70)	62 participants/70 hands	8 bilateral	Full publication	**24m**
**Trumble 2002**	one portal / Agee techique	mean 56 (range 24-74)	161 participants /209 hands	45 bilateral	Full publication	**12m**
**Tüzüner 2008**	one portal / Menon techique	mean 52 (range 38-60)	13 participants /16 hands	3 bilateral	Full publication	**NR**
**Werber 1996**	one portal techique	NR	90 participants	NR	abstract	**6m**
**Westphal 2000**	one portal / ENDO-CARTRIS® techique	NR	80 participants	NR	Full publication	**3m**

		COMPLICATIONS					
		**Minor Complications**		**Major complicaitons**		**Recurrences**	
		**Endoscopic CTR**	**Open CTR**	**Endoscopic CTR**	**Open CTR**	**Endoscopic CTR**	**Open CTR**
**Agee 1992**		2 transient apraxia	2 wound dehiscence<br>1 flexor tendons bowstringing	0	1 injury to the deep motor bran		
	total	**2**	**3**	**0**	**1**	**2**	**0**
**Aslani 2012**		1 pillar pain resolved<br>*scar problems possibly measured but not reported*	0	0	0		
	total	**1**	**0**			**0**	**0**
**Atroshi 2009**		5 patients reporting mild scar o	8 patients reporting mild scar o	5 patients reporting moderate (	3 patients reporting moderate or severe scar or palm pain at 5 years		
	total	**5**	**8**	**5**	**3**	**10** recurrence defined as deterio	**9** recurrence defined as deterio
**Benedetti 1996 / Sennwald 1995**		5 paresthesias/hypesthesias	6 scar dysanesthesias<br>2 paresthesias/hypesthesias	1 algodystrophy resolved at 2w	2 paresthesias/hypesthesias (sustaining at latest follow up)<br>1 algodystrophy resolved at 6w		
	total	**5**	**8**	**1**	**3**	**0**	**0**
**Brown 1993**		1 numbness<br>1 paresthesia<br>1 wound haemartoma<br>1 moderate scar tenderness	12 moderate or severe scar tend	1 injury superficial palmar arch	0		
	total	***4***	***12***	***1***	***0***	***1 no improvement in symprom***	***2 no improvement in symproms***
**Dumortier 1995**		6 paraesthesia in 3rd web spac<br>*scar pain measured but not separately reported*	2 paraesthesia in 3rd web spac	1 algodystrophy finally resolvec	1 algodystrophy finally resolved		
	total	***6***	***2***	***1***	***1***	***NR***	***NR***
**Eighghorn 2003**		0	2 infections				
	total	**0**	**2**			**3**	**4**
**Ejiri 2012**		1 transient apraxia- resolverd b	0	1 probable common digital nerv	0		
	total	**1**	**0**	**1**	**0**	***NR***	***NR***
**Erdman 1994**		1 transient ulnar nerve paresth<br>2 scar (hypertrophy, tethering,	1 palmar cutaneous nerve<br>19 scar (hypertrophy, tethering, tenderness)<br>1 scar infection with hypert	0	0		
	total	**3**	**21**	**0**	**0**	**1**	**0**
**Ferdinand 2002**		1 persisting wound pain	1 persisting wound pain<br>1 superficial sensory nerve injury	0	0		
	total	**1**	**2**	**0**	**0**	**0**	**1**
**Foucher 1993**		1 algodystrophy	0	0	0		
	total	**1**	**0**	**0**	**0**	**NR**	**NR**
**Giele 2000**		3 3rd web neuropraxia	1 3rd web neuropraxia<br>2 wound dehincences and infections				
	total	**3**	**3**	**0**	**0**	**1 no symptomatic relief**	**1 no symptomatic relief**
**Hoefnagels 1997**				1 knife broken (new operation to remove knife)<br>1 increased hypesthesia leading to revision surgery			
		**0**	**0**	**2**	**0**	**1 unterwent OCTR in other hos**	**0**
**Incoll 2004**							
	total	**NR**	**NR**	**NR**	**NR**	**NR**	**NR**
**Jacobsen 1996**		3 transient numbness	1 infection	0	0		
	total	**3**	**1**	**0**	**0**	**NR**	**NR**
**Koskella 1996**		0	0	0	0		
	total	**0**	**0**	**0**	**0**	**1 incomplete release**	**0**
**Larsen 2013**		2 transient numbness<br>4 pillar pain	7 pillar pain	0	0		
	total	**6**	**7**	**0**	**0**	**NR**	**NR**
**Mac Dermid 2003**				0	0		
	total	**NR**	**NR**	**0**	**0**	**NR**	**NR**
**Malhotra 2007**		2 residual numbness	9 scar tenderness<br>4 residual numbness<br>2 local scarring<br>1 keloid	0	2 algodystrophy		
	total	**2**	**16**	**0**	**2**	**0**	**0**
**Saw 2003**		1 transient numbness<br>1 superficial wound infection	1 hyperesthaesia over scar area<br>1 superficial wound infection<br>1 superficial haematoma	0	0	1 incomplete release	1 persistance of symptoms
	total	**2**	**3**	**0**	**0**	**1**	**1**
**Schaefer 1996**		0	0	0	0		
	total	**0**	**0**	**0**	**0**	**NR**	**NR**
**Stark 1996**		1 paraesthesia<br>0 scar pain at 3 months<br>1 haematoma	1 paraesthesia<br>5 scar pain at 3 months				
	total	**2**	**6**	**0**	**0**	**NR**	**NR**
**Tian 2007**		12 scar tenderness	23 scar tenderness	0	0		
	total	**12**	**23**	**0**	**0**	**3 did not improve postoperativ**	**0**
**Trumble 2002**		0<br>Scar tenderness was measured, but only as a continuous outcome	0	0	2 algodystrophy		
	total	**0**	**0**	**0**	**2**	**0**	**1 persistent symptoms**
**Tüzüner 2008**							
	total	**NR**	**NR**	**NR**	**NR**	**NR**	**NR**
**Werber 1996**		2 paresthesias in the ulnar nerv	0	0	0		
	total	**2**	**0**	**0**	**0**	**NR**	**NR**
**Westphal 2000**		3 scar tenderness at 3 months	3 scar tenderness at 3 months	0	0		
	total	**3**	**3**	**0**	**0**	**NR**	**NR**
