# Supplementary material for: Endoscopic and Open Release Similarly Safe for the Treatment of Carpal Tunnel Syndrome. A Systematic Review and Meta-Analysis
Source: PLoS One. 2015 Dec 16;10(12):e0143683. doi: 10.1371/journal.pone.0143683 (PMC4682940; doi:10.1371/journal.pone.0143683)
Supplement: S2 Table — (DOCX) [file pone.0143683.s009.docx]

| **Safety of CTS surgical treatments** | | | | | | |
| --- | --- | --- | --- | --- | --- | --- |
| **Patient or population:** patients with carpal tunnel syndrome **Intervention:** ECTR **Comparison:** OCTR | | | | | | |
| **Outcomes** | **Illustrative comparative risks* (95% CI)** | | **Relative effect (95% CI)** | **No of Participants (studies)** | **Quality of the evidence (GRADE)** | **Comments** |
|  | Assumed risk *(total event rate)* | Corresponding risk |  |  |  |  |
|  | **OCTR** | **ECTR** |  |  |  |  |
| **Recurrence** cases with reccurence of symptoms | **24 per 1000** | **24 per 1000** (13 to 44) | **OR 1.02**  (0.55 to 1.9) | 1684 (15 studies) | ⊕⊕⊝⊝ **low**^1,2^ |  |
| **Reoperations** cases with reoperations due to treatment failure | **11 per 1000** | **17 per 1000** (9 to 33) | **RR 1.53**  (0.78 to 2.98) | 1596 (16 studies) | ⊕⊝⊝⊝ **very low**^1,2^ |  |
| **Major complications** events with major complications | **10 per 1000** | **10 per 1000** (4 to 22) | **OR 1**  (0.44 to 2.27) | 2565 (25 studies) | ⊕⊕⊝⊝ **low**^1,2^ |  |
| **Complex regional pain syndrome (CRPS) / Major complications** events with Sudeck | **6 per 1000** | **3 per 1000** (1 to 12) | **OR 0.58**  (0.15 to 2.21) | 2343 (24 studies) | ⊕⊕⊝⊝ **low**^1,2^ |  |
| **Minor complications** events with minor complications | **103 per 1000** | **54 per 1000** (34 to 86) | **OR 0.50**  (0.31 to 0.82) | 2442 (24 studies) | ⊕⊕⊝⊝ **low**^2^ |  |
| **Transient neuropraxia /Minor complications** events with transient neuropraxia | **10 per 1000** | **23 per 1000** (12 to 45) | **OR 2.42**  (1.22 to 4.8) | 2182 (22 studies) | ⊕⊕⊝⊝ **low**^1,2^ |  |
| **scar problems / Minor complications** events with scar problems | **110 per 1000** | **29 per 1000** (18 to 47) | **OR 0.24**  (0.15 to 0.4) | 1943 (19 studies) | ⊕⊕⊝⊝ **low**^1,2^ |  |
| **Total complications** events with complications (minor or major) | **122 per 1000** | **76 per 1000** (50 to 113) | **OR 0.59**  (0.38 to 0.92) | 2442 (24 studies) | ⊕⊕⊝⊝ **low**^1,2^ |  |
| **Time to return to work** Time to return to work or to evey day activities |  | The mean time to return to work in the intervention groups was **9.56 lower** (12.51 to 6.6 lower) |  | 1115 (13 studies) | ⊕⊝⊝⊝ **very low**^1,3^ |  |
| *The basis for the **assumed risk** is the total even of all included studies .The **corresponding risk** (and its 95% confidence interval) is based on the assumed risk in the comparison group and the **relative effect** of the intervention (and its 95% CI).  **CI:** Confidence interval; **RR:** Risk ratio; **OR:** Odds ratio; | | | | | | |
| GRADE Working Group grades of evidence **High quality:** Further research is very unlikely to change our confidence in the estimate of effect.  **Moderate quality:** Further research is likely to have an important impact on our confidence in the estimate of effect and may change the estimate. **Low quality:** Further research is very likely to have an important impact on our confidence in the estimate of effect and is likely to change the estimate. **Very low quality:** We are very uncertain about the estimate. | | | | | | |
| ^1^ High Risk of Bias in included studies ^2^ Low number of events ^3^ Low sample size | | | | | | |
